# On-The-Fly Kinetics of the Hydrogen Abstraction by Hydroperoxyl Radical: An Application of the Reaction Class Transition State Theory

**DOI:** 10.3389/fchem.2021.806873

**Published:** 2022-01-31

**Authors:** Maciej Baradyn, Artur Ratkiewicz

**Affiliations:** Department of Chemistry, University of Bialystok, Bialystok, Poland

**Keywords:** reaction class transition state theory, hydroperoxyl radical, quantum tunneling composite, DFT, canonical variational theory

## Abstract

A Reaction Class Transition State Theory (RC-TST) is applied to calculate thermal rate constants for hydrogen abstraction by OOH radical from alkanes in the temperature range of 300–2500 K. The rate constants for the reference reaction C_2_H_6_ + ∙OOH → ∙C_2_H_5_ + H_2_O_2_, is obtained with the Canonical Variational Transition State Theory (CVT) augmented with the Small Curvature Tunneling (SCT) correction. The necessary parameters were obtained from M06-2X/aug-cc-pVTZ data for a training set of 24 reactions. Depending on the approximation employed, only the reaction energy or no additional parameters are needed to predict the RC-TST rates for other class representatives. Although each of the reactions can in principle be investigated at higher levels of theory, the approach provides a nearly equally reliable rate constant at a fraction of the cost needed for larger and higher level calculations. The systematic error is smaller than 50% in comparison with high level computations. Satisfactory agreement with literature data, augmented by the lack of necessity of tedious and time consuming transition state calculations, facilitated the seamless application of the proposed methodology to the Automated Reaction Mechanism Generators (ARMGs) programs.

## 1 Introduction

The crucial initialization step of the combustion of hydrocarbons is the H abstraction (Walker and Morley, 1997; Handford-Styring and Walker, 2002; Scott and Walker, 2002; Aguilera-Iparraguirre et al., 2008; Battin-Leclerc et al., 2013; Shi, 2018; Curran, 2019; Hashemi et al., 2019). Metatheses by atoms (i.e., ∙O, ∙H) (Saeys et al., 2003, 2006; Battin-Leclerc et al., 2013; Hou and You, 2017; Hashemi et al., 2019) and simple radicals (i.e., ∙OH(Chen et al., 2004; Battin-Leclerc et al., 2013; Edwards et al., 2014; Semenikhin et al., 2017; Frenklach et al., 2018; Gao et al., 2018; Wang et al., 2019), CH_3_ (Battin-Leclerc et al., 2013; Li et al., 2015; Mai et al., 2018),∙OOH (Handford-Styring and Walker, 2002; Carstensen and Dean, 2005; Battin-Leclerc et al., 2013; De Oliveira et al., 2016; Curran, 2019) are especially known to be the most significant channels for petrol depletion, thus mathematical combustion models are strongly sensitive to their rate constants (Semenikhin et al., 2017). H abstraction reactions are also important in consideration of adsorption and desorption at interstitial voids generated during molecular aggregation in solid state as well as investigation of possible bond formation between ‘noble’ elements in the periodic table, which are connected to the dissociation channels and internal isomerization processes (Jana et al., 2017, 2018; Pan et al., 2017). For some lighter systems, initialization with hydroperoxyl radical OOH (Hashemi et al., 2019) is believed to be a rate controlling step of the low temperature ignition. Processes belonging to the title family are also essential in the combustion of biofuels. Furthermore, it is known (Walker and Morley, 1997; Curran, 2019) that some peculiarities in the chemistry of combustion systems, such as NTC (Negative Temperature Coefficient) are mainly governed by reactions reducing the significance of the chain branching processes in favor of the termination processes. Typically, peroxy (mainly hydroperoxyl OOH) radicals (Blocquet et al., 2013) are involved at this stage. Because the title reaction family stands as a significant channel of decay of the OOH radicals, its accurate kinetic parameters are needed not only to quantify the initial stages of combustion, but also to properly predict the fate of the peroxy compound species. Unfortunately, despite its significance, only a very little amount of empirical kinetic data are available for the title reaction class. Due to the recombination of the peroxyl radicals and other side reactions, the direct rate measurements are very difficult, if possible at all. The only experimental data available are indirect measurements of the H-abstractions from propane and cyclopentane, reported by Walker et al. (Handford-Styring and Walker, 2002). The lack of experimental reports is partially compensated by computations.

A systematic study of the H abstractions by different peroxy radicals was carried out by Carstensen et al. (Carstensen and Dean, 2005, 2007. The rate constants were presented and analyzed for systematic trends in reactivity. Hashemi et al. (Hashemi et al., 2019) utilized the Classical Transition State Theory (CTST) data with 1D tunneling correction methodology for reactions of hydroperoxyl with propane. A similar series of CTST results were used for alkanes up to butane but results were obtained with a different electronic structure calculation methodology, as previously reported in Aguilera-Iparraguirre et al. (2008). Since the combustion models are intended for a wide range of possible fuels, a number of rates for each reaction class are needed. This is a challenging task, especially if the model is to be created with automated reaction mechanism generators (ARMG’s). However, as pointed out above, it is unrealizable to gain trustworthy data for so many reactions by experiments or explicit calculations, even with the simple CTST methodology. A software tool, capable of easy generation of reliable rates for any processes within a given family needs to be utilized to achieve this goal. The Reaction Class Transition State Theory (RC-TST) provides an accurate theoretical framework. By successful application to numerous reaction classes (Ratkiewicz et al., 2016), including also H abstractions, it has proven to be an effective and time efficient procedure for *on-the-fly* prediction of the thermal rate constants in a wide temperature range. In this study, an RC-TST framework was employed to derive the kinetic parameters necessary for the estimation of the rate constants of any reaction belonging to the alkane + ∙OOH → alkyl radical + H_2_O_2_ reaction family. To do this, explicit expressions relating to the rate constants of the reference reaction and those of other reactions in the class (called RC-TST correlations hereafter) have to be found. The assumption is, that these correlations apply to the whole title family. To compute the RC-TST parameters, 24 reactions are considered as a representative (training) set. Among these, eight occur at primary carbon active sites (type *p*), 10 at secondary (type *s*), and five at tertiary (type *t*). The simplest process R_1_ in the whole set is denoted as reference reaction. All reactions forming the training set, specified as SMILES (Weininger, 1988) strings, are explicitly listed in Table 1 below. SMILES specification rules are generally quite simple and well recognized in the chemical community. To further clarify this way of coding, a schematic representation of example *p*, *s* and *t* type H abstractions (namely processes R_11_, R_13,_ and R_12_) are available in the Supporting Information (Supplementary Figure S1).

**TABLE 1:** Processes selected to the representative (training) set for the alkyl + ∙OOH → alkyl radical + H_2_O_2_ reaction class; “*p*” represents H abstraction by∙OOH radical from a primary C atom (type *p*), “*s*” and “*t*” from secondary and tertiary ones. Both reactants and products are represented with SMILES linear notation.

No	Reaction				
R_1_ (*p*)	CC	+ **∙**OOH	→	**∙**CC	+ H_2_O_2_
R_2_ (*p*)	CCC	+ **∙**OOH	→	**∙**CCC	+ H_2_O_2_
R_3_ (*s*)	CCC	+ **∙**OOH	→	C**∙**CC	+ H_2_O_2_
R_4_ (*p*)	CCCC	+ **∙**OOH	→	**∙**CCCC	+ H_2_O_2_
R_5_ (*s*)	CCCC	+ **∙**OOH	→	CC**∙**CC	+ H_2_O_2_
R_6_ (*p*)	CC(C)C	+ **∙**OOH	→	**∙**CC(C)C	+ H_2_O_2_
R_7_ (*t*)	CC(C)C	+ **∙**OOH	→	C**∙**C(C)C	+ H_2_O_2_
R_8_ (*p*)	CCCCC	+ **∙**OOH	→	**∙**CCCCC	+ H_2_O_2_
R_9_ (*s*)	CCCCC	+ **∙**OOH	→	C**∙**CCCC	+ H_2_O_2_
R_10_ (*s*)	CCCCC	+ **∙**OOH	→	CC**∙**CCC	+ H_2_O_2_
R_11_ (*p*)	CC(C)CC	+ **∙**OOH	→	**∙**CC(C)CC	+ H_2_O_2_
R_12_ (*t*)	CC(C)CC	+ **∙**OOH	→	C**∙**C(C)CCC	+ H_2_O_2_
R_13_ (*s*)	CC(C)CC	+ **∙**OOH	→	C**∙**CC(C)C	+ H_2_O_2_
R_14_ (*p*)	CC(C)CC	+ **∙**OOH	→	**∙**CCC(C)C	+ H_2_O_2_
R_15_ (*p*)	CC(C) (C)C	+ **∙**OOH	→	**∙**CC(C) (C)C	+ H_2_O_2_
R_16_ (*p*)	CCCCCC	+ **∙**OOH	→	**∙**CCCCCC	+ H_2_O_2_
R_17_ (*s*)	CCCCCC	+ **∙**OOH	→	C**∙**CCCCC	+ H_2_O_2_
R_18_ (*s*)	CCCCCC	+ **∙**OOH	→	CC**∙**CCCC	+ H_2_O_2_
R_19_ (*t*)	CC(C)CCC	+ **∙**OOH	→	C**∙**C(C)CCC	+ H_2_O_2_
R_20_ (*s*)	CC(C)CCC	+ **∙**OOH	→	CC(C)**∙**CCC	+ H_2_O_2_
R_21_ (*t*)	CCC(C)CC	+ **∙**OOH	→	CC**∙**C(C)CC	+ H_2_O_2_
R_22_ (*s*)	CCCCCCC	+ **∙**OOH	→	C**∙**CCCCCC	+ H_2_O_2_
R_23_ (*t*)	CC(C)CCCC	+ **∙**OOH	→	C**∙**C(C)CCCC	+ H_2_O_2_
R_24_ (*s*)	CC(C)CCCC	+ **∙**OOH	→	CC(C)**∙**CCCC	+ H_2_O_2_

## 2 Methodology

### 2.1 Reaction Class Transition State Theory

Since the specifics of the RC-TST methodology have been detailed in previous reports (Truong, 2000; Ratkiewicz et al., 2016), only the most important features are mentioned here. All processes with the same common structural denominator, also known as reactive moiety, form a class. This definition is ambiguous since it depends on the specification of the reactive moiety. The approach profits by the similarity of reaction centers (moieties) within a given class, thus the discrepancies between rates are mainly attributed to alterations in the interactions of the reactive moiety with substituents. The unknown rate of any process within a family *k*(*T*) is obtained by capturing the difference between this process and the reference one (R_1_), which rate constant is well-known. Mathematically, both rates are related by a simple, temperature dependent, coefficient *f*(*T*):

$$k\left(T\right)=f\left(T\right)\times k_{ref}\left(T\right)$$ (1)

The reference reaction may be (but it is not always a case) tantamount to the principle reaction, which is the simplest reaction within a given family. Its rate constants, taken either from accurate high level calculations or directly from experiment, is well known. Under the TST framework, the coefficient *f(T*) is divided into five components (called further factors), which are the ratios of the corresponding quantities of the currently investigated reaction to the reference one, reflecting the particular components of the Classical Transition State Theory (CTST) (Bao and Truhlar, 2017) formula:

$$f\left(T\right)=f_{\sigma }\times f_{V}\left(T\right)\times f_{\kappa }\left(T\right)\times f_{Q}\left(T\right)\times f_{HR}\left(T\right)$$ (2)

**FIGURE 1:**
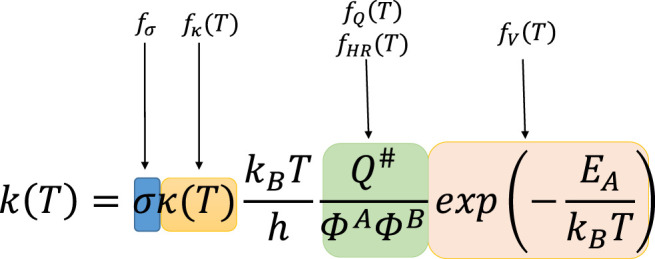
Classical Transition State Theory (CTST) formula with marked correspondences to RC-TST factors. In this formula, *σ* stands for symmetry number (reaction multiplicity); *T* is the temperature in Kelvins*; κ(Τ)* is the quantum tunneling coefficient; *k*_*B*_ and *h* are Boltzmann and Planck constants; *Q* ^#^, *Φ*_A,_ and *Φ*_B_ symbolize total partition functions of the transition state and both reactants; *E*_*A*_ is activation energy.

In the formula above, factor $f_{\sigma }$ corresponds to the symmetry number *σ*, and is the only temperature independent. $f_{V}\left(T\right)$ symbolizes the potential energy factor, $f_{\kappa }\left(T\right)$ denotes the tunneling factor, coefficient $f_{Q}\left(T\right)$ corresponds to the total partition function *Q* of the reactants and transition state. Finally, the $f_{HR}\left(T\right)$ (hindered rotations factor) takes into account the differences between the anharmonic motions (hindered rotations) of the arbitrary reaction (investigated) and the reference one. Accordingly, all the coefficients from the right side of Eq 2 are calculated as the ratios of the quantities corresponding to the arbitrary (investigated) reaction to these for the reference process (Ratkiewicz et al., 2016; Baradyn and Ratkiewicz, 2020). The strict definitions of the five factors from Eq 2 are given in the references above, they are also provided in the Supporting Info (Supplementary Figure S2). The correspondences between particular RC-TST factors and the CTST formula are detailed in Figure 1 below.

Despite the utilization of the original TST framework, there are inherent dissimilarities between TST and RC-RST methodologies. Whereas TST (CTST) determines the absolute rate constants, the RC-TST may be considered as an extrapolation of the (well known) rate constants of the reference reaction to any other class member. The reference reaction (R_1_) rate come from experiment or high level VTST (Variational TST) computations. As such, it accounts for variable dividing surface, quantum tunneling, anharmonities of the low frequency motions, etc. The RC-TST formalism transfers the factor variability to reaction rates. Thus, the unknown arbitrary rate in the same family is determined by capturing variations of particular factors from the reference process to the investigated one. This makes a significant difference when compared to the classical TST rate–the RC-TST transfers all the properties already included in the rate of the reference reaction to any other process within a given class, which is not a case in TST calculations. Factors from Eq 2 are calculated for all the reactions forming the training set (see Table 1) and averaged and fitted to RC-TST correlations. To obtain the potential energy factor *f*_*Q*_(*T*), classical reaction barriers are needed. For processes R_1_-R_24_ they are calculated directly. Of course, to do this transition states (TS’s) structures are necessary. All of the geometries and frequencies are available in the Supporting Information of this article (Supplementary Tables S1−23). This is the most problematic point of the whole procedure. Whereas automated construction (and optimization) of transition states and *on-the-fly* accurate kinetic calculations are now possible (Carstensen and Dean, 2009; Gao et al., 2016; Panadés-Barrueta et al., 2019; Van de Vijver et al., 2019; Li et al., 2020; Pattanaik et al., 2020), this is still an emerging technology developed for only a few reaction classes, thus its simple implementation is not straightforward. It has been shown previously (Ratkiewicz et al., 2016) that direct barrier calculations may be omitted by approaching them by simple linear expressions. In this approximation, referred to as RC-TST/LER (Linear Energy Relationship), the classical reaction barrier height $\Delta V^{\#}$ for the arbitrary reaction is retrieved with the Linear Energy Relationship (LER) between the classical barrier and reaction energy, comparable to the Evans−Polanyi relationship. The rate constants of any reactions in the title class can be predicted either from the LER or BHG (Barrier Height Grouping) methodologies, which are discussed in further detail later in this study. Whereas the first one requires the reaction energy and symmetry number, only the symmetry number is needed in the latter. In any case, no explicit transition state (TS) and/or frequency calculations are necessary, which makes it practical for the *on-the-fly* reaction rates computations, utilized in the Automated Reaction Mechanism Generation (ARMG) programs (Carstensen and Dean, 2009; Magoon and Green, 2013; Van de Vijver et al., 2015; Li et al., 2020).

### 2.2 Computational Details

All electronic structure calculations were performed on Gaussian 16 software (Frisch et al., 2016). A hybrid meta generalized gradient approximation exchange-correlation functional M06-2X (Zhao and Truhlar, 2008), which is intended especially for the chemical kinetics, was utilized to calculate the RC-TST factors. The cc-pVTZ basis set was chosen as it works significantly better than the simpler cc-pVDZ. To account for the exorbitant electron density on the OOH radical, the diffusion functions were employed. Taking into account the above discussion, the M06-2X/aug-cc-pVTZ theory level was chosen. The accuracy of this methodology has been proven by the benchmark calculations of the Potential Energy Surface (PES) of the reference reaction, reported further in this study. It is important to stress out, that all quantities needed in the RC-TST methodology are only relative to the reference process R_1_. As such, a relatively low level of theory, appropriate for the ARMG schemes, may be utilized with an acceptable outcome. All reported results were obtained for the lowest energy conformers, no constraints were imposed during the geometry optimizations. Vibrational analysis was undertaken for all the processes considered to ensure that all minima had no imaginary frequency, whereas transition state structures show one and only one imaginary frequency, matching the reaction coordinate. The calculated energies, geometries, and frequencies were then used to derive the RC-TST correlations, approximating particular factors. To obtain the data needed to derive the tunneling $f_{\kappa }(T$ ), partition function $f_{Q}(T$ ) and hindered rotation $f_{HR}(T$ ) factors, the CTST calculations with 1D Eckart tunneling corrections for all the reactions within the representative training set were performed with the MSMC (Duong et al., 2015) and TheRate (Duncan et al., 1998) codes. Harmonic oscillator (HO) approximation was used to approximate the vibrational modes, except for low frequency vibrations, treated by the direct solution of the 1D Schrödinger equation, as implemented in the MSMC program (Duong et al., 2015). Appropriate potential energy curves were obtained with the relaxed scans by using discrete steps of 10. For transition states, the C … H and O … H bonds were frozen during the scans. It is important to stress that the sole purpose of this approach was to prevent the relaxation of reactants/products during the scans and that no geometry constraints were imposed during the optimization of the transition states of the reactions R_1_–R_24_.

Thermal rate constants were obtained for the 300–2500 K temperature regime. Since the most accurate rate was needed for reference reaction R_1_, the Canonical Variational Transition State Theory (CVT) with the Small Curvature Tunneling (SCT) method was utilized, as implemented in the POLYRATE 17c (Zheng et al., 2017) program. To model vibrations transverse to the reaction path, physically intuitive curvilinear internal coordinates (keyword CURV3 in POLYRATE) were utilized.

## 3 Results and Discussion

This section starts by discussing calculations of the rate constant of the reference reaction. The RC-TST correlations, approximating particular factors, were then derived. To assess the reliability of these, three independent error analyses are presented.

### 3.1 Reference Reaction

Since the RC-TST rates of any representative of the title reaction family may be considered as an extrapolation of the reference process, exhaustive knowledge with the best accuracy possible is of crucial importance. For the title class, the smallest possible (principal) representative is the H abstraction from methane CH_4_ + ·OOH → ∙CH_3_ + H_2_O_2_. Although the simplest and, consequently, the less computationally demanding reaction within a whole class, the principal reaction is not always the best reference. Methane is known to possess unusual stability (and–consequently–high reaction barrier) due to its high symmetry and lack of the C-C bond. This results in possible problems with the extrapolation of its rate to the other class representatives. This expectation was confirmed by our calculations, as well as by results previously reported in Aguilera-Iparraguirre et al. (2008), where the barrier for CH_4_ + ·OOH → ∙CH_3_ + H_2_O_2_ reaction is higher by about 4.5-5 kcal/mol than that for reaction with C_2_H_6_ (R_1_) for all theory levels employed. The process R_1_ is the smallest, with all characteristic elements of the title class, i.e. the reactive moiety OOHH and the C-C bond of the alkyl group. For this reason, the C_2_H_6_ + ·OOH → ∙C_2_H_5_ + H_2_O_2_ (R_1_) reaction was chosen as the reference, as discussed further later in this article.

#### 3.1.1 Potential Energy Surface

**FIGURE 2:**
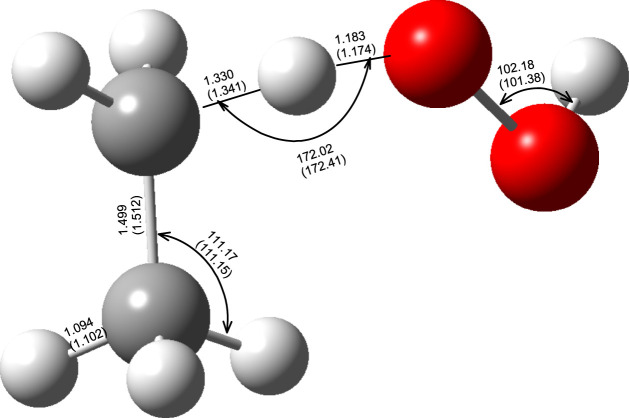
Optimized geometries (distances in Å and angles in degrees) of the reactant transition state of the C_2_H_6_ + ·OOH → ∙C_2_H_5_ + H_2_O_2_ (R_1_) reaction at the M06–2X/aug-cc-pVTZ and QCISD/aug-cc- pVDZ (numbers in parenthesis).

The optimized M06–2X/aug-cc-pVTZ level of theory geometrical parameters of the reactants and the TS of the C_2_H_6_ + ·OOH → ∙C_2_H_5_ + H_2_O_2_ reaction are shown in Figure 2. As mentioned in the previous section, the transition state was confirmed to possess only one imaginary frequency corresponding to the H transfer between ethane and hydroxyl radical. The geometry parameters, computed at the higher level of theory QCISD/aug-cc-pVDZ, are also presented for the sake of comparison. These results demonstrate that, for the transition state, the prediction of both methods was quite similar, so there is no benefit in using the computationally demanding QCISD/aug-cc-pVDZ methodology.

The classical and zero point energy corrected barriers calculated at various levels of theory, along with literature values (Aguilera-Iparraguirre et al., 2008), are listed in Table 2. Amongst the various methods considered here, the CBS-QB3, CBS-APNO, and W1U composite methods are known for their precision compared to experimental data, thus their results were expected to yield the most accurate and comprehensive assessments. It is seen, that M06-2X results are in a good agreement with more time consuming composite chemistries. The M06-2X reaction barriers are almost independent from the basis sets utilized. The value (∼18 kcal/mol) is almost the same as the CBS-QB3 results. It is interesting to observe that reaction energies depend on the basic set considerably more than the barriers do. As the BH and HLYP density functional frequently used in our previous studies (Ratkiewicz et al., 2016) seem to overestimate the reaction barrier for this reaction class, they were not utilized here. For both reaction energies and barrier heights, the M06-2X/aug-cc-pVTZ energies are in the best agreement with these resulting from composite methods. For that reason, this theory level is a method of choice for the title reaction family.

**TABLE 2:** Calculated classical (*V*_*C*_) and zero point corrected ( $V_{g}^{a}$ ) barriers for the C_2_H_6_ + ·OOH → ∙C_2_H_5_ + H_2_O_2_ (numbers are in kcal/mol).

Method	ΔE	ΔE ZPE	ΔV	ΔV ZPE
M06-2X/cc-pVDZ	19.1	17.3	20.2	18.0
M06-2X/aug-cc-pVDZ	16.7	14.6	20.2	18.0
M06-2X/cc-pVTZ	17.1	14.8	20.7	18.3
M06-2X/aug-cc-pVTZ	16.3	14.1	20.9	18.3
M06-2X/6-311++G (d,p)	16.5	14.3	20.5	18.2
B97D3/DEF2TZVP	19.9	17.7	16.4	13.8
BH&HLYP/cc-pVDZ	21.5	19.2	25.7	23.2
BH and HLYP/aug-cc-pVDZ	18.3	16.1	25.6	23.1
B3LYP/CBSB7	20.2	17.9	20.4	17.9
B3LYP/6-311++G (2d,2p)	18.0	15.8	20.6	18.0
CAM-B3LYP/6-31G(d)	24.9	22.5	24.5	21.8
MP2/cc-pVDZ//M06-2X/aug-cc-pVTZ	16.5	14.8	23.0	20.8
MP2/aug-cc-pVDZ//M06-2X/aug-cc-pVTZ	11.7	9.9	18.7	16.5
MP2/cc-pVTZ//M06-2X/aug-cc-pVTZ	12.7	11.0	20.4	18.2
MP2/aug-cc-pVTZ//M06-2X/aug-cc-pVTZ	11.5	9.7	19.1	16.9
MP4/cc-pVDZ//M06-2X/aug-cc-pVTZ	19.4	17.7	24.7	22.5
MP4/aug-cc-pVDZ//M06-2X/aug-cc-pVTZ	14.8	13.0	20.1	17.8
MP4/cc-pVTZ//M06-2X/aug-cc-pVTZ	28.1	26.3	21.9	19.6
MP4/aug-cc-pVTZ//M06–2X/aug-cc-pVTZ	14.5	12.8	20.4	18.2
CCSD(T)/cc-pVDZ//M06-2X/aug-cc-pVTZ	20.4	18.7	25.2	22.9
CCSD(T)/aug-cc-pVDZ//M06–2X/aug-cc-pVTZ	16.5	14.7	21.4	19.2
CCSD(T)/cc-pVTZ//M06-2X/aug-cc-pVTZ	17.4	15.7	23.1	20.9
CCSD(T)/aug-cc-pVTZ//M06–2x/aug-cc-pVTZ	16.5	14.7	22.0	19.8
G4		14.0		19.1
CBS-QB3		14.0		17.8 Aguilera-Iparraguirre et al. (2008)
CBS-APNO		14.1		19.5 Aguilera-Iparraguirre et al. (2008)
W1U		13.8		19.9

**FIGURE 3:**
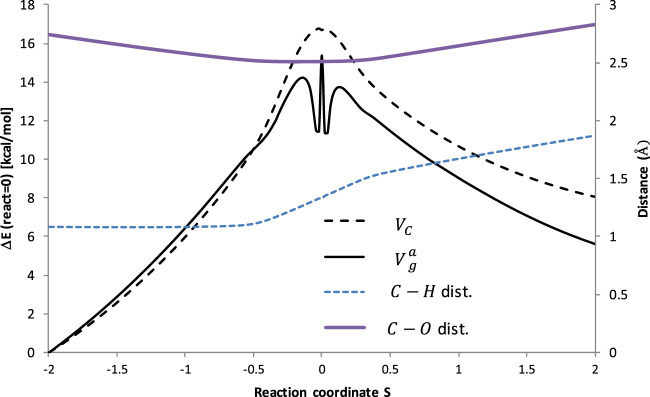
Potential energy and bonds lengths along the reaction coordinates of the reaction R_1_ (C_2_H_6_ + ·OOH → ∙C_2_H_5_ + H_2_O_2_). *V*_*C*_ is the classical adiabatic ground-state potential, whereas $V_{g}^{a}$ symbolizes the vibrationally adiabatic ground-state potential energy curve. H atom is transferred from C to O (see Figure 2 above).

Figure 3 illustrates the potential energy surface along the reaction path. To ensure the convergence of the Small Curvature Tunneling calculations classical adiabatic ground state potential *V*_*c*_ and the zero-point vibrational energy, ZPVE, were computed at the M06–2X/aug-cc-pVTZ theory level at 200 points along the Minimum Energy Path (MEP), 100 points of each side. The vibrationally adiabatic ground-state potential $V_{g}^{a}$ is the sum of the two terms: *V*_*c*_ + ZPVE. Both potentials (relative to reactants) along with the C-H and C-O distances (see Figure 2) as a function of reaction coordinate are pictured in Figure 3. Plots illustrating both potentials as the function of the aforementioned bond distances are available in the Supporting Information (Supplementary Figures S3A,B).

It is interesting to observe that ZPVE lowered the barrier to about 2 kcal/mol, corresponding to ∼10% of the total barrier height, which is typical for the H abstractions. However, a significant drop (approximately the next 2 kcal/mol) of the ZPVE in the vicinity of the transition state occurred, which is unusual. The nature of this phenomenon is not considered here and may require further investigation. The distance between the transferred H atom and C abstraction site increases continuously with the course of the reaction, although it happens noticeably faster in the vicinity of the transition state. The C-O distance shortens when approaching TS, but increases after a critical point.

#### 3.1.2 Rate Constants

**FIGURE 4:**
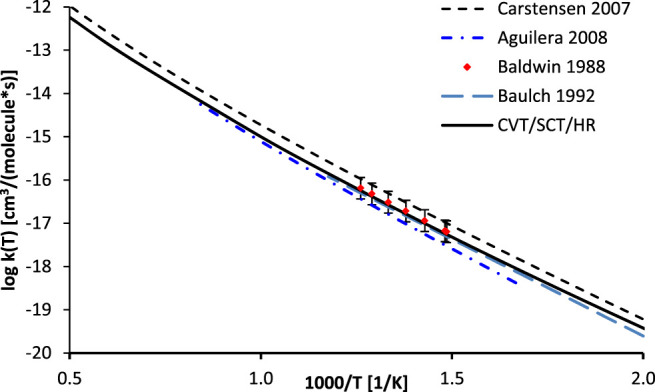
Arrhenius plot of the calculated rate constants for the reaction R_1_ (C_2_H_6_ + ·OOH → ∙C_2_H_5_ + H_2_O_2_) along with literature data.

As mentioned, the high pressure limit of the thermal rate constants of the reaction R_1_ was computed with the CVT/SCT methodology in curvilinear (CURV3 keyword in POLYRATE) coordinates, based on bonds stretches, bends, and torsions. Harmonic vibrational frequencies were calculated at 200 points along the MEP. In accordance with the methodology of Fernández-Ramos et al. (2007), to account for the quantity of the symmetrically equivalent reaction paths, a symmetry number of six was used. The low frequency vibrational modes, corresponding to rotations of the -CH_3_ groups, as well as H rotation along the O-O bonds in the OOH group were explicitly treaded as hindered (internal) rotations. The results, along with the available literature data, are plotted in Figure 4. It was found that the SCT tunneling factor (*κ*_*SCT*_ = 15.71) differs only slightly from the 1D Eckart value (*κ*_*Eckart*_ = 14.26). This result confirms the validity of the 1D tunneling approach utilized in previous reports. Consequently, the CVT/SCT/HR and Eckart/HR rates are very close one to another and their separate plots would be hardly distinguishable. The CVT/SCT/HR rate is slightly larger than those computed by Aguilera-Iparraguirre (Aguilera-Iparraguirre et al., 2008) as well as those recommended by Baulch et al. (Baulch et al., 1992), but is significantly smaller than those proposed by Carstensen et al. (Carstensen and Dean, 2007) in the whole temperature range. To the best of our knowledge, there are no direct measurements of the rates of reaction R_1_. Only indirect (i.e. based on the reaction with methane) data reported by Baldwin et al. (Baldwin et al., 1988) are available and are in excellent agreement with our calculations. In general, the computed CVT/SCT rates are in satisfactory agreement with available data, thus they can be safely utilized for estimation of the unknown rates of any process belonging to a title reaction class. This rate may be fitted to an Arrhenius expression as:

$$k_{CVT/SCT\;}\left(T\right)=5.22\times 10^{-25}\times T^{4.13}\times exp\left(\frac{-7206.9}{T}\right)\;\left(cm^{-3}s^{-1}molecule^{-1}\right)$$ (3)

### 3.2 RC-TST Parameters

In this section, the RC-TST factors are derived based on the electronic structure calculations for the whole representative set (see Table 1).

#### 3.2.1 Potential Energy Factor

**FIGURE 5:**
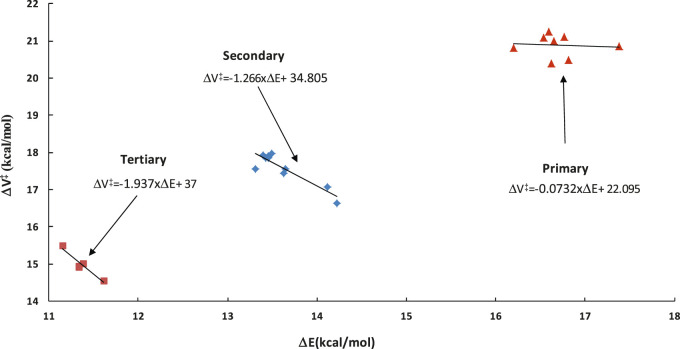
Linear energy relationship (LER) of the barrier heights ΔV versus reaction energies ΔE for the representative set. All data were obtained at the M06-2X/aug-cc-pVTZ level of theory.

This factor captures the differences between the barriers of particular class representatives. Since these differences are reflected in the exponential part of the TST formula (see Figure 1), even the small inaccuracies in barriers may significantly affect the calculated rates. As such, proper following of the barriers variability is essential. Transition states are needed to know the exact barrier. It was previously shown that within a given family, a linear relation between reaction energy ΔE and barrier height ΔV^#^ holds similar to the well-recognized Evans-Polanyi relationship. This relationship, referred to as LER (Linear Energy relationship), is utilized here to overcome the necessity of the on-the-fly TS calculations, which are difficult to perform and time consuming, if possible at all. Direct on-the-fly calculations of reaction energy are much faster and easier to achieve. Within the RC-TST framework, only relative barriers are needed to calculate *f*_*V*_, thus the relatively low level of theory can be safely utilized. To obtain these relative values, an exact barrier of the reference reaction R_1_ of 20.92 kcal/mol (see Table 3) is necessary. In this study, the LER is derived at the M06-2X/aug-cc-pVTZ level of theory. Table 3 reports the DFT reaction energies and barrier heights for all processes from the training set. The derived LER’s plotted with the reaction energies on ordinates are shown in Figure 5. As mentioned earlier, the principal reaction CH_4_ + ·OOH → ∙CH_3_ + H_2_O_2_ does not follow the same tendency as others, and thus was excluded from the LER. The linear fit leads to the following expressions (kcal/mol):

$$\text{Δ}V_{primary}^{†}=-0.0732\times \text{Δ}E+22.095\;\left(\text{H}\;\text{abstractions}\;\text{from}\;\text{primary}\;\text{C}\;\text{sites}\right)$$ (4a)

$$\text{Δ}V_{secondary}^{†}=-1.266\times \text{Δ}E+34.805\;\left(\text{H}\;\text{abstractions}\;\text{from}\;\text{secondary}\;\text{C}\;\text{sites}\right)$$ (4b)

$$\text{Δ}V_{tertiary}^{†}=-1.937\times \text{Δ}E+37.000\;\left(\text{H}\;\text{abstractions}\;\text{from}\;\text{tertiary}\;\text{C}\;\text{sites}\right)$$ (4c)

**TABLE 3:** Classical reaction energies *ΔE*, barrier heights $\Delta V^{‡}$ , and absolute deviations between the calculated barrier and those computed from LER expressions and barrier height grouping (BHG) approaches at 0K. Zero-point energy corrections are not included.

Rxn	Δ*E* DFT^a^	Δ*V* DFT^a^	Δ*V* DFT^b^	Δ*V* BHG^c^	|Δ*V* ^‡^ *–*Δ*V* ^‡^_*LER*_|^d^	
					DFT^b^	BHG^c^
**R**_**1**_	16.35	20.92	—	—	—	—
R_2_	16.60	21.25	20.88	20.87	0.37	0.38
R_3_	13.31	17.55	17.96	17.57	0.41	0.03
R_4_	16.65	20.99	20.88	20.87	0.12	0.12
R_5_	13.40	17.93	17.84	17.57	0.09	0.36
R_6_	16.81	20.49	20.86	20.87	0.37	0.38
R_7_	11.15	15.50	15.41	14.99	0.09	0.51
R_8_	16.76	21.11	20.87	20.87	0.24	0.24
R_9_	13.45	17.89	17.78	17.57	0.11	0.32
R_10_	13.65	17.54	17.53	17.57	0.01	0.03
R_11_	16.62	20.40	20.88	20.87	0.48	0.47
R_12_	11.38	15.01	14.94	14.99	0.07	0.02
R_13_	14.11	17.06	16.94	17.57	0.12	0.51
R_14_	16.20	20.80	20.91	20.87	0.10	0.07
R_15_	17.38	20.84	20.82	20.87	0.02	0.03
R_16_	16.54	21.09	20.88	20.87	0.20	0.22
R_17_	13.43	17.84	17.81	17.57	0.04	0.27
R_18_	13.62	17.44	17.56	17.57	0.12	0.13
R_19_	11.33	14.94	15.04	14.99	0.10	0.05
R_20_	14.22	16.64	16.81	17.57	0.17	0.93
R_21_	11.61	14.56	14.50	14.99	0.05	0.43
R_22_	13.45	17.86	17.77	17.57	0.08	0.29
R_23_	11.33	14.93	15.04	14.99	0.11	0.06
R_24_	13.50	17.97	17.72	17.57	0.24	0.39
		**MAD** ^d^			**0.16**	**0.23**	

Bolded numbers are the Mean Absolute Deviations (MAD’s).

a Calculated at aug-M06–2X/cc-pVTZ, level of theory.

b Calculated from the LER, using reaction energies calculated at aug-M06–2X/cc-pVTZ, level of theory: Eq 2a–c.

c Estimated from the BHG (Barrier Height Grouping) approximation.

d Mean absolute deviation (MAD) between the LER/BHG, approaches and the direct calculations.

As it is seen in Table 3, the absolute deviations between barriers, directly computed and fitted are smaller than 0.5 kcal/mol, whereas the average value (Medium Absolute Deviation – MAD) is less than 0.2 kcal/mol for the representative set of reactions. As can be seen from Figure 5, even though the actual relationship is not linear for primary sites, Eq 4a is still applicable. This is because its exclusive purpose is to approximate the real reaction barrier with satisfactory accuracy. The resulting errors (see Table 3) are smaller than the systematic error of the DFT calculations. As such, to keep the methodology consistent, the linear equations can be confidently used to predict the value of the potential energy factor *f*_*V*_, even when there is no observable linear relation between *ΔE* and *ΔV*. As shown further in this study (Section 3.4.1), such a treatment has a significant advantage over the simplified approximation, where only one averaged barrier is assigned to a specific reaction site. In general, the quality of the fitting equation for both LER and other factors, measured by *R* ^*2*^ and/or *F* statistics, is not crucial. As follows from both our experience and assessments done for this study, maximization of the fit quality statistics would lead to significantly more complicated equations with no real benefits to the accuracy of the final results, thus the simplest fits with reasonable accuracy are utilized.

Instead of the time consuming calculations of ΔE, the barrier of any reaction can be approximated by an average value for the same abstraction site (primary *p,* secondary *s,* and tertiary *t*). This approximation is called “Barrier Height Grouping” (BHG). It is known from previous studies that substitution of alkyl groups stabilizes the reaction site, thus lowering the barrier with an increasing number of substituents. Consequently, it is be expected that reactions occurring at primary sites show higher activation energy from those taking place at secondary C sites. Tertiary sites are thus likely to have the lowest barriers among all the types. Indeed, as can be seen from Figure 5 and Table 3, this rule also holds for the title family, with average barriers of 20.87, 17.57, and 14.99 kcal/mol for H abstractions from *p*, *s,* and *t* sites, respectively. Correspondingly, the average aberrations of barriers predicted from BHG are 0.24, 0.33, and 0.21 kcal/mol, which is 1.2, 1.9, and 1.5% of the average barriers in particular subclasses. As such, this method is also usable for quick estimation of the unknown barrier height with satisfactory error. The key benefit of this approach is its simplicity - no electronic structure calculations are needed to estimate rate constants. In general, it can be concluded that the unknown barrier height of any reaction within a title family can be obtained by application of either LER or BHG approximations; the estimated barrier is further used to compute the potential energy factor. It is important to point out that, in any case, no transition state calculations are necessary. The performance of this estimation is assessed further in this study.

#### 3.2.2 Symmetry Factor

The symmetry factor *f*_*σ*_ captures the variability of the number of indistinguishable reaction paths from the reference process R_1_ to any other within a title family. As the only one among all the RC-TST factors, it does not depend on temperature. Here, this number is tantamount to the amount of active reaction sites multiplied by a quantity of possible H atoms to be abstracted from a given site (3 for *p*, two for *s*, and one for *t* H abstraction sites). *f*_*σ*_ is simply computed as the ratio of reaction symmetry numbers of the unknown (arbitrary) and reference reaction (symmetry number = 6). For reactions with n-alkanes, the symmetry number is always equal to six for *p* type abstractions and 2*n, where *n* is the number of secondary *C* atoms*.* The values for the representative set are listed in Table 4.

**TABLE 4:** Symmetry number *f*_*σ*_ and tunneling *f*_*κ*_ RC-TST coefficients at T = 300K.

Reaction	Symmetry number factor	Tunneling ratio factor, $f_{\kappa }$			
		Eckart^a^	Fitting^b^	Deviation^c^	%Deviation^d^
R_1_	1	(14.26)^e^	—	—	—
R_2_	1.00	0.96	0.89	0.08	8.6
R_3_	0.33	0.95	0.85	0.10	11.3
R_4_	1.00	0.93	0.89	0.05	5.4
R_5_	0.67	0.96	0.85	0.11	13.1
R_6_	1.50	0.83	0.89	0.05	6.1
R_7_	0.17	0.86	0.81	0.05	6.4
R_7_	1.00	1.01	0.89	0.12	13.3
R_9_	0.67	1.02	0.85	0.17	20.0
R_10_	0.33	0.86	0.85	0.01	0.9
R_11_	0.50	0.73	0.89	0.16	17.6
R_12_	0.17	0.78	0.81	0.03	3.4
R_13_	0.33	0.78	0.85	0.07	8.3
R_14_	0.50	0.92	0.89	0.04	4.3
R_15_	2.00	0.77	0.89	0.12	13.0
R_16_	1.00	0.98	0.89	0.09	10.0
R_17_	0.67	0.99	0.85	0.14	16.8
R_18_	0.67	0.84	0.85	0.01	1.4
R_19_	0.17	0.73	0.81	0.08	9.9
R_20_	0.33	0.51	0.85	0.34	40.6
R_21_	0.17	0.51	0.81	0.30	37.1
R_22_	0.67	0.98	0.85	0.13	14.7
R_23_	0.17	1.28	0.81	0.08	10.2
R_24_	0.33	0.67	0.85	0.18	21.2
**MAD** ^e^ ^,^ ^f^				**0.11**	**12.8**

Bolded numbers are the Mean Absolute Deviations (MAD’s).

a Calculated directly using Eckart method with M06–2X/aug-cc-pVTZ, reaction barrier heights and energies.

b Calculated by using fitting expression.

c Absolute deviation between the fitting and directly calculated values.

d Percentage deviation (%).

e Medium absolute deviations (MAD) and deviation percentage between the fitting and directly calculated values.

f Tunneling coefficient calculated for reaction (R_1_) using the Eckart method with the electronic structure data obtained at M06–2X/aug-cc–pVTZ, level.

#### 3.2.3 Tunneling Factor *f*_*κ*_

It is well recognized, that tunneling is significant for the processes involving light particle transfer (Ratkiewicz et al., 2010; Alecu and Truhlar, 2011a; b; Awan et al., 2012; Sirjean et al., 2012). As can be seen from Figure 4, it is also important for the reference reaction of the title reaction class. The tunneling factor *f*_*κ*_, measuring the tunneling extent from reference reaction to other processes within the family, is defined as a ratio of the transmission coefficient of reaction investigated with that of the reference reaction R_1_. It was previously shown, that the one-dimension Eckart method properly follows the change of *κ* coefficients from reaction to reaction within the same class (Truong et al., 1999). To obtain the RC-TST correlation, the results for the training reaction set are fitted to analytical expressions. The tunneling coefficient depends on the reaction barriers, which group together into three sets, according to the nature of the reaction site (*p, s,* or *t*)*.* It is thus supposed, that processes belonging to the same set possess similar tunneling coefficients. Simple expressions approximating *f*_*κ*_ for *p, s,* and *t* active reaction sites were obtained by fitting to the computed averages and are given below:

fκ,pri=0.99−1.12×exp(−T169.7) for primary H abstraction sites (5a)

fκ,sec=0.99−0.94×exp(−T207.57) for secondary H abstraction sites (5b)

fκ,tert=0.98−0.98×exp(−T232.4) for tertiary H abstraction sites (5c)

**FIGURE 6:**
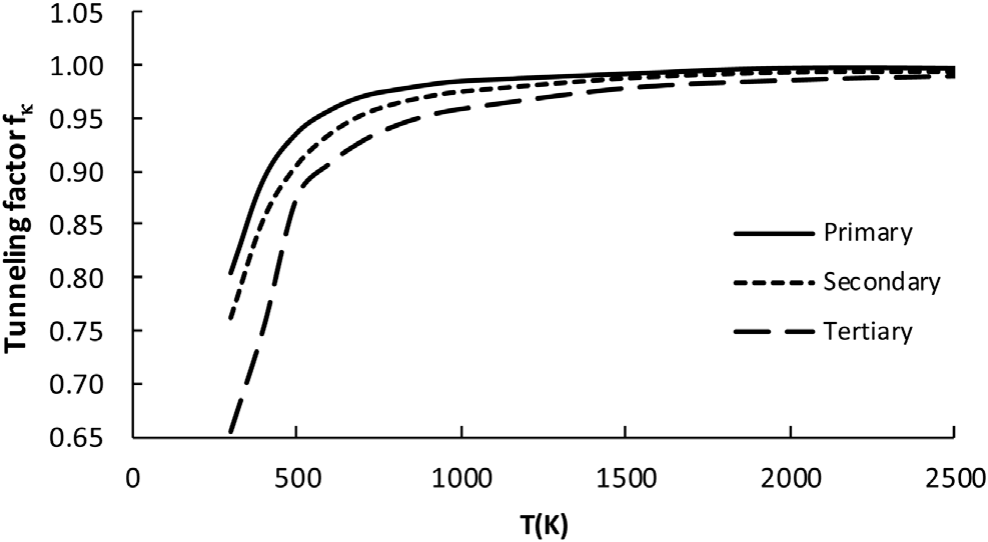
Plot of the tunneling factor (*f*_*κ*_) as a function of temperature for all 23 reactions considered in the representative set (R_2_–R_24_).

The three above equations are pictured in Figure 6. The factor values for T = 300K with error analysis are listed in Table 4. The division to three sets is reasonable for *f*_*κ*_. However, the errors (∼40% maximum and ∼13% averaged) are unexpectedly large when compared to similar reaction classes. For example, the average errors are about 5% for H abstraction by alkyl (Ratkiewicz et al., 2013) and 10% by hydroxyl (Huynh et al., 2006) radicals. This suggests the complicated nature of the tunneling phenomenon for the title family, which may be an interesting subject for further study. It is noted that, although the *κ* quantum tunneling coefficient *κ* (which should not be confused with tunneling factor *f*_*κ*_(*T*)) decreases with the rising T, the overall factor *f*_*κ*_, being the ratio of two *κ* coefficients, increases. Since the tunneling contributions to the rate constants lower with increasing temperature, the error also decreases. For T > 1000K the tunneling factor *f*_*κ*_, is almost equal to unity, thus it does not significantly affect the RC-TST rates.

#### 3.2.4 Partition Function Factor *f*_*Q*_

**FIGURE 7:**
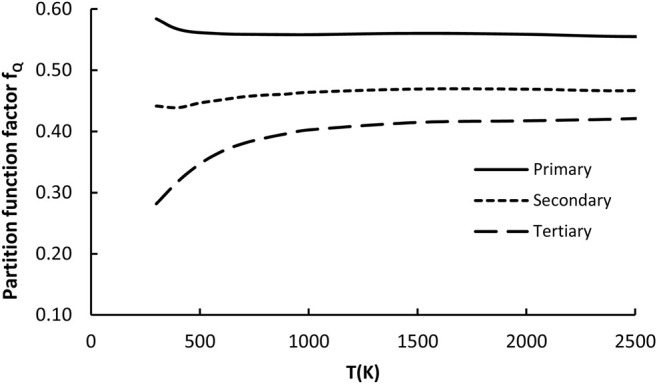
Plot of the partition function factor (*f*_*κ*_) as a function of temperature for all 23 reactions considered in the representative set (R_2_–R_24_).

This factor captures differences between total partition functions of the reference reaction and arbitrarily given family representative. The total partition function is the product of the electronic, vibrational (including part responsible for hindered rotations), rotational and translational components, where only two latest are independent on temperature. The others depend on T and originate mostly from the changes of coupling (associated with the vibrational component of Q including also internal rotations) between active reaction site (reactive moiety) and substituents. Since in this study the contributions from the low frequency motions (hindered rotations) are treated separately, they are not involved in the evaluation of the *f*_***Q***_ component. The averaged *f*_*Q*_ values for *p*, *s,* and *t* H abstractions were calculated and found to be almost temperature independent, but different to unity in the whole 300–2500K regime. For the sake of simplicity, they are approximated by constant expressions. The only exception from this rule is for H abstraction from *t* sites, where *f*_*Q*_ falls for T < 700K. Averaged values of the partition function factor are plotted in Figure 7 and fitted as follows:

$$f_{Q,pri}=0.56\;\text{for}\;\text{primary}\;\text{H}\;\text{abstraction}\;\text{sites}$$ (6a)

$$f_{Q,sec}=0.46\;\text{for}\;\text{secondary}\;\text{H}\;\text{abstraction}\;\text{sites}$$ (6b)

$$f_{Q,tert}=0.057\times \ln\left(T\right)-0.0083\text{for}\;\text{tertiary}\;\text{H}\;\text{abstraction}\;\text{sites}$$ (6c)

As indicated by Figure 7, the higher the order of active sites the lower the *f*_*Q*_ value. This is in accordance with expectations based on the previous reports. Stronger coupling with substituents affects (lowers) the partition function, also lowering the coefficient *f*_*Q*_.

#### 3.2.5 Hindered Rotations Factor *f*_*HR*_

This coefficient captures changes of contribution of the low frequency (hindered) rotations (HR) to the total partition function. Their handling within the harmonic oscillator (HO) approximation may lead to severe errors, thus they have to be treated separately. The influence of the anharmonic effect on the total partition function is expressed as a ratio of Q obtained with and without special treatment of these (see Supplementary Table S1 in the SI file or Baradyn and Ratkiewicz (2020)). As it is a ratio of two different HR/HO ratios, the factor *f*_*HR*_ captures the modification of the total partition function caused by accounting for anharmonicity. For the reference reaction R_1_, the hindered rotations were already treated explicitly in its reaction rate, meaning the *f*_*HR*_ could be regarded as an estimation related to the hindered rotation substituents’ effects on the total partition function. As mentioned previously, the rotations of -CH_3_ groups, as well as H rotation along the O-O bond were explicitly treated as hindered rotations. The geometry (reduced moment of inertia of rotating group) and energetic parameters needed were obtained from the electronic structure calculations at the M06-2X/aug-cc-pVTZ level of theory. As for the factors discussed above, this also depends on the active site, thus separate expressions were derived for each one of these:

$$f_{Q,pri}=-2\times 10^{-7}\times T^{2}+0.0005\times T+0.818\;\text{for}\;\text{primary}\;\text{H}\;\text{abstraction}\;\text{sites}$$ (7a)

$$f_{HR,sec}=7\times 10^{-5}\times T+1.513\text{for}\;\text{secondary}\;\text{H}\;\text{abstraction}\;\text{sites}$$ (7b)

$$f_{Q,tert}=-6\times 10^{-8}\times T^{2}+0.0003\times T+1.123\;\text{for}\;\text{tertiary}\;\text{H}\;\text{abstraction}\;\text{sites}$$ (7c)

**FIGURE 8:**
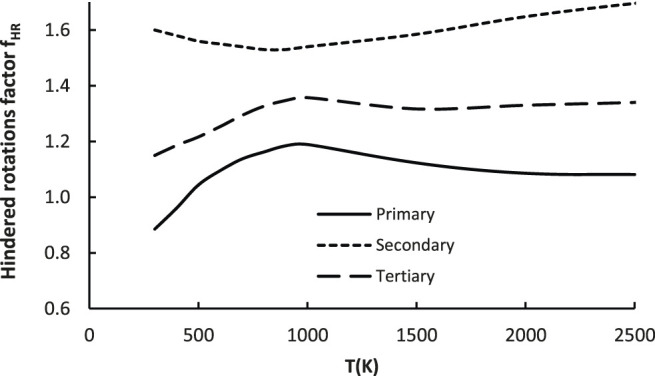
Plot of the hindered rotations factor (*f*_*HR*_) as a function of temperature for all 23 reactions considered in the representative set (R_2_–R_24_).

The above equations are plotted on Figure 8.

### 3.3 Rate Constants Prediction

Having established the RC-TST parameters (factors), we can now employ them to predict rate constants for arbitrary family representatives. The procedure is to: 1) evaluate particular factors, using formulas S(1–7) (see Supporting Info); 2) calculate the total factor as a product of the five partial factors (see Eq 2); and 3) calculate rate constants by multiplication of the total factor and rate of the reference reaction (Eq 3). The overall route is briefly summarized in Table 5. The rules presented in this table enable one to compute the rate constants of any title family member, except for (as discussed before) the principal reaction CH_4_ + ·OOH → ∙CH_3_ + H_2_O_2_, for which the rate reported by Aguilera-Iparraguirre et al. is recommended (Aguilera-Iparraguirre et al., 2008):

kprincipal(T)=1.88×10−23×T3.74×exp(−10572.53T) (cm−3s−1molecule−1) (8)

**TABLE 5:** Parameters and formulations of the RC-TST method for the ·OOH + alkane → alkyl + H_2_O_2_ reaction class (C_2_H_6_ + ·OOH → ∙C_2_H_5_ + H_2_O_2_ is the reference reaction), units of rate constants are (*cm* ^*−3*^s^*−1*^ *molecule* ^−*1*^).

$k_{a}\left(T\right)=k_{p}\left(T\right)\cdot f_{k}\left(T\right)\cdot f_{Q}\left(T\right)\cdot f_{HR}\left(T\right)\cdot f_{v}\left(T\right)\cdot f_{\sigma }f_{\nu }\left(T\right)=\exp\left[\frac{-\left(\Delta V^{\neq }-\Delta V_{r}^{\neq }\right)}{k_{B}T}\right]$	
T is in Kelvin; $\Delta V^{\neq }$ and ΔE are in kcal/mol; Zero-point energy correction is not included	
$f_{\sigma }$	Calculated explicitly from the symmetry of reactions (see Table 4)
$f_{\kappa }\left(T\right)$	fκ,pri=0.99−1.12×exp(−T169.7) for primary carbon sites
	fκ,sec=0.99−0.94×exp(−T207.57) for secondary carbon sites
	fκ,tert=0.98−0.98×exp(−T232.4) for tertiary carbon sites
$f_{Q}\left(T\right)$	$f_{Q,pri}=0.56$ for primary carbon sites
	$f_{Q,sec}=0.46$ for secondary carbon sites
	$f_{Q,tert}=0.057\times \ln\left(T\right)-0.0083$ for tertiary carbon sites
*f*_*HR*_ *(T*)	$\;f_{HR,pri}=-2\times 10^{-7}\times T^{2}+0.0005\times T+0.818$ for primary carbon sites
	$f_{HR,sec}=7\times 10^{-5}\times +1.513$ for secondary carbon sites
	$f_{HR,tert}=-6\times 10^{-8}\times T^{2}+0.0003\times T+1.123$ for tertiary carbon sites
$\Delta V^{\neq }$	$\Delta V_{pri}^{†}=-0.0732\times \Delta E+22.095$ for primary carbon sites
	$\text{Δ}V_{sec}^{†}=-1.266\times \text{Δ}E+34.805$ for secondary carbon sites
	$\text{Δ}V_{tert}^{†}=-1.937\times \text{Δ}E+37$ for tertiary carbon sites
	$\Delta V_{r}^{\neq }$ = 20.92 kcal/mol
$k_{ref}\left(T\right)$	$k_{CVT/SCT\;}\left(T\right)=5.22\times 10^{-25}\times T^{4.13}\times exp\left(\frac{-7206.9}{T}\right)$ (cm^3^ s^−1^molecule^−1^)
BHG approach (per site)	kBHG,prim(T)=1.85×10−25×T4.00×exp(−7353.8T) (cm^3^ s^−1^molecule^−1^)
	kBHG, sec(T)=4.14×10−30×T5.52×exp(−5669.1T) (cm^3^ s^−1^molecule^−1^)
	kBHG,tert(T)=3.38×10−27×T4.39×exp(−4759.7T) (cm^3^ s^−1^molecule^−1^)

The rate constants obtained with the BHG approximation are denoted by RC-TST/BHG. They can be estimated without any further calculations as (per single reaction site):

kBHG,prim(T)=1.85×10−25×T4.00×exp(−7353.8T) (cm−3s−1molecule−1) (9a)

kBHG,sec(T)=4.14×10−30×T5.52×exp(−5669.1T) (cm−3s−1molecule−1) (9b)

kBHG,tert(T)=3.38×10−27×T4.39×exp(−4759.7T) (cm−3s−1molecule−1) (9c)

To obtain the total rate constants, the above equations need to be multiplied by the appropriate symmetry number (see Table 3). Consequently, it may be concluded that only molecular topology data are needed to calculate the RC-TST/BHG rate constants.

### 3.4 Error Assessment

This section evaluates the accuracy of the proposed approach and three error analyses are reported. The first one involved a direct comparison of our results with literature data. Second, the systematic errors associated with processes from the representative set (Table 1) are evaluated. The final analysis concerns component errors.

#### 3.4.1 Comparison With Literature Data

**FIGURE 9:**
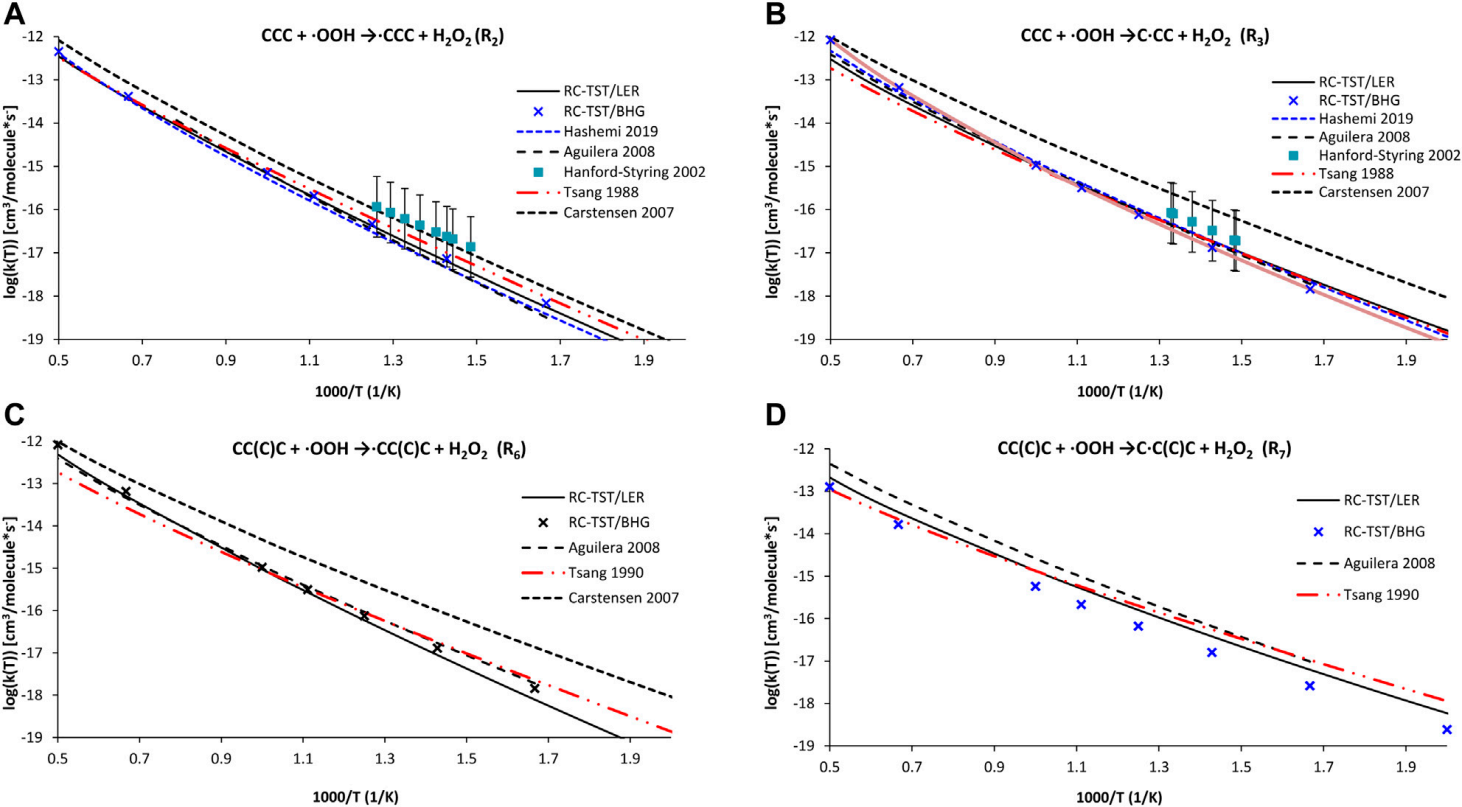
Arrhenius plots of the computed and literature rate constants calculated with the RC-TST/LER and RC-TST/BHG approaches for the: **(A)** CH_3_CH_2_CH_3_ + ·OOH →∙CH_2_CH_2_CH_3_ + H_2_O_2_ (reaction R_2_); **(B)** CH_3_CH_2_CH_3_ + OOH →CH_3_∙CHCH_3_ + H_2_O_2,_; **(C)** CH_3_CH(CH_3_)CH_3_ + ·OOH → ∙CH_2_CH(CH_3_)CH_3_ + H_2_O_2_; and **(D)** CH_3_CH(CH_3_)CH3 + ·OOH → CH_3_∙C(CH_3_)CH_3_ + H_2_O_2_ reactions. Electronic structure data were obtained at the M06-2X/aug-cc-pVTZ level.

In the first error analysis, the calculated RC-TST rates are compared with those already reported. As mentioned in the Introduction, only the indirect measurements reported by Walker and collaborators are available (Handford-Styring and Walker, 2002). The lack of measurements is partially compensated by computations (see Introduction for details) as well as extensive literature reviews (Tsang, 1988, 1990). Unfortunately, the uncertainty of these evaluations is significant. The RC-TST correlations from Table 5 and Eqs 9a–c are verified against literature reports dealing with H abstractions from *p*, *s,* and *t* active sites in propane (reactions R_2_ and R_2_) and isobutane (R_6_ and R_7_). Figures 9A–D shows the RC-TST rate of reactions R_2_ (9a), R_3_ (9b), R_6_ (9c), and R_7_ (9d) along with the appropriate literature data, taken both from original papers as well as the NIST Kinetics database (Manion et al., 2015). In this figure, the “RC-TST LER” notation indicates that the particular factors were obtained using the RC-TST correlations from Table 5. For the sake of comparison, the RC-TST/BHG rates (Eqs 12a–c are also visualized in Figure 9. It can be seen that, generally, both RC-TST/LER and RC-TST/BHG outputs are in reasonable agreement with the literature. The very good agreement between our data and those from Aguilera-Iparraguirre et al. (2008) and Hashemi et al. (2019) is noteworthy. The only exception here were reactions at the tertiary site, for which our rates were noticeably slower. It is important to keep in mind that significantly less computational effort (DFT vs high level *ab initio*) was needed and, which is even more important, as no TS calculations are now required. This facilitates RC-TST as an effective tool to be utilized in the automated mechanism generation at a reasonable cost. The noticeable difference is observed only for rates of Carstensen et al. (Carstensen and Dean, 2007). This observation holds for all computations, thus their rates seem to be systematically overestimated. However, all computational and literature results are within error bars claimed by the experiment.

**FIGURE 10:**
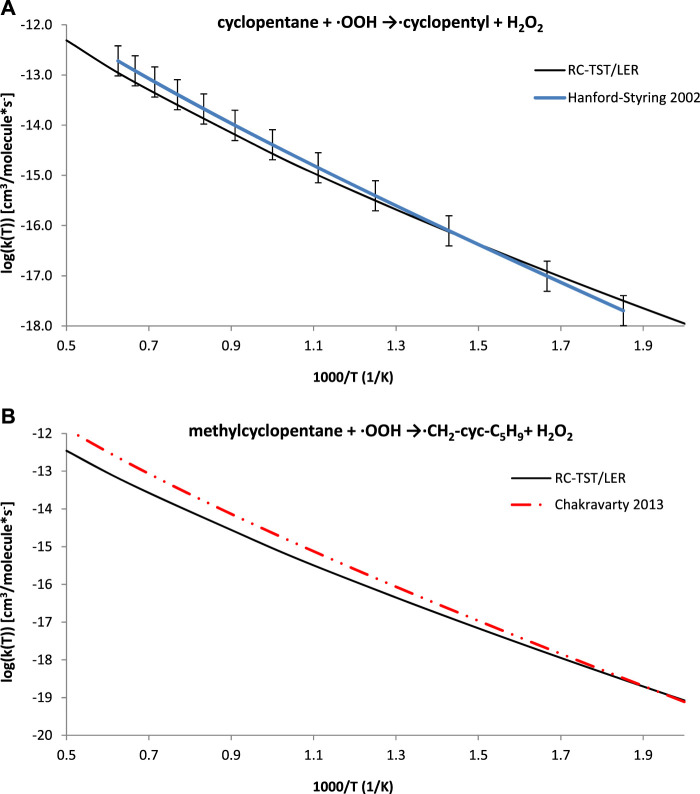
Arrhenius plots of the computed and literature rate constants calculated with the RC-TST/LER approach for the: **(A)** cyclopentane + ·OOH → cyclopentyl + H_2_O_2_ reaction; **(B)** methylcyclopentane + ·OOH →·CH_2_-cyc-C_5_H_9_+ H_2_O_2_. Electronic structure data were obtained at the M06–2X/aug-cc-pVTZ level.

Based on previous studies (Muszyńska et al., 2009; Ratkiewicz et al., 2011), the present study anticipated that the application of the formulas in Table 5 may also be similarly effective to the processes but that results would not exactly belong to the title class, i.e. H abstractions from cycloalkanes. Experimental data are available for the cyclopentane + ·OOH → cyclopentyl + H_2_O_2_ reaction (Handford-Styring and Walker, 2002), and high level TST calculations were reported for methylcyclopentane + HO_2_ → ·CH_2_-cyc-C_5_H_9_ + H_2_O_2_ (Chakravarty and Fernandes, 2013). These results, along with the RC-TST rates for both systems, are pictured in Figure 10. Even though the RC-TST rates fit within experimental error, there is a noticeable discrepancy between the high temperature rates for methylcyclopentane. It is interesting to note that the agreement is surprisingly good for T < 700K. Unfortunately, the BHG methodology was not applicable here. Despite this, it may still be concluded that RC-TST results compare well with wider literature, as the agreement is satisfactory for similar reactions but do not strictly belong the title family.

#### 3.4.2 Comparisons to Explicit Calculations

As mentioned, the correlations presented in Table 5 result from averaging computational data for the representative set and fitting results to simple analytical expressions. Errors resulting from this procedure provide valuable information about the overall performance of the RC-TST approach. A systematic analysis was performed for 23 reactions from the representative training set, results are plotted in Supplementary Figure S4A (RC-TST/LER) and S4(b) (RC-TST/BHG) in the Supporting Information of this article. The relative deviation, obtained as (|k_RC-TST_ - k_RC-TST_/_LER or BHG_|/k_RC-TST_) as a percent of a total factor (tantamount to RC-TST/Eckart rate) vs temperature was drawn. The most important parameter resulting from these plots was the error range, i.e. the y-range of the combined plots rather than tracking one particular reaction (curve). The advantage of the LER approach over the BHG is noticeable. Only four among the 24 processes show a systematic error larger than 80%. For the rest of the reactions, this value does not exceed 50%. Things are getting worse for BHG where the majority of curves exhibit an error exceeding 50% (at least) for some temperature points. Taking this into account, it may be stated that the LER approach estimates thermal rate constants within 50% compared to explicit calculations. The systematic error associated with the BHG approach is substantially higher and demonstrates larger deviations. Furthermore, as shown in the previous section, the BHG approach is not suitable to use in such cases.

#### 3.4.3 Analysis of Error Components

**FIGURE 11:**
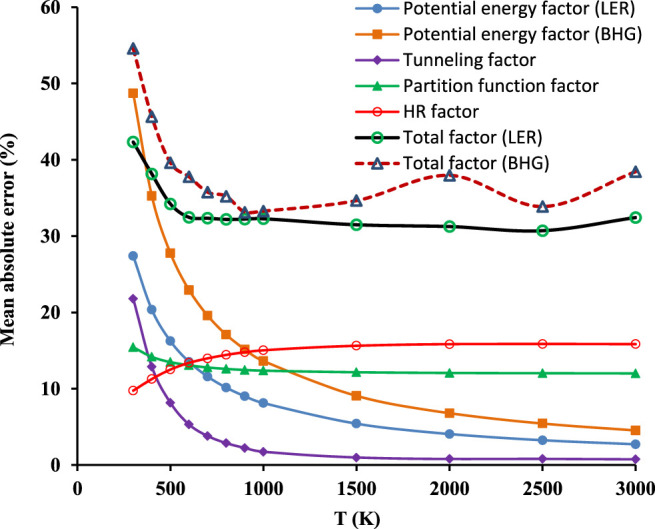
Temperature dependence of systematic errors of the RC-TST factors, namely total factors *f(T)* and its constituents: potential energy *f*_*V*_(*T*), partition function *f*_*Q*_(*T*), and hindered rotation *f*_*HR*_(*T*).

The last examination focuses on the systematic error of particular components (RC-TST factors), resulting from both averaging particular factors and fitting them to simple correlations. The deviations between the estimated and exact (i.e. obtained directly from electronic structure calculations results) RC-TST coefficients (factors) are calculated at each temperature for every reaction in the representative training set (Table 1) and averaged over the whole family. The mutual multiplication/cancelation of errors associated with particular factors may affect the results. Consequently, the total factors may not follow the trends observed for its constituents. Results are plotted in Figure 11. For T > 500K the tunneling factor introduces the smallest error. However, its contribution for T < 500K is significant, even exceeding 10%, which is not usual for this component and warrant future investigations of the nature of the quantum tunneling in this class.

Of the factors, the partition function and hindered rotations possess the slightest T dependence. The errors introduced by the others are most significant for T < 500K. It is interesting to observe that although the difference between both potential energy factors (LER and BHG) is well defined and systematically decreases with temperature, is not reflected in the relationship of the corresponding total factors. The mutual relations of these are far more complicated than the relationship of potential energy components. These results were calculated by averaging the components after their multiplication. Even though the component factor may show a larger error than the total, it is not the case in this instance, since the maximum error of the total factor (LER approach) does not exceed 45%. It may thus be safely concluded, that the averaged systematic error of the method is less than 50%.

## 4 Conclusion

This paper reports on an RC-TST study of the abstraction of H-atoms from sp^3^-hybridized carbons in alkanes by the hydroperoxyl radical OOH, forming alkyl radical and hydrogen peroxide. The rate constants of the reference reaction were obtained with the CVT/SCT method. All the parameters were derived from the DFT calculations for a training set of 24 class representatives, thus no transition state calculations are necessary. The systematic error of the method was found to be within a factor of two when compared to the explicit rate calculations. Satisfactory agreement with literature data proves that the RC-TST approach results in a nearly equally reliable rate constant at a fraction of the cost needed for larger and higher level calculations. This suggests its possible application in automated mechanism generation software.

## 5 Associated Content

Details of the implementation of the RC-TST methodology. Potential energy and bonds lengths along the C-H bond length and C-O bond length of the reaction R_1_. Definitions of the primary (*p*), secondary (*s*), and tertiary (*t*) H abstractions. Tables of optimized geometries and frequencies of all species calculated at the M06-2X/aug-cc-pVTZ level of theory for the representative set. Error analysis–comparison of the results computed directly from formulas S2−S7 with approximations: RC-TS/LER and RC-TST/BHG.

## Data Availability

The original contributions presented in the study are included in the article/Supplementary Material, further inquiries can be directed to the corresponding author.

## References

[B1] Aguilera-Iparraguirre J. Curran H. J. Klopper W. Simmie J. M. (2008). Accurate Benchmark Calculation of the Reaction Barrier Height for Hydrogen Abstraction by the Hydroperoxyl Radical from Methane. Implications for CnH2n+2 where N = 2 → 4. J. Phys. Chem. A. 112 (30), 7047–7054. 10.1021/jp8012464 18610940

[B2] Alecu I. M. Truhlar D. G. (2011a). Computational Study of the Reactions of Methanol with the Hydroperoxyl and Methyl Radicals. 1. Accurate Thermochemistry and Barrier Heights. J. Phys. Chem. A. 115 (13), 2811–2829. 10.1021/jp110024e 21405059

[B3] Alecu I. M. Truhlar D. G. (2011b). Computational Study of the Reactions of Methanol with the Hydroperoxyl and Methyl Radicals. 2. Accurate thermal Rate Constants. J. Phys. Chem. A. 115 (51), 14599–14611. 10.1021/jp209029p 22059377

[B4] Awan I. A. Burgess D. R. Manion J. A. (2012). Pressure Dependence and Branching Ratios in the Decomposition of 1-Pentyl Radicals: Shock Tube Experiments and Master Equation Modeling. J. Phys. Chem. A. 116 (11), 2895–2910. 10.1021/jp2115302 22356429

[B5] Baldwin R. R. Jones P. N. Walker R. W. (1988). Determination of the Rate Constant for HO2+ CH4→ H2O2+ CH3at 443 °C. J. Chem. Soc. Faraday Trans. 2 84 (2), 199–207. 10.1039/F29888400199

[B6] Bao J. L. Truhlar D. G. (2017). Variational Transition State Theory: Theoretical Framework and Recent Developments. Chem. Soc. Rev. 46 (24), 7548–7596. 10.1039/C7CS00602K 29165460

[B7] Baradyn M. Ratkiewicz A. (2020). Kinetics of the Hydrogen Abstraction Alkane + O2 → Alkyl + HO2 Reaction Class: an Application of the Reaction Class Transition State Theory. Struct. Chem. 31 (2), 731–746. 10.1007/s11224-019-01459-x

[B8] Battin-Leclerc F. Curran H. Faravelli T. Glaude P. A. (2013). “Specificities Related to Detailed Kinetic Models for the Combustion of Oxygenated Fuels Components,” Chapter 4 in Cleaner Combustion: Developing Detailed Chemical Kinetic Models. Editors Battin-Leclerc F. Simmie J. M. Blurock E. (London: Springer-Verlag), 93–109. 10.1007/978-1-4471-5307-8_4

[B9] Baulch D. L. Cobos C. J. Cox R. A. Esser C. Frank P. Just T. (1992). Evaluated Kinetic Data for Combustion Modelling. J. Phys. Chem. Reference Data 21 (3), 411–734. 10.1063/1.555908

[B10] Blocquet M. Schoemaecker C. Amedro D. Herbinet O. Battin-Leclerc F. Fittschen C. (2013). Quantification of OH and HO2 Radicals during the Low-Temperature Oxidation of Hydrocarbons by Fluorescence Assay by Gas Expansion Technique. Proc. Natl. Acad. Sci. 110 (50), 20014–20017. 10.1073/pnas.1314968110 24277836 PMC3864279

[B11] Carstensen H.-H. Dean A. M. Deutschmann O. (2007). Rate Constants for the H Abstraction from Alkanes (R-H) by R′O2 Radicals: A Systematic Study on the Impact of R and R′. Proc. Combustion Inst. 31 (1), 149–157. 10.1016/j.proci.2006.08.091

[B12] Carstensen H.-H. Dean A. M. (2009). Rate Constant Rules for the Automated Generation of Gas-phase Reaction Mechanisms. J. Phys. Chem. A. 113 (2), 367–380. 10.1021/jp804939v 19090679

[B13] Carstensen H.-H. Dean A. M. (2005). Rate Constants for the Abstraction Reactions RO2+C2H6; R=H, CH3, and C2H5. Proc. Combustion Inst. 30 (1), 995–1003. 10.1016/j.proci.2004.08.076

[B14] Chakravarty H. K. Fernandes R. X. (2013). Reaction Kinetics of Hydrogen Abstraction Reactions by Hydroperoxyl Radical from 2-Methyltetrahydrofuran and 2,5-Dimethyltetrahydrofuran. J. Phys. Chem. A. 117 (24), 5028–5041. 10.1021/jp402801c 23713783

[B15] Chen C.-C. Bozzelli J. W. Farrell J. T. (2004). Thermochemical Properties, Pathway, and Kinetic Analysis on the Reactions of Benzene with OH: An Elementary Reaction Mechanism. J. Phys. Chem. A. 108 (21), 4632–4652. 10.1021/jp0312823

[B16] Curran H. J. (2019). Developing Detailed Chemical Kinetic Mechanisms for Fuel Combustion. Proc. Combustion Inst. 37 (1), 57–81. 10.1016/j.proci.2018.06.054

[B17] De Oliveira L. P. Hudebine D. Guillaume D. Verstraete J. J. (2016). A Review of Kinetic Modeling Methodologies for Complex Processes. Oil Gas Sci. Technol. - Rev. IFP Energies Nouvelles 71 (3), 45. 10.2516/ogst/2016011

[B18] Duncan W. T. Bell R. L. Truong T. N. (1998). TheRate: Program Forab Initio Direct Dynamics Calculations of thermal and Vibrational-State-Selected Rate Constants. J. Comput. Chem. 19 (9), 1039–1052. 10.1002/(SICI)1096-987X(19980715)19:9<1039:AID-JCC5>3.0.CO;2-R

[B19] Duong M. V. Nguyen H. T. Truong N. Le T. N.-M. Huynh L. K. (2015). Multi-Species Multi-Channel (MSMC): An Ab Initio- Based Parallel Thermodynamic and Kinetic Code for Complex Chemical Systems. Int. J. Chem. Kinet. 47(9)**,** 564–575. 10.1002/kin.20930

[B20] Edwards D. E. Zubarev D. Y. Lester W. A. Frenklach M. (2014). Pathways to Soot Oxidation: Reaction of OH with Phenanthrene Radicals. J. Phys. Chem. A. 118 (37), 8606–8613. 10.1021/jp5033178 24761798

[B21] Fernández-Ramos A. Ellingson B. A. Meana-Pañeda R. Marques J. M. C. Truhlar D. G. (2007). Symmetry Numbers and Chemical Reaction Rates. Theor. Chem. Account. 118 (4), 813–826. 10.1007/s00214-007-0328-0

[B22] Frenklach M. Liu Z. Singh R. I. Galimova G. R. Azyazov V. N. Mebel A. M. (2018). Detailed, Sterically-Resolved Modeling of Soot Oxidation: Role of O Atoms, Interplay with Particle Nanostructure, and Emergence of Inner Particle Burning. Combustion and Flame 188, 284–306. 10.1016/j.combustflame.2017.10.012

[B23] Frisch M. J. Trucks G. W. Schlegel H. B. Scuseria G. E. Robb M. A. Cheeseman J. R. (2016). Gaussian 16 Rev. C.01. Wallingford, CT: Gaussian 16.

[B24] Gao C. W. Allen J. W. Green W. H. West R. H. (2016). Reaction Mechanism Generator: Automatic Construction of Chemical Kinetic Mechanisms. Comput. Phys. Commun. 203, 212–225. 10.1016/j.cpc.2016.02.013

[B25] Gao L. G. Zheng J. Fernández-Ramos A. Truhlar D. G. Xu X. (2018). Kinetics of the Methanol Reaction with OH at Interstellar, Atmospheric, and Combustion Temperatures. J. Am. Chem. Soc. 140 (8), 2906–2918. 10.1021/jacs.7b12773 29299932

[B26] Handford-Styring S. M. Walker R. W. (2002). Rate Constants for the Reaction of HO2 Radicals with Cyclopentane and Propane between 673 and 783 K. Phys. Chem. Chem. Phys. 4 (4), 620–627. 10.1039/B108578F

[B27] Hashemi H. Christensen J. M. Harding L. B. Klippenstein S. J. Glarborg P. (2019). High-pressure Oxidation of Propane. Proc. Combustion Inst. 37 (1), 461–468. 10.1016/j.proci.2018.07.009

[B28] Hou D. You X. (2017). Reaction Kinetics of Hydrogen Abstraction from Polycyclic Aromatic Hydrocarbons by H Atoms. Phys. Chem. Chem. Phys. 19 (45), 30772–30780. 10.1039/C7CP04964A 29134219

[B29] Huynh L. K. Ratkiewicz A. Truong T. N. (2006). Kinetics of the Hydrogen Abstraction OH + Alkane → H2O + Alkyl Reaction Class: An Application of the Reaction Class Transition State Theory. J. Phys. Chem. A. 110 (2), 473–484. 10.1021/jp051280d 16405319

[B30] Jana G. Pan S. Chattaraj P. K. (2017). Binding of Small Gas Molecules by Metal-Bipyridyl Monocationic Complexes (Metal = Cu, Ag, Au) and Possible Bond Activations Therein. J. Phys. Chem. A. 121 (19), 3803–3817. 10.1021/acs.jpca.7b02520 28448147

[B31] Jana G. Pan S. Osorio E. Zhao L. Merino G. Chattaraj P. K. (2018). Cyanide-isocyanide Isomerization: Stability and Bonding in noble Gas Inserted Metal Cyanides (Metal = Cu, Ag, Au). Phys. Chem. Chem. Phys. 20 (27), 18491–18502. 10.1039/C8CP02837K 29947384

[B32] Li C. Agarwal J. Wu C.-H. Allen W. D. Schaefer H. F. (2015). Intricate Internal Rotation Surface and Fundamental Infrared Transitions of the N-Propyl Radical. J. Phys. Chem. B 119 (3), 728–735. 10.1021/jp504764t 25007004

[B33] Li Y. Yao X. Sun X. Li Z. Wang J. Li X. (2020). Automatic Construction of Transition States and On-The-Fly Accurate Kinetic Calculations for Reaction Classes in Automated Mechanism Generators. Comput. Theor. Chem. 1184, 112852. 10.1016/j.comptc.2020.112852

[B34] Magoon G. R. Green W. H. (2013). Design and Implementation of a Next-Generation Software Interface for On-The-Fly Quantum and Force Field Calculations in Automated Reaction Mechanism Generation. Comput. Chem. Eng. 52, 35–45. 10.1016/j.compchemeng.2012.11.009

[B35] Mai T. V.-T. Ratkiewicz A. Le A. Duong M. v. Truong T. N. Huynh L. K. (2018). On-the-fly Kinetics of Hydrogen Abstraction from Polycyclic Aromatic Hydrocarbons by Methyl/ethyl Radicals. Phys. Chem. Chem. Phys. 20 (36), 23578–23592. 10.1039/C8CP03718C 30188552

[B36] Manion J. A. Huie R. E. Levin R. D. Burgess D. R. Jr. Orkin V. L. Tsang W. (2015). NIST Chemical Kinetics Database, NIST Standard Reference Database 17, Version 7.0 (Web Version). Release 1.6.8, Data version 2015.12.

[B37] Muszyńska M. Ratkiewicz A. Huynh L. K. Truong T. N. (2009). Kinetics of the Hydrogen Abstraction C2H3 + Alkane → C2H4 + Alkyl Radical Reaction Class. J. Phys. Chem. A. 113 (29), 8327–8336. 10.1021/jp903762x 19569659

[B38] Pan S. Jana G. Gupta A. Merino G. Chattaraj P. K. (2017). Endohedral Gas Adsorption by Cucurbit[7]uril: a Theoretical Study. Phys. Chem. Chem. Phys. 19 (36), 24448–24452. 10.1039/C7CP03984K 28853457

[B39] Panadés-Barrueta R. L. Martínez-Núñez E. Peláez D. (2019). Specific Reaction Parameter Multigrid POTFIT (SRP-MGPF): Automatic Generation of Sum-Of-Products Form Potential Energy Surfaces for Quantum Dynamical Calculations. Front. Chem. 7 (576). 10.3389/fchem.2019.00576 PMC6702682 31475138

[B40] Pattanaik L. Ingraham J. B. Grambow C. A. Green W. H. (2020). Generating Transition States of Isomerization Reactions with Deep Learning. Phys. Chem. Chem. Phys. 22 (41), 23618–23626. 10.1039/D0CP04670A 33112304

[B41] Ratkiewicz A. Bankiewicz B. Truong T. N. (2010). Kinetics of Thermoneutral Intermolecular Hydrogen Migration in Alkyl Radicals. Phys. Chem. Chem. Phys. 12, 10988–10995. 10.1039/c0cp00293c 20664879

[B42] Ratkiewicz A. Bieniewska J. Truong T. N. (2011). Kinetics of the Hydrogen Abstraction R−OH + H → R −OH + H2 Reaction Class: An Application of the Reaction Class Transition State Theory. Int. J. Chem. Kinet. 43 (2), 78–98. 10.1002/kin.20531

[B43] Ratkiewicz A. Huynh L. K. Pham Q. B. Truong T. N. (2013). Kinetics of the Hydrogen Abstraction ·C2H5 + Alkane → C2H6 + Alkyl Reaction Class: an Application of the Reaction Class Transition State Theory. Theor. Chem. Acc. 132 (3), 1344. 10.1007/s00214-013-1344-x

[B44] Ratkiewicz A. Huynh L. K. Truong T. N. (2016). Performance of First-Principles-Based Reaction Class Transition State Theory. J. Phys. Chem. B 120 (8), 1871–1884. 10.1021/acs.jpcb.5b09564 26752508

[B45] Saeys M. Reyniers M.-F. Marin G. B. Van Speybroeck V. Waroquier M. (2003). Ab Initio Calculations for Hydrocarbons: Enthalpy of Formation, Transition State Geometry, and Activation Energy for Radical Reactions. J. Phys. Chem. A. 107 (43), 9147–9159. 10.1021/jp021706d

[B46] Saeys M. Reyniers M.-F. Van Speybroeck V. Waroquier M. Marin G. B. (2006). Ab Initio Group Contribution Method for Activation Energies of Hydrogen Abstraction Reactions. ChemPhysChem 7 (1), 188–199. 10.1002/cphc.200500206 16323223

[B47] Scott M. Walker R. W. (2002). Addition of Toluene and Ethylbenzene to Mixtures of H2 and O2 at 773 K. Combustion and Flame 129, 365–377. 10.1016/s0010-2180(02)00350-4

[B48] Semenikhin A. S. Savchenkova A. S. Chechet I. V. Matveev S. G. Liu Z. Frenklach M. (2017). Rate Constants for H Abstraction from Benzo(a)pyrene and Chrysene: a Theoretical Study. Phys. Chem. Chem. Phys. 19 (37), 25401–25413. 10.1039/C7CP05560A 28894870

[B49] Shi S. (2018). Advances in Modeling Hydrocarbon Cracking Kinetic Predictions by Quantum Chemical Theory: A Review. Int. J. Energ. Res. 42 (10), 3164–3181. 10.1002/er.4049

[B50] Sirjean B. Dames E. Wang H. Tsang W. (2012). Tunneling in Hydrogen-Transfer Isomerization of N-Alkyl Radicals. J. Phys. Chem. A. 116 (1), 319–332. 10.1021/jp209360u 22129143

[B51] Truong T. N. Duncan W. T. Tirtowidjojo M. (1999). A Reaction Class Approach for Modeling Gas Phase Reaction Rates. Phys. Chem. Chem. Phys. 1 (6), 1061–1065. 10.1039/A808438F

[B52] Truong T. N. (2000). Reaction Class Transition State Theory: Hydrogen Abstraction Reactions by Hydrogen Atoms as Test Cases. J. Chem. Phys. 113 (12), 4957–4964. 10.1063/1.1287839

[B53] Tsang W. (1990). Chemical Kinetic Data Base for Combustion Chemistry Part 4. Isobutane. J. Phys. Chem. Reference Data 19 (1), 1–68. 10.1063/1.555877

[B54] Tsang W. (1988). Chemical Kinetic Data Base for Combustion Chemistry. Part 3: Propane. J. Phys. Chem. Reference Data 17 (2), 887–951. 10.1063/1.555806

[B55] Van de Vijver R. Van Geem K. M. Marin G. B. (2019). On-the-fly Ab Initio Calculations toward Accurate Rate Coefficients. Proc. Combustion Inst. 37 (1), 283–290. 10.1016/j.proci.2018.05.056

[B56] Van de Vijver R. Vandewiele N. M. Bhoorasingh P. L. Slakman B. L. Seyedzadeh Khanshan F. Carstensen H.-H. (2015). Automatic Mechanism and Kinetic Model Generation for Gas- and Solution-phase Processes: A Perspective on Best Practices, Recent Advances, and Future Challenges. Int. J. Chem. Kinet. 47 (4), 199–231. 10.1002/kin.20902

[B57] Walker R. W. Morley C. (1997). “Chapter 1 Basic Chemistry of Combustion,” in Low-Temperature Combustion and Autoignition. Editor Pilling M. J. (Amsterdam: Elsevier), 1–124. 10.1016/s0069-8040(97)80016-7

[B58] Wang Q.-D. Sun M.-M. Liang J.-H. (2019). Reaction Mechanisms and Kinetics of the Hydrogen Abstraction Reactions of C4-C6 Alkenes with Hydroxyl Radical: A Theoretical Exploration. Int. J. Mol. Sci. 20 (6), 1275. 10.3390/ijms20061275 PMC6471405 30875716

[B59] Weininger D. (1988). SMILES, a Chemical Language and Information System. 1. Introduction to Methodology and Encoding Rules. J. Chem. Inf. Model. 28 (1), 31–36. 10.1021/ci00057a005

[B60] Zhao Y. Truhlar D. G. (2008). The M06 Suite of Density Functionals for Main Group Thermochemistry, Thermochemical Kinetics, Noncovalent Interactions, Excited States, and Transition Elements: Two New Functionals and Systematic Testing of Four M06-Class Functionals and 12 Other Functionals. Theor. Chem. Account. 120 (1), 215–241. 10.1007/s00214-007-0310-x

[B61] Zheng J. Bao J. L. Meana-Pañeda R. Zhang S. Lynch B. L. Corchado J. C. (2017). Polyrate 17-C: Computer Program for the Calculation of Chemical Reaction Rates for Polyatomics.

