# Supplementary material for: On-The-Fly Kinetics of the Hydrogen Abstraction by Hydroperoxyl Radical: An Application of the Reaction Class Transition State Theory
Source: Front Chem. 2022 Jan 31;9:806873. doi: 10.3389/fchem.2021.806873 (PMC8841336; doi:10.3389/fchem.2021.806873)
Supplement: Supplementary file 1 [file DataSheet1.pdf]

Supporting Information for

On-the-fly Kinetics of the Hydrogen Abstraction by Hydroperoxyl Radical: An Application of the Reaction Class Transition State Theory

Maciej Baradyn, Artur Ratkiewicz*

Address: Department of Chemistry, University of Bialystok, ul. Ciolkowskiego 1K, 15-245 Białystok, Poland

Email: artrat@uwb.edu.pl

* Corresponding author

Figure S1: Definitions of the primary (*p*), secondary (*s*) and tertiary (*t*) H abstractions. 8
Figure S2: Definitions of the RC-TST factors...8
Figure S3(a-b): Potential energy and bonds lengths along the (a) C-H bond length and (b) C-O bond length of the reaction R1 (C_2H_6 + ·OOH → ·C_2H_5 + H_2O_2). V_C is the classical adiabatic ground-state potential, whereas Vga symbolizes the vibrationally adiabatic ground-state potential energy curve. H atom is transferred from C to O, see Figure 2 in the main article. 10
Table S1: Geometry (Å) of OOH molecule, calculated at the M06-2X/aug-cc-pVTZ level of theory..12
Table S2: Frequencies (cm^{-1}) of OOH molecule, calculated at the M06-2X/aug-cc-pVTZ level of theory. 12
Table S3: Geometry (Å) of H_2O_2 molecule, calculated at the M06-2X/aug-cc-pVTZ level of theory..12
Table S4: Frequencies (cm^{-1}) of H_2O_2 molecule, calculated at the M06-2X/aug-cc-pVTZ level of theory. 12
Table S5: Geometry (Å) of ethane molecule, calculated at the M06-2X/aug-cc-pVTZ level of theory..12
Table S6: Frequencies (cm^{-1}) of ethane molecule, calculated at the M06-2X/aug-cc-pVTZ level of theory...13
Table S7: Geometry (Å) of ethane + OOH transition state, calculated at the M06-2X/aug-cc-pVTZ level of theory...13
Table S7: Frequencies (cm^{-1}) of ethane + OOH transition state, calculated at the M06-2X/aug-cc-pVTZ level of theory...13
Table S8: Geometry (Å) of ethyl molecule, calculated at the M06-2X/aug-cc-pVTZ level of theory..13
Table S9: Frequencies (cm^{-1}) of ethyl molecule, calculated at the M06-2X/aug-cc-pVTZ level of theory. 14
Table S10: Geometry (Å) of propane molecule, calculated at the M06-2X/aug-cc-pVTZ level of theory. 14
Table S11: Frequencies (cm^{-1}) of propane molecule, calculated at the M06-2X/aug-cc-pVTZ level of theory...14
Table S12: Geometry (Å) of propane + OOH → 1-propyl + H_2O_2 transition state, calculated at the M06-2X/aug-cc-pVTZ level of theory. 14
Table S13: Frequencies (cm^{-1}) of propane + OOH → 1-propyl + H_2O_2 transition state, calculated at the M06-2X/aug-cc-pVTZ level of theory. 15
Table S14: Geometry (Å) of 1-propyl radical, calculated at the M06-2X/aug-cc-pVTZ level of theory. 15
Table S15: Frequencies (cm^{-1}) of 1-propyl radical, calculated at the M06-2X/aug-cc-pVTZ level of theory...16
Table S16: Geometry (Å) of propane + OOH → 2-propyl + H_2O_2 transition state, calculated at the M06-2X/aug-cc-pVTZ level of theory. 16
Table S17: Frequencies (cm^{-1}) of propane + OOH → 2-propyl + H_2O_2 transition state, calculated at the M06-2X/aug-cc-pVTZ level of theory. 16

Table S18: Geometry (Å) of 2-propyl radical, calculated at the M06-2X/aug-cc-pVTZ level of theory. 16
Table S19: Frequencies (cm^{-1}) of 1-propyl radical, calculated at the M06-2X/aug-cc-pVTZ level of theory....17
Table S20: Geometry (Å) of butane molecule, calculated at the M06-2X/aug-cc-pVTZ level of theory. 17
Table S21: Frequencies (cm^{-1}) of butane molecule, calculated at the M06-2X/aug-cc-pVTZ level of theory....18
Table S22: Geometry (Å) of butane + OOH → 1-butyl + H_2O_2 transition state, calculated at the M06-2X/aug-cc-pVTZ level of theory. 18
Table S23: Frequencies (cm^{-1}) of butane + OOH → 1-butyl + H_2O_2 transition state, calculated at the M06-2X/aug-cc-pVTZ level of theory. 18
Table S24: Geometry (Å) of 1-butyl radical, calculated at the M06-2X/aug-cc-pVTZ level of theory....19
Table S25: Frequencies (cm^{-1}) of 1-butyl radical, calculated at the M06-2X/aug-cc-pVTZ level of theory....19
Table S26: Geometry (Å) of butane + OOH → 2-butyl + H_2O_2 transition state, calculated at the M06-2X/aug-cc-pVTZ level of theory. 19
Table S27: Frequencies (cm^{-1}) of butane + OOH → 2-butyl + H_2O_2 transition state, calculated at the M06-2X/aug-cc-pVTZ level of theory. 20
Table S28: Geometry (Å) of 2-butyl radical, calculated at the M06-2X/aug-cc-pVTZ level of theory....20
Table S29: Frequencies (cm^{-1}) of 2-butyl radical, calculated at the M06-2X/aug-cc-pVTZ level of theory....21
Table S30: Geometry (Å) of isobutane molecule, calculated at the M06-2X/aug-cc-pVTZ level of theory....21
Table S31: Frequencies (cm^{-1}) of isobutane molecule, calculated at the M06-2X/aug-cc-pVTZ level of theory....21
Table S32: Geometry (Å) of isobutane + OOH → 1-isobutyl + H_2O_2 transition state, calculated at the M06-2X/aug-cc-pVTZ level of theory. 22
Table S33: Frequencies (cm^{-1}) of isobutane + OOH → 1-isobutyl + H_2O_2 transition state, calculated at the M06-2X/aug-cc-pVTZ level of theory. 22
Table S34: Geometry (Å) of 1-isobutyl radical, calculated at the M06-2X/aug-cc-pVTZ level of theory. 22
Table S35: Frequencies (cm^{-1}) of 1-isobutyl radical, calculated at the M06-2X/aug-cc-pVTZ level of theory....23
Table S36: Geometry (Å) of isobutane + OOH → 2-isobutyl + H_2O_2 transition state, calculated at the M06-2X/aug-cc-pVTZ level of theory. 23
Table S37: Frequencies (cm^{-1}) of isobutane + OOH → 2-isobutyl + H_2O_2 transition state, calculated at the M06-2X/aug-cc-pVTZ level of theory. 24
Table S38: Geometry (Å) of 2-isobutyl radical, calculated at the M06-2X/aug-cc-pVTZ level of theory. 24

Table S39: Frequencies (cm^{-1}) of 2-isobutyl radical, calculated at the M06-2X/aug-cc-pVTZ level of theory....24
Table S40: Geometry (Å) of pentane molecule, calculated at the M06-2X/aug-cc-pVTZ level of theory. 25
Table S41: Frequencies (cm^{-1}) of pentane molecule, calculated at the M06-2X/aug-cc-pVTZ level of theory....25
Table S42: Geometry (Å) of pentane + OOH → 1-pentyl + H_2O_2 transition state, calculated at the M06-2X/aug-cc-pVTZ level of theory. 25
Table S43: Frequencies (cm^{-1}) of pentane + OOH → 1-pentyl + H_2O_2 transition state, calculated at the M06-2X/aug-cc-pVTZ level of theory. 26
Table S44: Geometry (Å) of 1-pentyl radical, calculated at the M06-2X/aug-cc-pVTZ level of theory....26
Table S45: Frequencies (cm^{-1}) of 1-pentyl radical, calculated at the M06-2X/aug-cc-pVTZ level of theory....27
Table S46: Geometry (Å) of pentane + OOH →2-pentyl + H_2O_2 transition state, calculated at the M06-2X/aug-cc-pVTZ level of theory. 27
Table S47: Frequencies (cm^{-1}) of pentane + OOH → 2-pentyl + H_2O_2 transition state, calculated at the M06-2X/aug-cc-pVTZ level of theory. 28
Table S48: Geometry (Å) of 2-pentyl radical, calculated at the M06-2X/aug-cc-pVTZ level of theory....28
Table S49: Frequencies (cm^{-1}) of 2-pentyl radical, calculated at the M06-2X/aug-cc-pVTZ level of theory....28
Table S50: Geometry (Å) of pentane + OOH →3-pentyl + H_2O_2 transition state, calculated at the M06-2X/aug-cc-pVTZ level of theory. 29
Table S51: Frequencies (cm^{-1}) of pentane + OOH → 3-pentyl + H_2O_2 transition state, calculated at the M06-2X/aug-cc-pVTZ level of theory. 29
Table S52: Geometry (Å) of 3-pentyl radical, calculated at the M06-2X/aug-cc-pVTZ level of theory....30
Table S53: Frequencies (cm^{-1}) of 3-pentyl radical, calculated at the M06-2X/aug-cc-pVTZ level of theory....30
Table S54: Geometry (Å) of isopentane molecule, calculated at the M06-2X/aug-cc-pVTZ level of theory....30
Table S55: Frequencies (cm^{-1}) of isopentane molecule, calculated at the M06-2X/aug-cc-pVTZ level of theory. 31
Table S56: Geometry (Å) of isopentane + OOH → 2-methyl-but-1-yl + H_2O_2 transition state (R11), calculated at the M06-2X/aug-cc-pVTZ level of theory....31
Table S57: Frequencies (cm^{-1}) of isopentane + OOH → 2-methyl-but-1-yl + H_2O_2 transition state (R11), calculated at the M06-2X/aug-cc-pVTZ level of theory. 32
Table S58: Geometry (Å) of 2-methyl-but-1-yl radical, calculated at the M06-2X/aug-cc-pVTZ level of theory. 32
Table S59: Frequencies (cm^{-1}) of 2-methyl-but-1-yl radical, calculated at the M06-2X/aug-cc-pVTZ level of theory. 32

Table S60: Geometry (Å) of isopentane + OOH → 2-methyl-but-2-yl + H_2O_2 (R12) transition state, calculated at the M06-2X/aug-cc-pVTZ level of theory....33
Table S61: Frequencies (cm^{-1}) of isopentane + OOH → 2-methyl-but-2-yl + H_2O_2 (R12) transition state, calculated at the M06-2X/aug-cc-pVTZ level of theory....33
Table S62: Geometry (Å) of 2-methyl-but-2-yl radical, calculated at the M06-2X/aug-cc-pVTZ level of theory....34
Table S63: Frequencies (cm^{-1}) of 2-methyl-but-2-yl radical, calculated at the M06-2X/aug-cc-pVTZ level of theory....34
Table S64: Geometry (Å) of isopentane + OOH → 3-methyl-but-2-yl + H_2O_2 (R13) transition state, calculated at the M06-2X/aug-cc-pVTZ level of theory....34
Table S65: Frequencies (cm^{-1}) of isopentane + OOH → 3-methyl-but-2-yl + H_2O_2 (R13) transition state, calculated at the M06-2X/aug-cc-pVTZ level of theory....35
Table S66: Geometry (Å) of 3-methyl-but-2-yl radical, calculated at the M06-2X/aug-cc-pVTZ level of theory....35
Table S67: Frequencies (cm^{-1}) of 3-methyl-but-2-yl radical, calculated at the M06-2X/aug-cc-pVTZ level of theory....36
Table S68: Geometry (Å) of isopentane + OOH → 3-methyl-but-1-yl + H_2O_2 (R14) transition state, calculated at the M06-2X/aug-cc-pVTZ level of theory....36
Table S69: Frequencies (cm^{-1}) of isopentane + OOH → 3-methyl-but-1-yl + H_2O_2 (R14) transition state, calculated at the M06-2X/aug-cc-pVTZ level of theory....37
Table S70: Geometry (Å) of 3-methyl-but-1-yl radical, calculated at the M06-2X/aug-cc-pVTZ level of theory....37
Table S71: Frequencies (cm^{-1}) of 3-methyl-but-1-yl radical, calculated at the M06-2X/aug-cc-pVTZ level of theory....37
Table S72: Geometry (Å) of 2,2-dimethylpropane molecule, calculated at the M06-2X/aug-cc-pVTZ level of theory....38
Table S73: Frequencies (cm^{-1}) of 2,2-dimethylpropane molecule, calculated at the M06-2X/aug-cc-pVTZ level of theory....38
Table S74: Geometry (Å) of 2,2-dimethylpropane + OOH → 2,2-dimethylpropyl + H_2O_2 transition state (R15), calculated at the M06-2X/aug-cc-pVTZ level of theory....38
Table S75: Frequencies (cm^{-1}) of of 2,2-dimethylpropane + OOH → 2,2-dimethylpropyl + H_2O_2 transition state (R15), calculated at the M06-2X/aug-cc-pVTZ level of theory....39
Table S76: Geometry (Å) of 2,2-dimethylpropyl radical, calculated at the M06-2X/aug-cc-pVTZ level of theory....39
Table S77: Frequencies (cm^{-1}) of 2,2-dimethylpropyl radical, calculated at the M06-2X/aug-cc-pVTZ level of theory....40
Table S78: Geometry (Å) (cm^{-1}) of hexane molecule, calculated at the M06-2X/aug-cc-pVTZ level of theory....40
Table S79: Frequencies (cm^{-1}) of hexane molecule, calculated at the M06-2X/aug-cc-pVTZ level of theory....41
Table S81: Frequencies (cm^{-1}) of hexane + OOH → 1-hexyl + H_2O_2 transition state (R16), calculated at the M06-2X/aug-cc-pVTZ level of theory....42

Table S82: Geometry (Å) of 1-hexyl radical, calculated at the M06-2X/aug-cc-pVTZ level of theory....42
Table S83: Frequencies (cm^{-1}) of 1-hexyl radical, calculated at the M06-2X/aug-cc-pVTZ level of theory....42
Table S85: Frequencies (cm^{-1}) of hexane + OOH → 2-hexyl + H_2O_2 transition state (R17), calculated at the M06-2X/aug-cc-pVTZ level of theory....43
Table S86: Geometry (Å) of 2-hexyl radical, calculated at the M06-2X/aug-cc-pVTZ level of theory....44
Table S87: Frequencies (cm^{-1}) of 2-hexyl radical, calculated at the M06-2X/aug-cc-pVTZ level of theory....44
Table S89: Frequencies (cm^{-1}) of hexane + OOH → 3-hexyl + H_2O_2 transition state (R18), calculated at the M06-2X/aug-cc-pVTZ level of theory....45
Table S90: Geometry (Å) of 3-hexyl radical, calculated at the M06-2X/aug-cc-pVTZ level of theory....45
Table S91: Frequencies (cm^{-1}) of 3-hexyl radical, calculated at the M06-2X/aug-cc-pVTZ level of theory....46
Table S92: Geometry (Å) of isohexane molecule, calculated at the M06-2X/aug-cc-pVTZ level of theory....46
Table S93: Frequencies (cm^{-1}) of isohexane molecule, calculated at the M06-2X/aug-cc-pVTZ level of theory....47
Table S95: Frequencies (cm^{-1}) of isohexane + OOH → 2-isohexyl + H_2O_2 transition state (R19), calculated at the M06-2X/aug-cc-pVTZ level of theory....48
Table S96: Geometry (Å) of 2-isohexyl radical, calculated at the M06-2X/aug-cc-pVTZ level of theory....48
Table S97: Frequencies (cm^{-1}) of 2-isohexyl radical, calculated at the M06-2X/aug-cc-pVTZ level of theory....49
Table S99: Frequencies (cm^{-1}) of isohexane + OOH → 2-isohexyl + H_2O_2 transition state (R20), calculated at the M06-2X/aug-cc-pVTZ level of theory....50
Table S100: Geometry (Å) of 3-isohexyl radical, calculated at the M06-2X/aug-cc-pVTZ level of theory....50
Table S101: Frequencies (cm^{-1}) of 3-isohexyl radical, calculated at the M06-2X/aug-cc-pVTZ level of theory....50
Table S102: Geometry (Å) of 3-methylpentane molecule, calculated at the M06-2X/aug-cc-pVTZ level of theory....51
Table S103: Frequencies (cm^{-1}) of 3-methylpentane molecule, calculated at the M06-2X/aug-cc-pVTZ level of theory....51
Table S105: Frequencies (cm^{-1}) of 3-methylpentane + OOH → 3-methylpent-3-yl + H_2O_2 transition state (R21), calculated at the M06-2X/aug-cc-pVTZ level of theory....52
Table S106: Geometry (Å) of 3-methylpent-3-yl radical, calculated at the M06-2X/aug-cc-pVTZ level of theory....53
Table S107: Frequencies (cm^{-1}) of 3-methylpent-3-yl, calculated at the M06-2X/aug-cc-pVTZ level of theory....53

Table S108: Geometry (Å) of heptane molecule, calculated at the M06-2X/aug-cc-pVTZ level of theory.....53
Table S109: Frequencies (cm^{-1}) of heptane molecule, calculated at the M06-2X/aug-cc-pVTZ level of theory.....54
Table S111: Frequencies (cm^{-1}) of heptane + OOH → 2-heptyl + H_2O_2 transition state (R22), calculated at the M06-2X/aug-cc-pVTZ level of theory.....55
Table S112: Geometry (Å) of 2-heptyl radical, calculated at the M06-2X/aug-cc-pVTZ level of theory.....55
Table S113: Frequencies (cm^{-1}) of 2-heptyl radical, calculated at the M06-2X/aug-cc-pVTZ level of theory.....56
Table S114: Geometry (Å) of isoheptane molecule, calculated at the M06-2X/aug-cc-pVTZ level of theory.....56
Table S115: Frequencies (cm^{-1}) of isoheptane molecule, calculated at the M06-2X/aug-cc-pVTZ level of theory.....57
Table S117: Frequencies (cm^{-1}) of isoheptane + OOH → 2-isoheptyl + H_2O_2 transition state (R23), calculated at the M06-2X/aug-cc-pVTZ level of theory.....58
Table S118: Geometry (Å) of 2-isoheptyl radical, calculated at the M06-2X/aug-cc-pVTZ level of theory.....58
Table S119: Frequencies (cm^{-1}) of 2-isoheptyl radical, calculated at the M06-2X/aug-cc-pVTZ level of theory.....59
Table S121: Frequencies (cm^{-1}) of isoheptane + OOH → 3-isoheptyl + H_2O_2 transition state (R24), calculated at the M06-2X/aug-cc-pVTZ level of theory.....60
Table S122: Geometry (Å) of 3-isoheptyl radical, calculated at the M06-2X/aug-cc-pVTZ level of theory.....60
Table S123: Frequencies (cm^{-1}) of 3-isoheptyl radical, calculated at the M06-2X/aug-cc-pVTZ level of theory.....61
Figure S4(a-b): Relative absolute deviations as functions of the temperature between rate constants calculated from explicit TST/Eckart calculations for all selected reactions and: (a) from the RC-TST/LER method where M062X reaction energies were used for the LER (b) from the RCT-TST/BHG method.....61

Figure S1: Definitions of the primary (*p*), secondary (*s*) and tertiary (*t*) H abstractions.

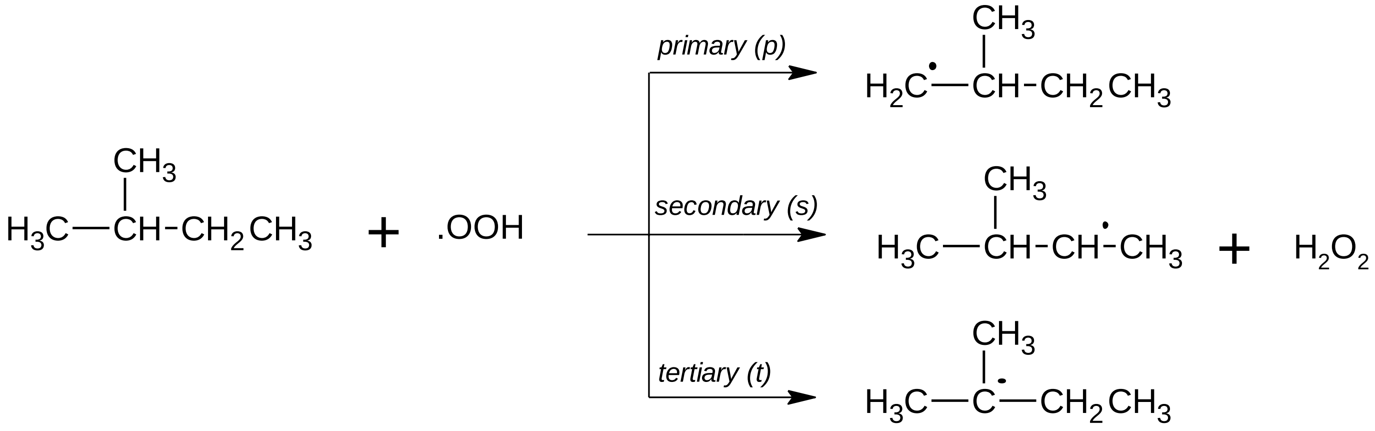

Figure S2: Definitions of the RC-TST factors.

Symmetry factor does not depend on temperature:

$$f_\sigma = \frac{\sigma}{\sigma_{ref}} \qquad \text{(S1)}$$

where σ and σ_{ref} are symmetry numbers of the arbitrary and reference reactions, respectively. As detailed in the Section 3.2.2, they are tantamount to the number of symmetry equivalent paths leading from reactant to product. The other factors are temperature dependent, the first one of them deals with quantum tunneling:

$$f_\kappa(T) = \frac{\kappa(T)}{\kappa_{ref}(T)} \qquad \text{(S2)}$$

In Eq. (4) $\kappa(T)$ is the transmission coefficient, measuring the quantum tunneling contribution to the rates constants of the arbitrary and reference reactions. The tunneling factor adjusts additional contributions due to the substituents. The next factor corresponds to the total partition functions and is defined as:

$$f_Q(T) = \frac{\left(\frac{Q_a^{\ddagger}(T)}{\Phi_a^R(T)}\right)}{\left(\frac{Q_{ref}^{\ddagger}(T)}{\Phi_{ref}^R(T)}\right)} = \left(\frac{Q_a^{\ddagger}(T)}{Q_{ref}^{\ddagger}(T)}\right) \times \left(\frac{\Phi_{ref}^R(T)}{\Phi_a^R(T)}\right) \qquad \text{(S3)}$$

All symbols in the above equation refer to the total partition functions of transition states ($Q_a^{\ddagger}(T)$ and $Q_{ref}^{\ddagger}(T)$) and reactants ($\Phi_a^R(T)$ and $\Phi_{ref}^R(T)$) of the arbitrary (subscript *a*) and reference (subscript *ref*) reactions, respectively. This factor captures the difference between total partition functions of the reference process and any other arbitrary reaction within the title family. The potential energy factor $f_V(T)$ is given by the formula:

$$f_V(T) = exp\left(-\frac{\Delta V^{\ddagger} - \Delta V_{ref}^{\ddagger}}{k_B T}\right) = exp\left(-\frac{\Delta\Delta V^{\ddagger}}{k_B T}\right) \quad \text{(S4)}$$

where $\Delta V^{\ddagger}$ and $\Delta V_{ref}^{\ddagger}$ are barriers of the arbitrary reactions and the reference one, respectively. This faction reflects the differences between reaction barriers within the title class. The last factor $f_{HR}(T)$ gauges correction to the total partition functions due to the vibrational modes that cannot be accurately treated within the harmonic approximation:

$$f_{HR}(T) = \frac{c(T)}{c_{ref}(T)} \quad \text{(S5)}$$

$$c(T) = \frac{\frac{Q_{HR}^{\ddagger}(T)}{Q_{HO}^{\ddagger}(T)}}{\frac{\Phi_{HR}(T)}{\Phi_{HO}(T)}} = \frac{Q_{HR}^{\ddagger}(T)}{Q_{HO}^{\ddagger}(T)} \times \frac{\Phi_{HO}(T)}{\Phi_{HR}(T)} \quad \text{(S6)}$$

$$c_{ref}(T) = \frac{\frac{Q_{HR,ref}^{\ddagger}(T)}{Q_{HO,ref}^{\ddagger}(T)}}{\frac{\Phi_{HR,ref}(T)}{\Phi_{HO,ref}(T)}} = \frac{Q_{HR,ref}^{\ddagger}(T)}{Q_{HO,ref}^{\ddagger}(T)} \times \frac{\Phi_{HO,ref}(T)}{\Phi_{HR,ref}(T)} \quad \text{(S7)}$$

In the formulas above, $c(T)$ and $c_{ref}(T)$ are the ratios of total partition functions part of the CTST formulas (marked green on the Fig.1), calculated with (subscript HR) and without (subscript HO) explicit hindered rotations treatment for the arbitrary ($c(T)$) and reference ($c_{ref}(T)$) reactions, respectively.

Figure S3(a-b): Potential energy and bonds lengths along the (a) C-H bond length and (b) C-O bond length of the reaction R1 (C_2H_6 + ·OOH → ·C_2H_5 + H_2O_2). V_C is the classical adiabatic ground-state potential, whereas V_g^a symbolizes the vibrationally adiabatic ground-state potential energy curve. H atom is transferred from C to O, see Figure 2 in the main article.

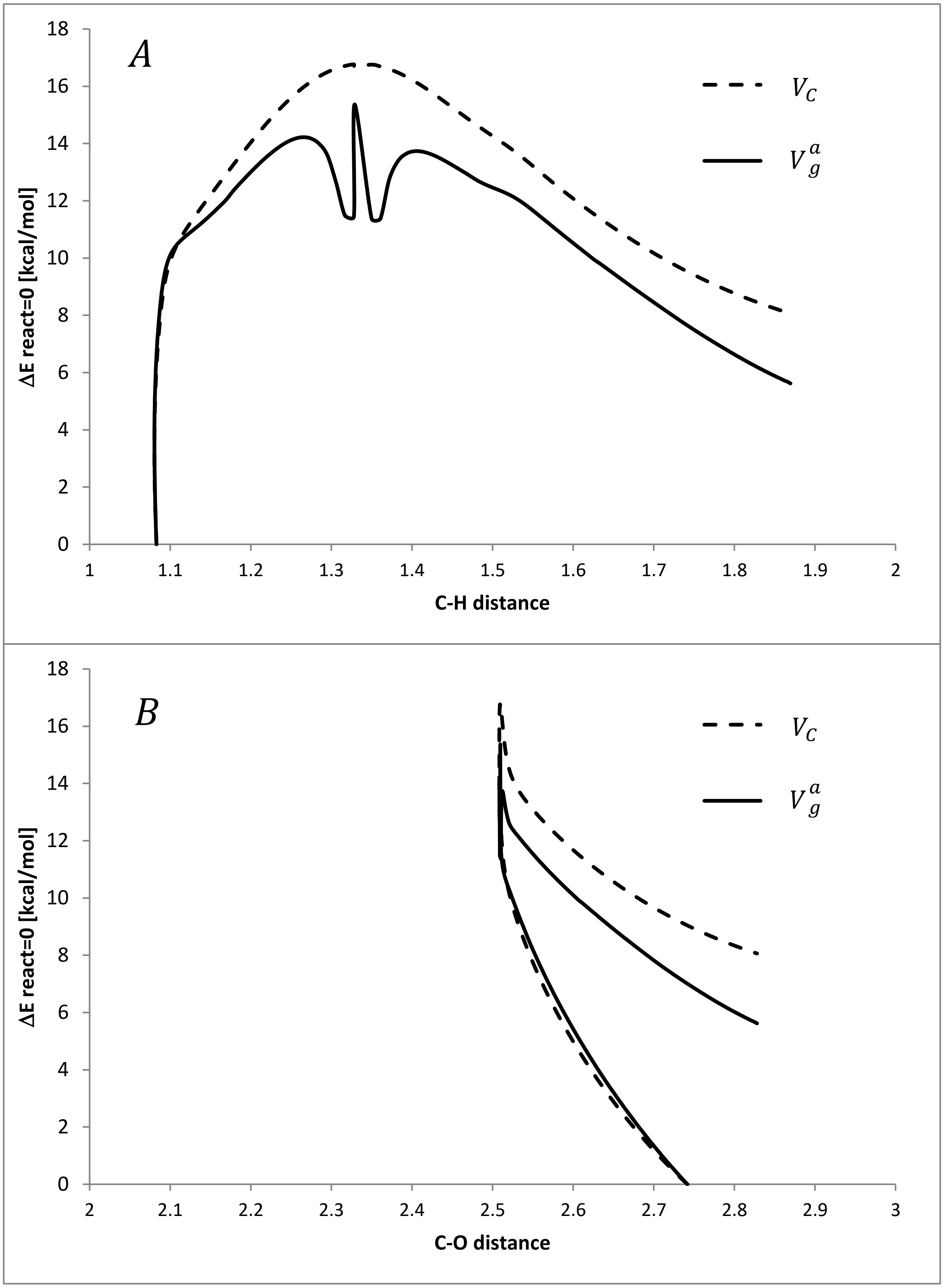
A
B
V_C
V_g^a
ΔE react=0 [kcal/mol]
C-H distance
C-O distance
0
2
4
6
8
10
12
14
16
18
1
1.1
1.2
1.3
1.4
1.5
1.6
1.7
1.8
1.9
2
2.1
2.2
2.3
2.4
2.5
2.6
2.7
2.8
2.9
3

Table S1: Geometry (Å) of OOH molecule, calculated at the M06-2X/aug-cc-pVTZ level of theory.

Atom	x	y	z
O	0.052464	-0.60022	0
H	-0.88402	-0.86266	0
O	0.055722	0.707768	0

Table S2: Frequencies (cm^{-1}) of OOH molecule, calculated at the M06-2X/aug-cc-pVTZ level of theory.

1256	1462	3696

Table S3: Geometry (Å) of H_2O_2 molecule, calculated at the M06-2X/aug-cc-pVTZ level of theory.

Atom	x	y	z
O	0.703375	0.135755	-3.6E-05
H	1.044471	-0.76441	4.05E-05
O	-0.70338	-0.13576	-3.5E-05
H	-1.04447	0.764409	5.75E-05

Table S4: Frequencies (cm^{-1}) of H_2O_2 molecule, calculated at the M06-2X/aug-cc-pVTZ level of theory.

1045	1266	1556	3862	3870

Table S5: Geometry (Å) of ethane molecule, calculated at the M06-2X/aug-cc-pVTZ level of theory.

Atom	x	y	z
C	-0.20994	0.023441	2.96E-08
H	0.137276	0.5649	0.879605
H	0.137276	0.564901	-0.87961
H	-1.29912	0.057078	-3.4E-08
C	0.297945	-1.41313	-3.4E-08
H	1.387125	-1.44677	2.98E-08
H	-0.04927	-1.95459	0.879605
H	-0.04927	-1.95459	-0.87961

Table S6: Frequencies (cm^{-1}) of ethane molecule, calculated at the M06-2X/aug-cc-pVTZ level of theory.

292	818	818	1022	1221	1221	1407	1426
1506	1506	1507	1507	3051	3053	3107	3107
3130	3130						

Table S7: Geometry (Å) of ethane + OOH transition state, calculated at the M06-2X/aug-cc-pVTZ level of theory.

Atom	x	y	z
C	-1.16157	0.692847	0.053974
H	-1.16282	1.193263	-0.91091
H	0.138205	0.588104	0.318536
H	-1.50232	1.33476	0.86192
O	1.281316	0.340734	0.497323
O	1.537011	-0.71204	-0.37154
H	1.91736	-0.27352	-1.14271
C	-1.66372	-0.71973	0.066538
H	-1.13379	-1.33185	-0.66229
H	-1.53755	-1.17443	1.047865
H	-2.7288	-0.75347	-0.18319

Table S7: Frequencies (cm^{-1}) of ethane + OOH transition state, calculated at the M06-2X/aug-cc-pVTZ level of theory.

30	129	136	238	402	529	576	825
869	1053	1073	1113	1193	1226	1400	1410
1448	1468	1481	1494	3029	3092	3105	3136
3179	3796	-1865					

Table S8: Geometry (Å) of ethyl molecule, calculated at the M06-2X/aug-cc-pVTZ level of theory.

Atom	x	y	z
C	-3.56239	1.536226	-0.01582
H	-3.20358	1.998665	0.904098
H	-3.30874	2.224386	-0.83247
H	-4.65095	1.490896	0.026693

C	-2.96616	0.192683	-0.22182
H	-1.95499	-0.0161	0.091545
H	-3.46836	-0.54702	-0.82587

Table S9: Frequencies (cm^{-1}) of ethyl molecule, calculated at the M06-2X/aug-cc-pVTZ level of theory.

80	427	803	985	1083	1193	1402	1471
1485	1486	2994	3069	3114	3164	3265	

Table S10: Geometry (Å) of propane molecule, calculated at the M06-2X/aug-cc-pVTZ level of theory.

Atom	x	y	z
C	-0.2254	0.022811	-3.6E-07
H	0.121699	0.566222	0.880387
H	0.121697	0.566221	-0.88039
H	-1.31489	0.043987	4.21E-07
C	0.3139	-1.40209	1.82E-07
H	-0.06385	-1.93629	0.874082
H	-0.06385	-1.93629	-0.87408
C	1.837073	-1.43561	-1E-06
H	2.220178	-2.45575	-2.5E-07
H	2.233718	-0.92724	-0.88039
H	2.23372	-0.92724	0.880386

Table S11: Frequencies (cm^{-1}) of propane molecule, calculated at the M06-2X/aug-cc-pVTZ level of theory.

250	303	375	754	894	916	942	1080
1186	1218	1324	1374	1411	1424	1497	1500
1501	1518	1519	3053	3054	3061	3086	3117
3127	3131	3133					

Table S12: Geometry (Å) of propane + OOH → 1-propyl + H_2O_2 transition state, calculated at the M06-2X/aug-cc-pVTZ level of theory.

Atom	x	y	z
C	1.155894	0.688397	0.275902
H	1.111186	0.829697	1.354197
H	-0.12811	0.73623	-0.07594
H	1.572298	1.548659	-0.24384
O	-1.26466	0.618189	-0.37442
O	-1.62973	-0.60515	0.172574
H	-2.04183	-0.35753	1.00959
C	1.634919	-0.65735	-0.18522
H	1.005391	-1.43622	0.247812
H	1.528416	-0.73169	-1.26819
C	3.096357	-0.89925	0.207028
H	3.219711	-0.85769	1.289381
H	3.430739	-1.87811	-0.13554
H	3.74686	-0.14369	-0.2338

Table S13: Frequencies (cm^{-1}) of propane + OOH → 1-propyl + H_2O_2 transition state, calculated at the M06-2X/aug-cc-pVTZ level of theory.

24	68	105	206	236	336	417	553
590	749	903	909	965	1069	1076	1125
1170	1213	1313	1344	1401	1413	1447	1463
1492	1499	1510	3046	3061	3088	3098	3124
3125	3171	3813	-1854				

Table S14: Geometry (Å) of 1-propyl radical, calculated at the M06-2X/aug-cc-pVTZ level of theory.

Atom	x	y	z
C	-3.56538	1.523401	0.015195
H	-3.36177	2.198329	-0.82787
H	-4.65453	1.436531	0.054605
C	-2.96539	0.189995	-0.24603
H	-1.97609	-0.04316	0.121117
H	-3.42596	-0.50434	-0.93228
C	-3.0454	2.163731	1.298519
H	-1.96207	2.282941	1.255871
H	-3.48661	3.146413	1.460212
H	-3.27791	1.539229	2.161114

Table S15: Frequencies (cm^{-1}) of 1-propyl radical, calculated at the M06-2X/aug-cc-pVTZ level of theory.

185	235	374	453	751	896	926	1052
1103	1177	1271	1360	1408	1470	1473	1499
1508	2979	3047	3049	3117	3127	3160	3262

Table S16: Geometry (Å) of propane + OOH → 2-propyl + H_2O_2 transition state, calculated at the M06-2X/aug-cc-pVTZ level of theory.

Atom	x	y	z
C	1.295641	0.732405	0.506643
H	1.645472	1.005565	1.501247
H	-0.00205	0.766006	0.664743
O	-1.20795	0.727673	0.58202
O	-1.42996	0.252851	-0.70296
H	-1.56865	-0.69308	-0.56804
C	1.584935	-0.6898	0.116635
H	1.292361	-1.39385	0.894606
H	1.062245	-0.94579	-0.80701
H	2.655049	-0.8253	-0.0717
C	1.541944	1.768763	-0.5534
H	0.965156	1.53641	-1.45022
H	1.266388	2.76616	-0.21606
H	2.600199	1.78503	-0.83462

Table S17: Frequencies (cm^{-1}) of propane + OOH → 2-propyl + H_2O_2 transition state, calculated at the M06-2X/aug-cc-pVTZ level of theory.

69	130	144	211	228	319	404	422
568	841	912	927	939	1076	1094	1160
1185	1198	1354	1404	1408	1414	1465	1479
1487	1492	1508	3027	3032	3089	3100	3107
3128	3138	3797	-1823				

Table S18: Geometry (Å) of 2-propyl radical, calculated at the M06-2X/aug-cc-pVTZ level of theory.

Atom	x	y	z
C	-3.2811	1.598122	-0.03437
H	-3.47372	2.250964	-0.87385
C	-3.03316	2.197049	1.302748
H	-1.9599	2.334099	1.493354
H	-3.50742	3.171348	1.409427
H	-3.40441	1.545993	2.09902
C	-2.91827	0.180349	-0.29195
H	-3.28955	-0.47039	0.504567
H	-3.31657	-0.17867	-1.23959
H	-1.82915	0.039074	-0.32143

Table S19: Frequencies (cm^{-1}) of 1-propyl radical, calculated at the M06-2X/aug-cc-pVTZ level of theory.

110	123	333	387	902	936	941	1026
1159	1183	1366	1410	1418	1469	1481	1486
1498	2986	2989	3049	3050	3118	3119	3211

Table S20: Geometry (Å) of butane molecule, calculated at the M06-2X/aug-cc-pVTZ level of theory.

Atom	x	y	z
C	-0.2261	0.033293	-7.4E-07
H	0.119819	0.577179	0.880548
H	0.119818	0.577176	-0.88055
H	-1.31565	0.055026	-2.9E-07
C	0.30961	-1.3923	8.78E-07
H	-0.06857	-1.92912	0.874529
H	-0.06857	-1.92912	-0.87453
C	1.83171	-1.44934	-1.5E-07
H	2.20989	-0.91252	-0.87453
H	2.209891	-0.91252	0.874526
C	2.367419	-2.87494	2.03E-06
H	2.021503	-3.41882	0.880554
H	2.021501	-3.41882	-0.88055
H	3.456972	-2.89667	7.51E-07

Table S21: Frequencies (cm^{-1}) of butane molecule, calculated at the M06-2X/aug-cc-pVTZ level of theory.

91	187	230	254	430	717	802	852
950	973	1041	1087	1175	1205	1283	1314
1328	1393	1405	1407	1484	1488	1497	1499
1504	1509	3026	3033	3036	3037	3051	3073
3104	3107	3111	3112				

Table S22: Geometry (Å) of butane + OOH → 1-butyl + H_2O_2 transition state, calculated at the M06-2X/aug-cc-pVTZ level of theory.

Atom	x	y	z
C	-0.43907	1.21842	0.389885
H	-0.41045	1.619574	1.400494
H	-1.67224	0.704221	0.322867
H	-0.47771	1.992583	-0.37278
O	-2.69149	0.121401	0.229864
O	-2.43841	-0.85605	-0.72332
H	-2.22586	-1.63276	-0.19079
C	0.489649	0.074894	0.111806
H	0.345192	-0.71338	0.856332
H	0.259629	-0.3606	-0.8636
C	1.962441	0.5054	0.130469
H	2.193818	0.944558	1.103405
H	2.110797	1.294322	-0.61
C	2.900667	-0.65989	-0.15287
H	2.696408	-1.09279	-1.13296
H	3.943229	-0.34442	-0.13604
H	2.776134	-1.44814	0.590835

Table S23: Frequencies (cm^{-1}) of butane + OOH → 1-butyl + H_2O_2 transition state, calculated at the M06-2X/aug-cc-pVTZ level of theory.

60	72	87	133	200	238	311	387
401	516	593	741	811	888	962	1014
1050	1076	1082	1123	1164	1214	1293	1313
1330	1374	1413	1414	1446	1469	1494	1500
1502	1515	3041	3048	3053	3078	3093	3098
3119	3127	3178	3810	-1851			

Table S24: Geometry (Å) of 1-butyl radical, calculated at the M06-2X/aug-cc-pVTZ level of theory.

Atom	x	y	z
C	-3.20577	2.166016	1.215327
H	-2.14114	2.208734	1.461223
H	-3.67158	1.49606	1.943274
C	-2.72615	0.146795	-0.24722
H	-3.1971	-0.52555	0.471567
H	-2.83344	-0.2931	-1.2382
H	-1.66155	0.18951	-0.0121
C	-3.80855	3.520636	1.304294
H	-3.25142	4.392838	0.996004
H	-4.86288	3.642436	1.503573
C	-3.35093	1.534065	-0.17982
H	-2.88585	2.192796	-0.91636
H	-4.41134	1.482428	-0.43587

Table S25: Frequencies (cm^{-1}) of 1-butyl radical, calculated at the M06-2X/aug-cc-pVTZ level of theory.

112	145	230	256	394	509	727	800
878	942	1048	1085	1100	1196	1281	1289
1325	1359	1407	1466	1489	1496	1500	1510
3028	3042	3047	3066	3087	3114	3122	3155
3256							

Table S26: Geometry (Å) of butane + OOH → 2-butyl + H_2O_2 transition state, calculated at the M06-2X/aug-cc-pVTZ level of theory.

Atom	x	y	z
C	0.404128	0.940402	0.2101
H	-0.83267	0.734603	-0.15729
H	0.636275	1.877069	-0.29809
O	-1.96906	0.368822	-0.35224
O	-2.10776	-0.72487	0.490905
H	-2.53199	-0.34894	1.272466
C	1.129892	-0.25937	-0.34138
H	0.659821	-1.16384	0.053043
H	1.012917	-0.28782	-1.42537

C	0.325217	1.057035	1.707199
H	1.305576	1.287627	2.13511
H	-0.35828	1.848922	2.012734
H	-0.00853	0.114673	2.146222
C	2.619805	-0.25602	0.013201
H	2.767998	-0.31025	1.091399
H	3.124257	-1.11026	-0.43669
H	3.103775	0.652159	-0.34841

Table S27: Frequencies (cm^{-1}) of butane + OOH → 2-butyl + H_2O_2 transition state, calculated at the M06-2X/aug-cc-pVTZ level of theory.

62	72	114	145	195	230	262	395
412	434	594	778	849	874	974	990
1007	1076	1105	1152	1166	1194	1292	1341
1382	1404	1408	1413	1464	1482	1492	1498
1503	1512	3032	3047	3050	3087	3091	3105
3121	3125	3128	3802	-1822			

Table S28: Geometry (Å) of 2-butyl radical, calculated at the M06-2X/aug-cc-pVTZ level of theory.

Atom	x	y	z
C	-3.34107	1.55077	-0.03634
H	-3.60338	2.193886	-0.86668
C	-3.15393	2.167223	1.304042
H	-2.08116	2.311671	1.501232
H	-3.48945	1.46481	2.075424
C	-2.85955	0.171259	-0.30484
H	-3.20431	-0.52312	0.466482
H	-3.19519	-0.19969	-1.27167
H	-1.76251	0.116462	-0.29699
C	-3.87806	3.499143	1.459821
H	-3.70842	3.933718	2.444028
H	-3.53211	4.213674	0.712042
H	-4.95216	3.37045	1.324759

Table S29: Frequencies (cm^{-1}) of 2-butyl radical, calculated at the M06-2X/aug-cc-pVTZ level of theory.

58	98	225	255	352	434	772	862
980	991	1051	1073	1145	1191	1275	1316
1406	1415	1432	1476	1482	1491	1501	1509
2969	2988	3032	3051	3057	3121	3124	3126
3191							

Table S30: Geometry (Å) of isobutane molecule, calculated at the M06-2X/aug-cc-pVTZ level of theory.

Atom	x	y	z
C	-0.18525	0.047383	-0.02508
H	0.164594	0.584278	0.857429
H	0.194627	0.56951	-0.90667
H	-1.27425	0.102495	-0.04765
C	0.296644	-1.39907	-0.0219
H	-0.10204	-1.89048	0.870284
C	1.818793	-1.45989	0.040323
H	2.251425	-0.97844	-0.84008
H	2.200825	-0.94638	0.923423
C	-0.22238	-2.14238	-1.2478
H	0.156622	-1.67555	-2.16022
H	0.100852	-3.18401	-1.24663
H	-1.312	-2.12433	-1.291
H	2.174144	-2.49067	0.065949

Table S31: Frequencies (cm^{-1}) of isobutane molecule, calculated at the M06-2X/aug-cc-pVTZ level of theory.

188	251	260	365	369	436	819	918
926	953	987	988	1200	1201	1213	1359
1360	1399	1401	1427	1482	1488	1490	1508
1509	1516	3031	3034	3035	3041	3102	3102
3110	3115	3117	3118				

Table S32: Geometry (Å) of isobutane + OOH → 1-isobutyl + H_2O_2 transition state, calculated at the M06-2X/aug-cc-pVTZ level of theory.

C	0.431914	1.337319	0.134979
H	0.475005	2.027833	-0.70469
H	1.668648	0.835833	0.180816
H	0.36489	1.843129	1.097757
O	2.706847	0.269876	0.172859
O	2.488499	-0.8273	-0.65131
H	2.822435	-0.53044	-1.50701
C	-0.47176	0.147085	-0.02868
H	-0.18288	-0.38138	-0.94125
C	-1.92259	0.61918	-0.19021
H	-2.03217	1.289803	-1.04245
H	-2.24795	1.151501	0.705466
C	-0.3374	-0.81176	1.148786
H	-0.61613	-0.30862	2.077753
H	-0.99328	-1.67342	1.023834
H	0.687321	-1.16812	1.2455
H	-2.58497	-0.23496	-0.3362

Table S33: Frequencies (cm^{-1}) of isobutane + OOH → 1-isobutyl + H_2O_2 transition state, calculated at the M06-2X/aug-cc-pVTZ level of theory.

66	86	105	216	244	278	340	375
415	429	533	587	834	929	937	963
980	1039	1075	1138	1174	1201	1215	1339
1376	1396	1408	1417	1442	1459	1490	1497
1509	1513	3045	3050	3064	3079	3118	3123
3129	3140	3164	3816	-1836			

Table S34: Geometry (Å) of 1-isobutyl radical, calculated at the M06-2X/aug-cc-pVTZ level of theory.

Atom	x	y	z
C	-1.72651	2.247214	1.599481
H	-1.30862	1.245243	1.699813
H	-1.12087	2.799085	0.878188
H	-1.64426	2.749646	2.563608
C	-3.17915	2.189817	1.129129
H	-3.74202	1.600675	1.868697

C	-3.77426	3.551809	1.057848
H	-3.47565	4.319328	1.757572
H	-4.62679	3.746476	0.422897
C	-3.29216	1.468243	-0.21291
H	-2.90176	0.452599	-0.1444
H	-4.3293	1.413775	-0.54474
H	-2.72141	2.002711	-0.97464

Table S35: Frequencies (cm^{-1}) of 1-isobutyl radical, calculated at the M06-2X/aug-cc-pVTZ level of theory.

167	220	257	361	378	405	516	834
911	946	976	994	1092	1191	1212	1330
1334	1396	1412	1468	1487	1492	1503	1512
2960	3040	3042	3112	3115	3119	3120	3149
3252							

Table S36: Geometry (Å) of isobutane + OOH → 2-isobutyl + H_2O_2 transition state, calculated at the M06-2X/aug-cc-pVTZ level of theory.

Atom	x	y	z
C	-0.66116	-0.44183	-0.06029
H	0.462337	-0.38463	0.568686
O	1.612178	-0.31974	1.002272
O	2.38163	-0.20719	-0.14598
H	2.525423	0.74425	-0.22384
C	-0.5256	-1.71479	-0.85575
H	-0.45994	-2.58424	-0.20241
H	-1.3974	-1.84584	-1.50671
H	0.363938	-1.68657	-1.48558
C	-1.70973	-0.47483	1.022982
H	-2.7064	-0.54154	0.571999
H	-1.58301	-1.34006	1.673329
H	-1.68201	0.427595	1.633599
C	-0.65456	0.813561	-0.89431
H	-1.5599	0.860829	-1.50986
H	0.201689	0.829561	-1.56992
H	-0.63117	1.706949	-0.26963

Table S37: Frequencies (cm^{-1}) of isobutane + OOH → 2-isobutyl + H_2O_2 transition state, calculated at the M06-2X/aug-cc-pVTZ level of theory.

57	114	124	203	222	240	246	345
368	388	416	575	814	928	944	969
1007	1022	1080	1134	1197	1276	1287	1392
1397	1411	1418	1455	1470	1483	1485	1496
1499	1513	3020	3021	3026	3089	3093	3105
3121	3128	3141	3785	-1778			

Table S38: Geometry (Å) of 2-isobutyl radical, calculated at the M06-2X/aug-cc-pVTZ level of theory.

Atom	x	y	z
C	-1.63271	2.265805	1.490717
H	-1.26843	1.239395	1.554802
H	-0.92659	2.813953	0.849338
H	-1.57243	2.716463	2.482481
C	-3.01864	2.321662	0.947615
C	-3.36262	1.440578	-0.20314
H	-2.94107	0.441488	-0.08298
H	-4.44248	1.347349	-0.32776
H	-2.96715	1.840534	-1.1487
C	-3.80181	3.574507	1.137082
H	-4.86713	3.410631	0.968095
H	-3.66972	3.981839	2.140554
H	-3.48521	4.357669	0.432198

Table S39: Frequencies (cm^{-1}) of 2-isobutyl radical, calculated at the M06-2X/aug-cc-pVTZ level of theory.

124	131	131	254	378	378	781	933
933	966	1016	1016	1100	1309	1309	1401
1401	1424	1470	1473	1473	1493	1495	1495
2969	2969	2975	3066	3066	3067	3111	3114
3114							

Table S40: Geometry (Å) of pentane molecule, calculated at the M06-2X/aug-cc-pVTZ level of theory.

C	-0.21993	-2.08629	-1.23869
H	0.327517	-1.91175	-2.16878
H	-0.08842	-3.14496	-0.99966
C	-1.69932	-1.80714	-1.47055
H	-1.83356	-0.74706	-1.70976
H	-2.24971	-1.98091	-0.54004
C	-2.30918	-2.65362	-2.58042
H	-1.75842	-2.47819	-3.50838
H	-2.17435	-3.7114	-2.33926
C	-3.78803	-2.35925	-2.79603
H	-3.93852	-1.31184	-3.06225
H	-4.21041	-2.9716	-3.59222
H	-4.35717	-2.55311	-1.88549
C	0.370136	-1.23009	-0.12564
H	1.428058	-1.44047	0.028828
H	0.269484	-0.16915	-0.35985
H	-0.14917	-1.41042	0.816907

Table S41: Frequencies (cm^{-1}) of pentane molecule, calculated at the M06-2X/aug-cc-pVTZ level of theory.

126	127	182	270	279	403	405	735
769	873	889	946	1003	1059	1070	1101
1175	1211	1274	1300	1336	1342	1377	1415
1417	1419	1490	1493	1502	1510	1510	1511
1518	3030	3045	3051	3055	3056	3058	3078
3092	3122	3123	3132	3132			

Table S42: Geometry (Å) of pentane + OOH → 1-pentyl + H_2O_2 transition state, calculated at the M06-2X/aug-cc-pVTZ level of theory.

Atom	x	y	z
C	0.402466	0.929273	0.229466
H	0.377407	1.0546	1.310267
H	-0.88525	0.790517	-0.07469
H	0.667071	1.84568	-0.2935
O	-2.00582	0.512313	-0.33146
O	-2.17175	-0.75152	0.219999

H	-2.57687	-0.56655	1.076306
C	1.055033	-0.32719	-0.26618
H	0.573215	-1.19585	0.188439
H	0.911738	-0.41655	-1.34516
C	2.555548	-0.36121	0.046353
H	2.703924	-0.26948	1.126334
H	3.039858	0.508226	-0.40755
C	3.226672	-1.63501	-0.45258
H	3.062859	-1.72689	-1.52916
H	2.740662	-2.4989	0.007492
C	4.719648	-1.66147	-0.15337
H	5.184391	-2.5777	-0.51593
H	5.224925	-0.81888	-0.62749
H	4.902039	-1.59526	0.920176

Table S43: Frequencies (cm^{-1}) of pentane + OOH → 1-pentyl + H_2O_2 transition state, calculated at the M06-2X/aug-cc-pVTZ level of theory.

29	56	80	110	130	155	236	242
360	403	448	553	593	730	768	869
902	973	999	1041	1073	1074	1084	1135
1168	1208	1270	1281	1325	1328	1353	1391
1413	1415	1446	1466	1488	1493	1503	1504
1514	3029	3043	3046	3051	3060	3081	3091
3098	3117	3120	3175	3801	-1856		

Table S44: Geometry (Å) of 1-pentyl radical, calculated at the M06-2X/aug-cc-pVTZ level of theory.

Atom	x	y	z
C	-3.20484	2.16724	1.220074
H	-2.14028	2.209609	1.465516
H	-3.67058	1.496999	1.947525
C	-2.73322	0.150327	-0.26446
H	-3.19965	-0.49814	0.481997
H	-1.67462	0.212022	0.001646
C	-3.80675	3.522024	1.312063
H	-3.24896	4.395461	1.008685
H	-4.85995	3.645273	1.516112
C	-3.35202	1.538976	-0.17604
H	-2.88715	2.197559	-0.91516

H	-4.41347	1.486798	-0.4344
C	-2.8811	-0.4681	-1.64824
H	-2.43357	-1.46051	-1.69459
H	-3.93331	-0.56196	-1.92087
H	-2.39836	0.152818	-2.40435

Table S45: Frequencies (cm^{-1}) of 1-pentyl radical, calculated at the M06-2X/aug-cc-pVTZ level of theory.

85	106	175	217	353	403	497	713
750	849	903	974	1004	1076	1091	1117
1195	1255	1256	1316	1321	1334	1380	1407
1468	1482	1487	1496	1498	1507	3024	3030
3036	3039	3052	3067	3085	3108	3113	3164
3267							

Table S46: Geometry (Å) of pentane + OOH →2-pentyl + H_2O_2 transition state, calculated at the M06-2X/aug-cc-pVTZ level of theory.

Atom	x	y	z
C	0.404214	0.920594	0.188118
H	-0.8365	0.731183	-0.17003
H	0.663882	1.820686	-0.3701
O	-1.98674	0.390878	-0.33919
O	-2.14555	-0.66505	0.547091
H	-2.54498	-0.24648	1.320097
C	1.096898	-0.32631	-0.29372
H	0.606344	-1.19906	0.148436
H	0.983644	-0.4152	-1.37649
C	2.589249	-0.3578	0.055382
H	2.709363	-0.32399	1.140086
H	3.069363	0.54085	-0.34042
C	3.274339	-1.60027	-0.49777
H	3.183934	-1.64116	-1.58386
H	4.334588	-1.61632	-0.24737
H	2.818634	-2.50516	-0.09381
C	0.325968	1.123666	1.676088
H	1.307354	1.371229	2.091766
H	-0.35068	1.938379	1.933667
H	-0.01625	0.211453	2.168786

Table S47: Frequencies (cm^{-1}) of pentane + OOH → 2-pentyl + H_2O_2 transition state, calculated at the M06-2X/aug-cc-pVTZ level of theory.

55	81	95	102	147	203	208	261
328	388	427	444	603	747	847	873
887	940	1014	1044	1059	1078	1112	1155
1166	1197	1270	1309	1330	1373	1389	1409
1412	1415	1460	1483	1490	1498	1500	1507
1514	3028	3038	3052	3054	3075	3087	3096
3110	3124	3132	3133	3800			

Table S48: Geometry (Å) of 2-pentyl radical, calculated at the M06-2X/aug-cc-pVTZ level of theory.

Atom	x	y	z
C	-3.07992	2.277881	1.03233
H	-2.21071	2.011814	1.620437
C	-2.70262	0.179703	-0.3152
H	-3.1564	-0.4187	0.477949
H	-1.63448	0.231961	-0.08962
C	-3.28805	1.587336	-0.26754
H	-2.84945	2.179485	-1.0869
H	-4.3611	1.546489	-0.49158
C	-2.9126	-0.49548	-1.66322
H	-2.49434	-1.50139	-1.67785
H	-3.97549	-0.57177	-1.89786
H	-2.43821	0.076346	-2.46226
C	-3.82048	3.5249	1.355459
H	-3.68543	3.822783	2.393863
H	-3.4933	4.366084	0.729571
H	-4.89142	3.404728	1.169917

Table S49: Frequencies (cm^{-1}) of 2-pentyl radical, calculated at the M06-2X/aug-cc-pVTZ level of theory.

19	91	131	181	214	374	403	408
729	855	882	946	982	1050	1079	1097
1149	1183	1249	1281	1317	1365	1405	1410

1431	1471	1477	1492	1495	1499	1510	2957
2989	3017	3039	3042	3051	3078	3110	3118
3121	3193						

Table S50: Geometry (Å) of pentane + OOH →3-pentyl + H_2O_2 transition state, calculated at the M06-2X/aug-cc-pVTZ level of theory.

Atom	x	y	z
C	0.384317	0.894254	0.207043
H	-0.84479	0.657118	-0.18089
H	0.600485	1.838886	-0.29726
O	-1.97557	0.273665	-0.37948
O	-2.16584	-0.69419	0.596879
H	-2.60886	-0.21075	1.306415
C	1.152912	-0.28535	-0.32886
H	0.731023	-1.20058	0.093544
H	1.018557	-0.34414	-1.40978
C	0.2601	1.02247	1.701327
H	1.252763	1.203026	2.130448
H	-0.07449	0.064257	2.109266
C	2.648245	-0.20782	-0.00653
H	2.8212	-0.22368	1.069131
H	3.180552	-1.05199	-0.4434
H	3.08348	0.709563	-0.40493
C	-0.68368	2.142116	2.124129
H	-0.77109	2.203066	3.207967
H	-0.32645	3.106413	1.760912
H	-1.67996	1.989892	1.705145

Table S51: Frequencies (cm^{-1}) of pentane + OOH → 3-pentyl + H_2O_2 transition state, calculated at the M06-2X/aug-cc-pVTZ level of theory.

37	67	109	123	147	199	221	244
314	389	434	451	592	758	769	869
918	936	1008	1029	1058	1074	1102	1159
1164	1191	1267	1291	1332	1359	1399	1406
1410	1418	1454	1472	1492	1499	1501	1510
1512	3017	3047	3048	3057	3059	3082	3100
3116	3122	3125	3128	3789	-1841		

Table S52: Geometry (Å) of 3-pentyl radical, calculated at the M06-2X/aug-cc-pVTZ level of theory.

Atom	x	y	z
C	-2.64979	0.146024	-0.3113
H	-2.90087	-0.60289	0.454197
H	-1.57081	0.305689	-0.20569
C	-3.3655	1.418226	-0.02756
H	-4.26726	1.642915	-0.58644
C	-2.96053	-0.42048	-1.69183
H	-2.42968	-1.3545	-1.8709
H	-4.0287	-0.61517	-1.79441
H	-2.67566	0.287963	-2.46996
C	-3.75924	3.573689	1.202702
H	-3.52735	4.134649	2.107031
H	-3.452	4.17023	0.343479
H	-4.84158	3.449824	1.151452
C	-3.06399	2.217296	1.189734
H	-1.97978	2.350857	1.276419
H	-3.35185	1.652339	2.088542

Table S53: Frequencies (cm^{-1}) of 3-pentyl radical, calculated at the M06-2X/aug-cc-pVTZ level of theory.

39	76	181	219	224	399	401	412
752	783	885	960	1031	1055	1070	1086
1154	1180	1268	1276	1282	1371	1415	1419
1436	1471	1482	1500	1500	1506	1506	2973
2976	3028	3030	3049	3050	3119	3119	3125
3125	3157						

Table S54: Geometry (Å) of isopentane molecule, calculated at the M06-2X/aug-cc-pVTZ level of theory.

Atom	x	y	z
C	-0.17658	0.037223	-0.01096
H	0.209145	0.560421	0.864874
H	0.172695	0.56891	-0.89942
H	-1.26509	0.099622	0.007791
C	0.296073	-1.41217	-0.04429
H	-0.07581	-1.91682	0.854008
C	1.823632	-1.4716	-0.01033

H	2.211251	-0.95314	-0.89348
H	2.17293	-0.9044	0.856152
C	2.394015	-2.88323	0.049284
H	1.956105	-3.4427	0.878328
H	2.194684	-3.43747	-0.86737
H	3.473855	-2.86533	0.193341
C	-0.27617	-2.12752	-1.26401
H	0.123955	-1.68751	-2.18136
H	-0.03474	-3.18978	-1.26701
H	-1.36209	-2.03226	-1.29514

Table S55: Frequencies (cm^{-1}) of isopentane molecule, calculated at the M06-2X/aug-cc-pVTZ level of theory.

99	205	232	270	285	375	418	459
771	804	928	933	971	984	1035	1069
1178	1204	1210	1298	1326	1371	1388	1403
1410	1420	1482	1491	1498	1502	1508	1510
1520	3022	3034	3038	3044	3052	3075	3105
3111	3118	3118	3127	3136			

Table S56: Geometry (Å) of isopentane + OOH → 2-methyl-but-1-yl + H_2O_2 transition state (R11), calculated at the M06-2X/aug-cc-pVTZ level of theory.

Atom	x	y	z
C	0.461728	0.975425	0.238244
H	0.54841	1.139748	1.310337
H	-0.85664	0.877116	0.050783
H	0.699811	1.86246	-0.34794
O	-2.00304	0.656179	-0.13956
O	-2.10591	-0.72504	-0.02564
H	-2.39516	-0.84986	0.886809
C	1.033631	-0.3121	-0.28555
H	0.562152	-1.14336	0.24924
C	3.210437	-1.66118	-0.40206
H	4.251333	-1.68375	-0.08079
H	2.70349	-2.5203	0.040628
H	3.195777	-1.78513	-1.48382
C	2.541903	-0.3587	0.021131
H	2.685554	-0.20777	1.093378
H	3.024342	0.485656	-0.47989
C	0.740685	-0.46798	-1.77429

H	1.26943	0.299303	-2.34572
H	1.055666	-1.44272	-2.14296
H	-0.32657	-0.36419	-1.96618

Table S57: Frequencies (cm^{-1}) of isopentane + OOH → 2-methyl-but-1-yl + H_2O_2 transition state (R11), calculated at the M06-2X/aug-cc-pVTZ level of theory.

58	67	88	97	194	206	266	291
353	397	423	440	527	591	770	823
919	933	977	1022	1032	1071	1076	1142
1164	1192	1207	1288	1319	1361	1373	1405
1410	1412	1440	1459	1485	1497	1498	1507
1518	3030	3038	3047	3053	3075	3080	3119
3122	3128	3138	3166	3814	-1866		

Table S58: Geometry (Å) of 2-methyl-but-1-yl radical, calculated at the M06-2X/aug-cc-pVTZ level of theory.

Atom	x	y	z
C	-1.662	2.735032	1.296841
H	-0.77941	2.103999	1.198677
H	-1.71547	3.38018	0.418384
H	-1.51495	3.371749	2.169123
C	-2.92361	1.889549	1.427995
H	-2.97195	1.178239	0.599909
H	-2.87526	1.297256	2.344818
C	-4.27628	3.701391	2.597596
H	-4.09415	3.201357	3.549776
H	-3.52627	4.482977	2.47266
H	-5.25255	4.183886	2.647743
C	-4.45137	3.387747	0.1338
H	-4.20862	2.888087	-0.79372
H	-5.01611	4.307159	0.078522
C	-4.22468	2.707473	1.438158
H	-5.03933	1.984154	1.593182

Table S59: Frequencies (cm^{-1}) of 2-methyl-but-1-yl radical, calculated at the M06-2X/aug-cc-pVTZ level of theory.

106	209	241	273	296	376	387	499

590	783	801	920	954	995	1033	1040
1092	1182	1199	1282	1317	1358	1371	1411
1412	1470	1489	1494	1502	1511	1517	2967
3040	3050	3053	3077	3117	3122	3126	3133
3164	3266						

Table S60: Geometry (Å) of isopentane + OOH → 2-methyl-but-2-yl + H_2O_2 (R12) transition state, calculated at the M06-2X/aug-cc-pVTZ level of theory.

Atom	x	y	z
C	0.410693	0.968966	0.177325
H	-0.815	0.73542	-0.12772
O	-1.98891	0.390726	-0.2961
O	-2.12127	-0.71206	0.533507
H	-2.50134	-0.33975	1.339112
C	0.738659	2.251247	-0.54767
H	0.668597	2.125558	-1.62811
H	0.068027	3.056663	-0.24734
H	1.759075	2.572064	-0.31217
C	0.367756	1.095796	1.679861
H	1.355004	1.360466	2.072377
H	-0.32421	1.881266	1.986767
H	0.066427	0.156026	2.145029
C	1.102196	-0.27246	-0.34252
H	0.611458	-1.15115	0.082394
H	0.966882	-0.32594	-1.42455
C	2.597301	-0.3117	-0.01269
H	2.76516	-0.32654	1.064309
H	3.058025	-1.2051	-0.43218
H	3.115223	0.555199	-0.4239

Table S61: Frequencies (cm^{-1}) of isopentane + OOH → 2-methyl-but-2-yl + H_2O_2 (R12) transition state, calculated at the M06-2X/aug-cc-pVTZ level of theory.

70	74	103	128	196	226	227	251
268	364	395	422	449	625	786	795
935	943	990	1018	1047	1056	1085	1146
1164	1236	1284	1336	1362	1396	1401	1410
1415	1453	1479	1484	1485	1498	1507	1509
1512	3024	3030	3050	3055	3090	3094	3097
3125	3126	3131	3132	3798	-1863		

Table S62: Geometry (Å) of 2-methyl-but-2-yl radical, calculated at the M06-2X/aug-cc-pVTZ level of theory.

Atom	x	y	z
C	-1.51236	2.39716	1.485225
H	-0.83108	1.631627	1.116209
H	-1.09168	3.373595	1.241159
H	-1.54947	2.311123	2.570868
C	-3.88062	3.301339	1.229086
C	-2.89687	2.235211	0.865533
H	-2.80901	2.194355	-0.22366
H	-3.30231	1.25307	1.159137
C	-3.90072	3.83478	2.621263
H	-4.24827	3.075313	3.338187
H	-2.9116	4.149635	2.955699
H	-4.57563	4.686478	2.706419
C	-5.14159	3.361824	0.43676
H	-5.65244	4.317161	0.564387
H	-4.95342	3.2096	-0.62723
H	-5.84967	2.579463	0.748654

Table S63: Frequencies (cm^{-1}) of 2-methyl-but-2-yl radical, calculated at the M06-2X/aug-cc-pVTZ level of theory.

62	144	157	219	285	296	387	455
752	759	938	950	992	998	1047	1080
1114	1255	1284	1317	1383	1403	1407	1418
1467	1478	1484	1493	1494	1502	1511	2952
2970	2976	3049	3052	3077	3081	3120	3124
3127	3129						

Table S64: Geometry (Å) of isopentane + OOH → 3-methyl-but-2-yl + H_2O_2 (R13) transition state, calculated at the M06-2X/aug-cc-pVTZ level of theory.

Atom	x	y	z
C	0.373129	0.997264	0.159441
H	-0.84505	0.630831	-0.15305
H	0.477196	1.928432	-0.40214
O	-1.93782	0.12749	-0.28646
O	-1.9716	-0.83946	0.708515

H	-2.42114	-0.393	1.437801
C	0.313023	1.208149	1.652899
H	0.029073	0.253772	2.108885
C	-0.72777	2.262528	2.01666
H	-0.81104	2.375306	3.097677
H	-0.44393	3.23171	1.600311
H	-1.70971	2.007767	1.614854
C	1.214324	-0.13979	-0.35013
H	1.068973	-0.29294	-1.41823
H	2.278574	0.05287	-0.18532
H	0.95585	-1.06469	0.168013
C	1.689069	1.602088	2.202816
H	2.036544	2.521847	1.72751
H	1.631991	1.778577	3.277427
H	2.431239	0.824907	2.026154

Table S65: Frequencies (cm^{-1}) of isopentane + OOH → 3-methyl-but-2-yl + H_2O_2 (R13) transition state, calculated at the M06-2X/aug-cc-pVTZ level of theory.

50	70	107	132	197	204	230	255
334	360	401	408	480	593	813	855
932	935	968	994	1064	1076	1087	1153
1172	1194	1208	1304	1347	1386	1401	1409
1412	1419	1455	1485	1490	1491	1496	1503
1515	3032	3035	3038	3044	3092	3103	3106
3114	3121	3130	3144	3785	-1839		

Table S66: Geometry (Å) of 3-methyl-but-2-yl radical, calculated at the M06-2X/aug-cc-pVTZ level of theory.

Atom	x	y	z
C	-1.70245	2.527161	1.581839
H	-0.92791	1.769599	1.482712
H	-1.59288	3.224701	0.741668
H	-1.48891	3.106756	2.485521
C	-3.06093	1.920265	1.622779
H	-3.16854	0.863338	1.419907
C	-4.16373	4.003652	2.492673
H	-3.90745	3.730423	3.516695
H	-3.38626	4.671491	2.117082
H	-5.09915	4.563322	2.51002
C	-4.29398	2.767224	1.603647

H	-5.12482	2.163062	1.978064
C	-4.64194	3.187412	0.165962
H	-5.55569	3.783781	0.146042
H	-3.83646	3.790929	-0.25718
H	-4.78415	2.31685	-0.47388

Table S67: Frequencies (cm^{-1}) of 3-methyl-but-2-yl radical, calculated at the M06-2X/aug-cc-pVTZ level of theory.

78	95	229	265	280	286	370	470
537	795	912	925	961	984	1024	1070
1118	1170	1211	1326	1366	1369	1389	1412
1420	1475	1485	1488	1496	1507	1512	2999
3036	3040	3044	3049	3105	3113	3124	3126
3127	3179						

Table S68: Geometry (Å) of isopentane + OOH → 3-methyl-but-1-yl + H_2O_2 (R14) transition state, calculated at the M06-2X/aug-cc-pVTZ level of theory.

Atom	x	y	z
C	0.375887	0.979556	0.086187
H	0.415092	1.144077	1.162132
H	-0.91559	0.735068	-0.10569
H	0.527003	1.891703	-0.48485
O	-2.03227	0.355205	-0.22462
O	-2.04675	-0.85891	0.44848
H	-2.38476	-0.62339	1.321439
C	1.068714	-0.25855	-0.40252
H	0.549691	-1.13641	-0.00807
H	1.004061	-0.31523	-1.49246
C	2.549268	-0.33702	0.008988
H	2.591041	-0.30116	1.102177
C	3.34688	0.838706	-0.54403
H	4.401404	0.751538	-0.28088
H	3.275029	0.863718	-1.63437
H	2.982955	1.791903	-0.16091
C	3.146362	-1.65987	-0.45614
H	4.183722	-1.7572	-0.13439
H	2.585904	-2.50856	-0.0633
H	3.127671	-1.7206	-1.54663

Table S69: Frequencies (cm^{-1}) of isopentane + OOH → 3-methyl-but-1-yl + H_2O_2 (R14) transition state, calculated at the M06-2X/aug-cc-pVTZ level of theory.

34	61	91	115	184	241	263	272
364	411	441	456	565	598	794	816
931	950	969	978	1033	1073	1074	1144
1175	1194	1206	1295	1314	1361	1367	1403
1413	1420	1446	1464	1484	1492	1498	1508
1514	3034	3040	3042	3047	3083	3094	3110
3115	3124	3128	3178	3804	-1852		

Table S70: Geometry (Å) of 3-methyl-but-1-yl radical, calculated at the M06-2X/aug-cc-pVTZ level of theory.

Atom	x	y	z
C	-2.1596	2.251552	2.487856
H	-1.31261	2.080283	1.841165
H	-1.98843	2.804646	3.398695
C	-4.53396	4.147737	2.029335
H	-5.03436	3.958103	2.980254
H	-3.5813	4.637145	2.240673
H	-5.14676	4.844699	1.456758
C	-4.30972	2.855955	1.252509
H	-5.28528	2.409562	1.040056
C	-3.6125	3.134372	-0.07365
H	-4.20549	3.810397	-0.69049
H	-2.6413	3.603201	0.095721
H	-3.4498	2.214862	-0.63812
C	-3.52719	1.820342	2.094553
H	-3.46815	0.89465	1.516053
H	-4.11542	1.603231	2.989838

Table S71: Frequencies (cm^{-1}) of 3-methyl-but-1-yl radical, calculated at the M06-2X/aug-cc-pVTZ level of theory.

107	178	237	252	277	362	377	472
591	787	792	917	957	970	1019	1039
1124	1184	1204	1292	1294	1369	1371	1401
1417	1470	1486	1493	1496	1509	1510	3034

3039	3043	3048	3088	3108	3112	3117	3119
3163	3262						

Table S72: Geometry (Å) of 2,2-dimethylpropane molecule, calculated at the M06-2X/aug-cc-pVTZ level of theory.

Atom	x	y	z
C	-2.3E-05	-7.4E-05	0.000163
C	-0.51831	-0.45071	1.365154
H	-0.02528	-1.37124	1.68331
H	-0.33196	0.312484	2.123126
C	-0.27669	-1.0895	-1.035
H	-1.34745	-1.286	-1.11585
H	-1.59339	-0.63722	1.330436
H	0.21865	-2.02243	-0.75946
C	1.504686	0.251934	0.084234
H	1.728373	1.02756	0.819127
H	2.034777	-0.65587	0.378471
H	1.898638	0.576273	-0.88079
C	-0.70962	1.288191	-0.41439
H	-0.35409	1.631373	-1.38778
H	-1.78801	1.133164	-0.48313
H	-0.52776	2.082773	0.311724
H	0.087842	-0.79102	-2.01975

Table S73: Frequencies (cm^{-1}) of 2,2-dimethylpropane molecule, calculated at the M06-2X/aug-cc-pVTZ level of theory.

200	282	288	292	336	337	417	420
422	752	948	950	951	960	962	964
1090	1090	1290	1291	1292	1398	1399	1400
1436	1482	1483	1484	1490	1492	1519	1520
1520	3034	3034	3034	3042	3105	3106	3108
3108	3110	3112	3113	3115			

Table S74: Geometry (Å) of 2,2-dimethylpropane + OOH → 2,2-dimethylpropyl + H_2O_2 transition state (R15), calculated at the M06-2X/aug-cc-pVTZ level of theory.

Atom	x	y	z
C	0.512326	1.119802	0.098583

H	0.678524	1.72777	-0.79017
H	1.678875	0.481513	0.190921
H	0.47728	1.702239	1.017995
O	2.694904	-0.12557	0.183463
O	3.085962	-0.12188	-1.15048
H	3.707742	0.614956	-1.19522
C	-0.54079	0.047592	-0.03355
C	-1.9065	0.739244	-0.16565
H	-1.9376	1.374999	-1.05162
H	-2.11983	1.356401	0.708335
C	-0.53704	-0.84035	1.211063
H	-0.7265	-0.25121	2.110317
H	-1.31386	-1.6027	1.135577
H	0.425347	-1.34112	1.324019
H	-2.6955	-0.01017	-0.25552
C	-0.27421	-0.79991	-1.2776
H	0.689553	-1.30425	-1.20424
H	-1.05391	-1.55448	-1.39283
H	-0.26517	-0.18019	-2.17609

Table S75: Frequencies (cm^{-1}) of of 2,2-dimethylpropane + OOH → 2,2-dimethylpropyl + H_2O_2 transition state (R15), calculated at the M06-2X/aug-cc-pVTZ level of theory.

52	58	110	168	221	276	285	306
330	392	396	408	439	571	644	760
931	943	949	961	964	965	1060	1067
1087	1177	1250	1290	1292	1390	1401	1404
1420	1436	1453	1482	1485	1488	1508	1511
1519	3036	3038	3043	3079	3110	3111	3115
3116	3122	3127	3164	3809	-1872		

Table S76: Geometry (Å) of 2,2-dimethylpropyl radical, calculated at the M06-2X/aug-cc-pVTZ level of theory.

Atom	x	y	z
C	-2.66647	0.13778	-0.31149
C	-2.98121	-0.46069	-1.69403
H	-2.4565	-1.40662	-1.83423
H	-4.05101	-0.64457	-1.8011
H	-2.66919	0.226533	-2.48401
C	-3.41792	1.461923	-0.15704
H	-3.20189	1.917712	0.810156

H	-3.12602	2.163059	-0.94093
H	-4.49577	1.304888	-0.22646
C	-3.10081	-0.81726	0.751852
H	-2.45728	-1.63502	1.045942
H	-4.13079	-0.83521	1.080712
C	-1.1597	0.382673	-0.20396
H	-0.60779	-0.55326	-0.30724
H	-0.82401	1.06288	-0.98876
H	-0.90802	0.821426	0.762496

Table S77: Frequencies (cm^{-1}) of 2,2-dimethylpropyl radical, calculated at the M06-2X/aug-cc-pVTZ level of theory.

132	206	268	278	310	336	386	416
416	524	762	926	938	954	960	964
1034	1076	1222	1284	1297	1385	1396	1416
1465	1477	1485	1485	1502	1502	1520	3030
3034	3039	3102	3108	3111	3112	3116	3116
3142	3247						

Table S78: Geometry (Å) (cm^{-1}) of hexane molecule, calculated at the M06-2X/aug-cc-pVTZ level of theory.

Atom	x	y	z
C	0.381144	-1.23959	-0.13174
H	-0.16974	-1.415	0.796129
C	1.859954	-1.53359	0.084258
H	2.429282	-1.33966	-0.82616
H	2.282003	-0.92105	0.880523
C	-0.22861	-2.08628	-1.24177
H	0.322159	-1.91207	-2.17182
H	-0.09403	-3.14603	-1.00199
H	2.010626	-2.58094	0.350616
C	-1.70757	-1.80589	-1.47217
H	-1.84215	-0.74613	-1.71196
H	-2.25834	-1.9801	-0.54212
C	-2.31732	-2.65257	-2.5822
H	-1.76644	-2.47717	-3.51007
H	-2.18234	-3.7103	-2.34103
C	-3.79613	-2.35858	-2.7982
H	-3.9468	-1.31122	-3.06456

H	-4.21818	-2.97111	-3.59447
H	-4.36546	-2.55251	-1.88779
H	0.246165	-0.18186	-0.37292

Table S79: Frequencies (cm^{-1}) of hexane molecule, calculated at the M06-2X/aug-cc-pVTZ level of theory.

81	126	134	159	269	278	302	373
473	733	748	813	909	916	921	1024
1040	1076	1095	1098	1172	1210	1258	1281
1317	1341	1347	1349	1399	1416	1417	1421
1489	1490	1497	1504	1510	1510	1513	1518
3028	3034	3048	3050	3055	3055	3055	3068
3084	3093	3122	3122	3132	3132		

Table S80: Geometry (Å) of hexane + OOH → 1-hexyl + H_2O_2 transition state (R16), calculated at the M06-2X/aug-cc-pVTZ level of theory.

Atom	x	y	z
C	0.393076	0.932995	0.180404
H	0.374608	1.064008	1.260827
H	-0.89778	0.78328	-0.10333
H	0.65294	1.846773	-0.34928
O	-2.02325	0.484722	-0.31195
O	-2.17615	-0.72352	0.355941
H	-2.56315	-0.46102	1.20037
C	1.045815	-0.32568	-0.30963
H	0.546415	-1.19284	0.129014
H	0.926701	-0.40704	-1.39201
C	2.538127	-0.37331	0.03897
H	2.657499	-0.30061	1.123793
H	3.037386	0.501908	-0.38651
C	3.21593	-1.64101	-0.46435
H	3.092699	-1.71124	-1.54944
H	2.710482	-2.51478	-0.04175
C	4.698939	-1.70075	-0.12064
H	5.199117	-0.82324	-0.53862
H	4.818221	-1.63435	0.963902
C	5.367264	-2.96851	-0.63584
H	5.284324	-3.03624	-1.72146
H	6.425275	-2.99855	-0.37778
H	4.893677	-3.85572	-0.21309

Table S81: Frequencies (cm^{-1}) of hexane + OOH → 1-hexyl + H_2O_2 transition state (R16), calculated at the M06-2X/aug-cc-pVTZ level of theory.

48	56	78	85	123	137	150	211
253	285	371	417	455	563	598	732
752	812	913	915	957	1023	1047	1057
1074	1079	1091	1142	1172	1208	1257	1268
1312	1331	1336	1339	1379	1407	1411	1414
1443	1464	1489	1491	1499	1504	1510	1518
3027	3034	3043	3049	3052	3055	3069	3085
3093	3099	3117	3125	3177	3805	-1864	

Table S82: Geometry (Å) of 1-hexyl radical, calculated at the M06-2X/aug-cc-pVTZ level of theory.

Atom	x	y	z
C	-3.45164	1.384037	-0.41387
H	-3.61557	1.996855	0.47381
H	-3.01435	2.019394	-1.18332
H	-4.4278	1.050245	-0.76891
C	-2.55834	0.193768	-0.09025
H	-1.57781	0.546782	0.239709
H	-2.38398	-0.39421	-0.99508
C	-2.26947	-1.90153	1.321339
H	-2.09228	-2.4924	0.418375
H	-1.28921	-1.54782	1.65515
C	-2.86753	-2.80151	2.399166
H	-3.0614	-2.19111	3.294159
H	-3.85175	-3.15555	2.077005
C	-2.00124	-3.95661	2.745623
H	-0.92762	-3.87941	2.649581
H	-2.40172	-4.82464	3.246755
C	-3.15156	-0.70762	0.984817
H	-3.32847	-0.12021	1.891583
H	-4.1332	-1.06331	0.65553

Table S83: Frequencies (cm^{-1}) of 1-hexyl radical, calculated at the M06-2X/aug-cc-pVTZ level of theory.

66	106	132	149	218	244	302	374
459	495	719	739	824	900	913	968

1050	1061	1093	1103	1114	1172	1225	1268
1291	1328	1336	1341	1392	1412	1415	1468
1472	1487	1492	1501	1506	1514	2973	3024
3033	3041	3044	3047	3054	3074	3088	3114
3124	3160	3261					

Table S84: Geometry (Å) of hexane + OOH → 2-hexyl + H_2O_2 transition state (R17), calculated at the M06-2X/aug-cc-pVTZ level of theory.

Atom	x	y	z
C	0.394419	0.916149	0.181227
H	-0.84805	0.727489	-0.17097
H	0.655095	1.811022	-0.3849
O	-1.99953	0.388643	-0.33507
O	-2.16257	-0.65491	0.565137
H	-2.56072	-0.22478	1.332405
C	1.081298	-0.33644	-0.29469
H	0.586405	-1.20423	0.151914
H	0.966141	-0.42897	-1.37684
C	2.57302	-0.37205	0.054032
H	2.697867	-0.34547	1.139896
H	3.060542	0.526269	-0.3375
C	3.270438	-1.60861	-0.49932
H	3.148916	-1.63144	-1.58502
H	2.771323	-2.50163	-0.1151
C	0.322761	1.130903	1.667778
H	1.307805	1.372847	2.077888
H	-0.34592	1.953625	1.920566
H	-0.02618	0.225795	2.168799
C	4.750784	-1.64923	-0.14389
H	5.236836	-2.53607	-0.54894
H	5.268036	-0.77356	-0.53873
H	4.88976	-1.65528	0.938164

Table S85: Frequencies (cm^{-1}) of hexane + OOH → 2-hexyl + H_2O_2 transition state (R17), calculated at the M06-2X/aug-cc-pVTZ level of theory.

54	63	92	108	127	151	158	207
269	283	365	415	426	443	599	739
790	855	912	913	927	1007	1029	1070
1077	1086	1111	1157	1165	1196	1254	1285
1318	1337	1355	1385	1400	1408	1413	1420
1460	1481	1492	1493	1497	1504	1508	1515

3028	3035	3043	3052	3057	3068	3081	3088
3096	3103	3125	3127	3136	3797	-1833	

Table S86: Geometry (Å) of 2-hexyl radical, calculated at the M06-2X/aug-cc-pVTZ level of theory.

C	-3.49179	1.356252	-0.44976
H	-3.63894	2.00547	0.414651
H	-3.09833	1.965221	-1.263
H	-4.4717	0.984875	-0.75324
C	-2.55916	0.202098	-0.10795
H	-1.5742	0.590324	0.164519
H	-2.40786	-0.4242	-0.99116
C	-2.17246	-1.83146	1.368484
H	-2.03206	-2.4571	0.478401
H	-1.16867	-1.44083	1.60031
C	-2.66385	-2.67333	2.490923
H	-3.30327	-2.21587	3.23499
C	-3.08812	-0.6606	1.029911
H	-3.22443	-0.04076	1.921507
H	-4.07853	-1.04389	0.76817
C	-2.02812	-3.98368	2.785064
H	-2.58329	-4.55008	3.530799
H	-1.94877	-4.59358	1.880852
H	-1.00408	-3.86169	3.163076

Table S87: Frequencies (cm^{-1}) of 2-hexyl radical, calculated at the M06-2X/aug-cc-pVTZ level of theory.

50	100	114	130	153	243	298	363
382	468	721	778	910	914	924	990
1049	1072	1092	1106	1150	1181	1234	1267
1306	1330	1336	1394	1412	1413	1432	1471
1482	1489	1491	1498	1505	1513	2958	2997
3018	3029	3040	3047	3053	3060	3082	3114
3123	3133	3177					

Table S88: Geometry (Å) of hexane + OOH → 3-hexyl + H_2O_2 transition state (R18), calculated at the M06-2X/aug-cc-pVTZ level of theory.

Atom	x	y	z

C	0.360827	0.87675	0.18399
H	-0.8794	0.644668	-0.16909
H	0.585565	1.793585	-0.36533
O	-2.01969	0.260938	-0.31209
O	-2.16456	-0.69397	0.684514
H	-2.57091	-0.20026	1.408854
C	1.094161	-0.34228	-0.30958
H	0.604827	-1.2365	0.088911
H	1.01928	-0.3996	-1.39765
C	2.573955	-0.36075	0.09143
H	2.652314	-0.39723	1.179712
H	3.045529	0.573509	-0.2245
C	3.310547	-1.5467	-0.51622
H	3.281057	-1.50469	-1.60564
H	4.355509	-1.56874	-0.2086
H	2.849367	-2.4863	-0.20927
C	0.272104	1.074779	1.673351
H	1.275387	1.282393	2.06329
H	-0.04204	0.134733	2.136483
C	-0.66794	2.207683	2.068323
H	-0.71066	2.332852	3.149244
H	-0.33783	3.151885	1.633447
H	-1.67879	2.021647	1.701357

Table S89: Frequencies (cm^{-1}) of hexane + OOH → 3-hexyl + H_2O_2 transition state (R18), calculated at the M06-2X/aug-cc-pVTZ level of theory.

45	69	79	108	121	143	189	246
263	296	325	378	422	486	609	740
765	838	875	913	937	1024	1039	1058
1063	1076	1111	1156	1163	1193	1253	1293
1307	1319	1337	1378	1405	1408	1413	1419
1466	1474	1484	1495	1500	1505	1510	1513
3015	3035	3045	3049	3051	3058	3071	3083
3097	3115	3120	3128	3131	3791	-1815	

Table S90: Geometry (Å) of 3-hexyl radical, calculated at the M06-2X/aug-cc-pVTZ level of theory.

Atom	x	y	z
C	-3.38766	1.310277	-0.54538
H	-3.94039	1.860718	0.217482

H	-2.82914	2.032004	-1.1403
H	-4.11747	0.830578	-1.19933
C	-2.4699	0.275901	0.090712
H	-1.72701	0.774416	0.718086
H	-1.91233	-0.25531	-0.68409
C	-2.3649	-1.77711	1.554155
H	-1.29949	-1.58978	1.629925
C	-1.97174	-4.03856	2.581253
H	-1.07954	-3.68554	3.099848
H	-2.41996	-4.82811	3.183105
H	-1.65502	-4.47078	1.631955
C	-2.94806	-2.89049	2.348966
H	-3.84858	-3.25929	1.84677
H	-3.29704	-2.51247	3.321444
C	-3.23094	-0.73877	0.936327
H	-3.79369	-0.20198	1.716388
H	-4.00069	-1.22323	0.321545

Table S91: Frequencies (cm^{-1}) of 3-hexyl radical, calculated at the M06-2X/aug-cc-pVTZ level of theory.

34	52	126	142	217	263	298	372
387	471	724	770	861	910	921	1036
1050	1072	1089	1100	1152	1177	1248	1260
1274	1322	1337	1393	1412	1415	1440	1467
1480	1494	1501	1506	1509	1510	2955	2969
3014	3042	3042	3045	3056	3078	3112	3118
3123	3135	3169					

Table S92: Geometry (Å) of isohexane molecule, calculated at the M06-2X/aug-cc-pVTZ level of theory.

Atom	x	y	z
C	-0.02454	0.204155	-0.07848
H	0.436331	0.720914	0.764055
H	0.381232	0.635584	-0.99736
H	-1.0934	0.412493	-0.05211
C	0.268825	-1.2925	-0.02516
H	-0.21895	-1.70497	0.864576
C	1.769744	-1.53299	0.097585
H	2.290374	-1.12371	-0.77159

H	2.176811	-1.05088	0.986976
C	-0.28658	-2.02854	-1.24417
H	0.2104	-1.64517	-2.1432
H	-0.0107	-3.08526	-1.17015
H	1.998115	-2.59806	0.152917
C	-1.7966	-1.92866	-1.42415
H	-2.08006	-0.89046	-1.60666
H	-2.28932	-2.22513	-0.49368
C	-2.29709	-2.79667	-2.57163
H	-3.37567	-2.71266	-2.70056
H	-1.82368	-2.5055	-3.51063
H	-2.06057	-3.84717	-2.39609

Table S93: Frequencies (cm^{-1}) of isohexane molecule, calculated at the M06-2X/aug-cc-pVTZ level of theory.

94	114	184	221	262	269	333	382
444	454	746	832	866	915	932	975
982	1033	1069	1101	1179	1202	1208	1274
1300	1331	1357	1371	1400	1405	1415	1420
1479	1492	1496	1499	1506	1507	1515	1518
3017	3028	3038	3041	3042	3050	3059	3090
3105	3110	3118	3119	3127	3132		

Table S94: Geometry (Å) of isohexane + OOH → 2-isohexyl + H_2O_2 transition state (R19), calculated at the M06-2X/aug-cc-pVTZ level of theory.

Atom	x	y	z
C	-0.6885	-0.41318	-0.04249
H	0.410396	-0.31096	0.623961
O	1.540218	-0.22833	1.111792
O	2.373742	-0.37943	0.012624
H	2.579527	0.530462	-0.23603
C	-0.68363	0.81078	-0.92353
H	0.187656	0.816909	-1.58024
C	-0.4896	-1.70516	-0.80204
H	-1.3335	-1.82652	-1.49716
C	-1.76889	-0.4184	1.01121
H	-2.74294	-0.60382	0.543489
H	-1.60702	-1.19413	1.757915
H	-1.82282	0.542808	1.521909

H	-1.57432	0.817166	-1.56167
C	-0.35779	-2.9557	0.059187
H	0.400562	-2.77831	0.8257
H	-1.29724	-3.14801	0.581365
H	0.403986	-1.60423	-1.42529
H	-0.68966	1.727778	-0.33423
C	0.018507	-4.17607	-0.77063
H	0.98511	-4.03029	-1.25407
H	0.082468	-5.07277	-0.15542
H	-0.72061	-4.35801	-1.55275

Table S95: Frequencies (cm^{-1}) of isohexane + OOH → 2-isohexyl + H_2O_2 transition state (R19), calculated at the M06-2X/aug-cc-pVTZ level of theory.

39	86	108	118	139	190	208	219
226	257	328	361	395	418	467	577
750	828	871	924	942	986	995	1044
1075	1081	1118	1140	1194	1229	1264	1288
1327	1343	1397	1404	1409	1416	1419	1456
1461	1479	1486	1494	1500	1505	1508	1514
2984	3025	3028	3052	3054	3062	3093	3096
3103	3124	3127	3131	3140	3796	-1717	

Table S96: Geometry (Å) of 2-isohexyl radical, calculated at the M06-2X/aug-cc-pVTZ level of theory.

Atom	x	y	z
C	-2.26738	-1.85584	1.187773
H	-2.3502	-2.38568	0.222892
H	-1.19886	-1.65012	1.309307
C	-3.01824	-0.53187	1.069581
H	-3.01259	-0.03069	2.040876
H	-4.0648	-0.72196	0.823775
C	-2.71741	-2.75684	2.291927
C	-4.17662	-2.90962	2.559926
H	-4.67758	-1.94775	2.67521
H	-4.68358	-3.43053	1.733535
H	-4.3523	-3.4947	3.462677
C	-2.40561	0.375999	0.011485
H	-2.94847	1.316514	-0.07521
H	-1.36766	0.608337	0.254177
H	-2.41504	-0.10781	-0.96656

C	-1.81493	-3.8825	2.666443
H	-0.77051	-3.56747	2.689061
H	-2.07242	-4.29816	3.641587
H	-1.88108	-4.7079	1.941746

Table S97: Frequencies (cm^{-1}) of 2-isohexyl radical, calculated at the M06-2X/aug-cc-pVTZ level of theory.

56	65	102	145	196	244	288	332
392	462	736	797	852	914	932	990
991	1031	1072	1111	1126	1246	1256	1311
1322	1334	1397	1408	1409	1416	1468	1474
1483	1490	1494	1497	1502	1511	2938	2973
2977	3041	3044	3051	3065	3075	3083	3111
3112	3119	3120					

Table S98: Geometry (Å) of isohexane + OOH → 3-isohexyl + H_2O_2 transition state (R20), calculated at the M06-2X/aug-cc-pVTZ level of theory.

Atom	x	y	z
C	-0.65488	-0.45427	-0.12781
H	0.450345	-0.41033	0.578598
O	1.574989	-0.33917	1.025934
O	2.349992	-0.05463	-0.09007
H	2.419912	0.908869	-0.0725
C	-0.60695	0.813832	-0.94515
H	0.290236	0.770041	-1.57127
C	-0.54445	-1.77132	-0.84749
H	-1.41642	-1.89594	-1.50024
C	-0.44843	-2.95835	0.103614
H	0.436668	-2.87118	0.733164
H	-1.32287	-3.00384	0.754322
H	0.33006	-1.74045	-1.50333
C	-0.52584	2.043561	-0.04522
H	-0.43051	2.955282	-0.63518
H	-1.43297	2.129407	0.557265
H	0.318594	1.98183	0.642965
C	-1.82355	0.915394	-1.87319
H	-1.7933	1.8497	-2.43501
H	-1.85808	0.093082	-2.58621
H	-2.74625	0.902032	-1.28938
H	-1.41923	-0.43714	0.654766

H	-0.38775	-3.89777	-0.44378

Table S99: Frequencies (cm^{-1}) of isohexane + OOH → 2-isohexyl + H_2O_2 transition state (R20), calculated at the M06-2X/aug-cc-pVTZ level of theory.

50	68	81	117	138	177	222	236
272	307	347	392	412	438	465	589
761	849	895	926	932	968	986	1054
1071	1076	1108	1162	1183	1194	1201	1270
1296	1338	1367	1390	1406	1411	1416	1420
1455	1476	1491	1494	1504	1508	1515	1516
3023	3036	3040	3044	3060	3064	3081	3107
3114	3121	3131	3133	3144	3782	-1835	

Table S100: Geometry (Å) of 3-isohexyl radical, calculated at the M06-2X/aug-cc-pVTZ level of theory.

Atom	x	y	z
C	-2.62314	-2.45099	1.071595
H	-2.72095	-3.07451	0.190651
C	-1.96254	-1.12403	0.923092
H	-1.17465	-1.01219	1.677633
H	-2.67848	-0.32013	1.145852
C	-4.86699	-2.28998	2.150117
H	-5.34873	-2.69013	1.258282
H	-5.46205	-2.57268	3.020318
H	-4.87094	-1.20066	2.078149
C	-1.37198	-0.90927	-0.46487
H	-0.88176	0.05989	-0.54613
H	-0.63539	-1.68102	-0.69063
H	-2.15068	-0.95978	-1.22659
C	-2.79608	-2.2859	3.572613
H	-1.77313	-2.64509	3.688091
H	-2.7728	-1.19455	3.578171
H	-3.37332	-2.60879	4.439265
C	-3.42545	-2.80899	2.281936
H	-3.47636	-3.89965	2.34373

Table S101: Frequencies (cm^{-1}) of 3-isohexyl radical, calculated at the M06-2X/aug-cc-pVTZ level of theory.

58	94	199	255	265	282	284	326
396	467	565	778	816	907	927	964
1005	1052	1062	1085	1119	1181	1213	1278
1313	1332	1370	1391	1411	1415	1429	1472
1486	1497	1504	1507	1508	1513	2983	3015
3040	3048	3049	3054	3112	3119	3119	3122
3124	3136	3181					

Table S102: Geometry (Å) of 3-methylpentane molecule, calculated at the M06-2X/aug-cc-pVTZ level of theory.

Atom	x	y	z
C	0.410159	-1.29794	-0.10432
H	-0.17067	-1.40992	0.813218
C	-0.2451	-2.05327	-1.25453
H	0.306179	-1.86275	-2.18115
H	-0.16779	-3.12739	-1.06797
C	-2.29885	-2.61097	-2.57346
H	-1.75826	-2.42335	-3.50694
H	-2.09429	-3.65054	-2.30518
C	-3.79549	-2.43999	-2.80521
H	-4.03019	-1.46161	-3.22277
H	-4.17268	-3.19038	-3.49926
H	-4.34673	-2.54393	-1.86867
H	0.494643	-0.23289	-0.31691
H	1.414614	-1.67315	0.088926
C	-1.90361	-0.2309	-1.81894
H	-1.51528	0.411553	-1.02993
H	-1.37585	0.014813	-2.74464
H	-2.95455	0.020718	-1.95424
C	-1.71786	-1.70777	-1.4837
H	-2.25885	-1.91749	-0.55262

Table S103: Frequencies (cm^{-1}) of 3-methylpentane molecule, calculated at the M06-2X/aug-cc-pVTZ level of theory.

56	72	101	187	245	256	314	359
408	475	749	762	784	928	969	998
1008	1051	1079	1097	1125	1225	1272	1278
1324	1374	1393	1408	1412	1418	1470	1481
1486	1489	1504	1504	1512	1520	2953	2956
2971	3049	3049	3061	3066	3089	3118	3119

3125	3130	3135

Table S104: Geometry (Å) of 3-methylpentane + OOH → 3-methylpent-3-yl + H_2O_2 transition state (R21), calculated at the M06-2X/aug-cc-pVTZ level of theory.

Atom	x	y	z
C	-0.67809	-0.44762	-0.0242
H	0.44166	-0.40215	0.620816
O	1.588246	-0.31947	1.066861
O	2.354337	-0.0917	-0.06757
H	2.432261	0.870827	-0.09398
C	-0.61907	0.793992	-0.88783
H	0.225944	0.693074	-1.57331
C	-0.50685	2.106921	-0.12019
H	-0.31828	2.937789	-0.79869
H	-1.41935	2.333663	0.429445
H	0.309333	2.062804	0.604321
C	-0.53992	-1.72679	-0.82222
H	-1.39247	-1.79284	-1.5114
H	0.352055	-1.63668	-1.44695
C	-1.73811	-0.4442	1.049619
H	-2.73297	-0.48746	0.589602
H	-1.64249	-1.30097	1.714087
H	-1.69117	0.457948	1.657204
H	-1.51723	0.811847	-1.51921
C	-0.44907	-2.99826	0.012573
H	-1.38564	-3.2142	0.525576
H	-0.21481	-3.85439	-0.61826
H	0.338461	-2.90497	0.761597

Table S105: Frequencies (cm^{-1}) of 3-methylpentane + OOH → 3-methylpent-3-yl + H_2O_2 transition state (R21), calculated at the M06-2X/aug-cc-pVTZ level of theory.

55	59	88	102	120	152	194	219
257	264	348	364	404	430	484	565
761	768	821	916	982	995	1003	1046
1071	1078	1098	1145	1184	1216	1252	1276
1324	1374	1380	1402	1407	1414	1416	1459
1465	1478	1480	1495	1503	1506	1512	1521
3002	3007	3011	3046	3051	3068	3073	3102
3117	3123	3129	3134	3140	3786	-1709	

Table S106: Geometry (Å) of 3-methylpent-3-yl radical, calculated at the M06-2X/aug-cc-pVTZ level of theory.

Atom	x	y	z
C	0.383323	-1.27228	-0.12915
H	-0.30174	-1.2789	0.719804
C	-0.19384	-2.06878	-1.29556
H	0.49249	-1.9846	-2.15388
H	-0.20206	-3.13209	-1.04054
C	-1.57195	-1.67287	-1.72565
C	-2.27546	-2.63404	-2.63238
H	-1.80171	-2.6076	-3.62723
H	-2.10443	-3.64868	-2.26225
C	-3.77413	-2.40124	-2.79909
H	-3.98085	-1.46933	-3.32416
H	-4.22699	-3.20915	-3.37238
H	-4.26856	-2.35609	-1.82771
H	0.568023	-0.23408	-0.40286
H	1.330597	-1.69998	0.196728
C	-1.93299	-0.227	-1.77484
H	-1.56677	0.311925	-0.90201
H	-1.49839	0.26446	-2.65938
H	-3.01089	-0.08023	-1.82943

Table S107: Frequencies (cm^{-1}) of 3-methylpent-3-yl, calculated at the M06-2X/aug-cc-pVTZ level of theory.

56	72	101	187	245	256	314	359
408	475	749	762	784	928	969	998
1008	1051	1079	1097	1125	1225	1272	1278
1324	1374	1393	1408	1412	1418	1470	1481
1486	1489	1504	1504	1512	1520	2953	2956
2971	3049	3049	3061	3066	3089	3118	3119
3125	3130	3135					

Table S108: Geometry (Å) of heptane molecule, calculated at the M06-2X/aug-cc-pVTZ level of theory.

Atom	x	y	z

C	0.383658	-1.23306	-0.12684
C	1.862941	-1.52509	0.088277
H	2.431435	-1.33121	-0.82255
H	2.284446	-0.91129	0.883668
C	-0.22483	-2.08184	-1.23578
H	-0.08838	-3.14098	-0.99491
H	2.015204	-2.5719	0.35545
C	-1.70431	-1.80432	-1.46625
H	-1.84036	-0.74531	-1.70737
H	-2.25423	-1.97895	-0.53607
H	0.246993	-0.17589	-0.36913
H	-0.16656	-1.4086	0.801292
H	0.325409	-1.90757	-2.16599
C	-2.30901	-2.65461	-2.57546
H	-1.75905	-2.48042	-3.50585
H	-2.17283	-3.71383	-2.33476
C	-3.789	-2.3798	-2.80895
H	-4.33671	-2.55464	-1.8792
H	-3.92315	-1.32194	-3.04962
C	-4.37502	-3.23941	-3.92137
H	-5.43327	-3.03228	-4.07714
H	-3.85531	-3.05894	-4.86356
H	-4.27154	-4.29963	-3.68556

Table S109: Frequencies (cm^{-1}) of heptane molecule, calculated at the M06-2X/aug-cc-pVTZ level of theory.

75	98	102	154	158	249	272	275
308	424	489	733	740	778	854	900
932	948	1024	1038	1065	1093	1094	1107
1171	1209	1248	1268	1301	1326	1335	1343
1349	1377	1411	1416	1418	1422	1489	1489
1494	1499	1507	1510	1510	1514	1518	3029
3031	3039	3049	3051	3055	3056	3056	3062
3076	3088	3095	3123	3124	3133	3133	

Table S110: Geometry (Å) of heptane + OOH → 2-heptyl + H_2O_2 transition state (R22), calculated at the M06-2X/aug-cc-pVTZ level of theory.

C	0.383537	0.910814	0.161872
H	-0.86423	0.717722	-0.16886
H	0.637364	1.792653	-0.4275
O	-2.02034	0.382275	-0.30746

O	-2.19159	-0.60006	0.657694
H	-2.58225	-0.11756	1.397162
C	1.061422	-0.35424	-0.29451
H	0.557395	-1.21063	0.163848
H	0.947632	-0.46125	-1.37535
C	2.551051	-0.39737	0.062029
H	2.667117	-0.38477	1.149058
H	3.042097	0.505978	-0.31326
C	3.249893	-1.62671	-0.50342
H	3.162486	-1.62226	-1.59429
H	2.733341	-2.52818	-0.15943
C	0.334407	1.158632	1.644263
H	1.325151	1.413223	2.032635
H	-0.33366	1.984066	1.890208
H	-0.00277	0.263324	2.170233
C	4.720788	-1.70929	-0.11551
H	5.228957	-0.79737	-0.43988
H	4.802478	-1.73499	0.974285
C	5.414759	-2.92607	-0.7131
H	5.374755	-2.89735	-1.80291
H	6.462128	-2.97799	-0.41779
H	4.928649	-3.84756	-0.38948

Table S111: Frequencies (cm^{-1}) of heptane + OOH → 2-heptyl + H_2O_2 transition state (R22), calculated at the M06-2X/aug-cc-pVTZ level of theory.

42	45	62	87	113	132	138	152
209	230	252	346	362	400	428	465
604	723	751	830	868	900	926	946
996	1030	1054	1078	1084	1094	1111	1157
1167	1195	1239	1265	1294	1328	1332	1336
1372	1386	1407	1411	1415	1418	1465	1478
1486	1493	1495	1497	1503	1506	1515	3025
3031	3035	3041	3045	3049	3057	3069	3081
3087	3096	3100	3119	3124	3125	3796	-1826

Table S112: Geometry (Å) of 2-heptyl radical, calculated at the M06-2X/aug-cc-pVTZ level of theory.

C	-2.68522	-2.37592	0.817391
H	-1.58923	-2.4657	0.750215
H	-2.87913	-1.29593	0.816962

C	-3.16711	-2.96401	2.094863
H	-3.55572	-3.97429	2.082529
C	-3.30693	-3.00703	-0.42335
H	-4.39436	-2.90222	-0.37203
H	-3.10071	-4.08169	-0.41924
C	-2.79718	-2.40022	-1.72288
H	-3.00381	-1.32506	-1.72677
H	-1.70805	-2.50156	-1.76807
C	-3.41196	-3.03351	-2.96435
H	-3.20507	-4.10693	-2.95792
H	-4.49922	-2.93061	-2.91841
C	-2.88983	-2.41731	-4.25553
H	-3.33224	-2.88591	-5.13405
H	-3.11532	-1.35057	-4.29463
H	-1.80675	-2.52831	-4.32621
C	-2.81438	-2.33692	3.394881
H	-3.00545	-1.26038	3.376556
H	-3.37437	-2.7689	4.222627
H	-1.7472	-2.45517	3.626358

Table S113: Frequencies (cm^{-1}) of 2-heptyl radical, calculated at the M06-2X/aug-cc-pVTZ level of theory.

8	54	96	101	118	147	239	243
310	366	420	487	719	742	833	899
930	955	992	1027	1074	1082	1096	1112
1150	1179	1225	1255	1287	1312	1328	1333
1368	1402	1410	1413	1431	1469	1477	1486
1491	1492	1502	1505	1513	2961	2988	3012
3021	3032	3040	3046	3050	3050	3070	3085
3113	3121	3125	3192				

Table S114: Geometry (Å) of isoheptane molecule, calculated at the M06-2X/aug-cc-pVTZ level of theory.

C	0.362636	-1.27608	-0.07634
C	1.847094	-1.52403	0.156538
H	2.432775	-1.21118	-0.70912
H	2.215396	-0.97867	1.024872
C	-0.17728	-2.03667	-1.28136
H	-0.02132	-3.11033	-1.12988
H	2.041053	-2.58525	0.320035

C	-1.65633	-1.77156	-1.53439
H	-1.79371	-0.71827	-1.80592
H	-2.20667	-1.92048	-0.60002
H	0.184756	-0.20682	-0.21921
H	-0.20392	-1.56467	0.812745
H	0.408362	-1.7623	-2.16263
C	-2.2894	-2.64372	-2.61856
H	-2.18051	-3.68911	-2.31022
C	-3.77744	-2.33441	-2.74351
H	-4.29213	-2.4683	-1.79132
H	-3.92273	-1.29868	-3.06024
C	-1.59709	-2.47187	-3.96717
H	-1.62149	-1.42284	-4.27372
H	-0.5554	-2.78898	-3.93418
H	-2.09966	-3.05691	-4.73824
H	-4.25548	-2.97856	-3.48229

Table S115: Frequencies (cm^{-1}) of isoheptane molecule, calculated at the M06-2X/aug-cc-pVTZ level of theory.

61	86	126	152	232	251	262	305
313	414	444	482	728	790	841	910
918	935	969	978	1037	1060	1091	1103
1177	1202	1206	1255	1279	1319	1336	1342
1372	1384	1405	1409	1414	1423	1480	1489
1492	1498	1502	1505	1507	1513	1515	3021
3027	3033	3039	3042	3046	3047	3056	3069
3087	3108	3114	3115	3125	3126	3128	

Table S116: Geometry (Å) of isoheptane + OOH → 2-isoheptyl + H_2O_2 transition state (R23), calculated at the M06-2X/aug-cc-pVTZ level of theory.

Atom	x	y	z
C	0.383537	0.910814	0.161872
H	-0.86423	0.717722	-0.16886
H	0.637364	1.792653	-0.4275
O	-2.02034	0.382275	-0.30746
O	-2.19159	-0.60006	0.657694
H	-2.58225	-0.11756	1.397162
C	1.061422	-0.35424	-0.29451
H	0.557395	-1.21063	0.163848
H	0.947632	-0.46125	-1.37535
C	2.551051	-0.39737	0.062029

H	2.667117	-0.38477	1.149058
H	3.042097	0.505978	-0.31326
C	3.249893	-1.62671	-0.50342
H	3.162486	-1.62226	-1.59429
H	2.733341	-2.52818	-0.15943
C	0.334407	1.158632	1.644263
H	1.325151	1.413223	2.032635
H	-0.33366	1.984066	1.890208
H	-0.00277	0.263324	2.170233
C	4.720788	-1.70929	-0.11551
H	5.228957	-0.79737	-0.43988
H	4.802478	-1.73499	0.974285
C	5.414759	-2.92607	-0.7131
H	5.374755	-2.89735	-1.80291
H	6.462128	-2.97799	-0.41779
H	4.928649	-3.84756	-0.38948

Table S117: Frequencies (cm^{-1}) of isoheptane + OOH → 2-isoheptyl + H_2O_2 transition state (R23), calculated at the M06-2X/aug-cc-pVTZ level of theory.

42	45	62	87	113	132	138	152
209	230	252	346	362	400	428	465
604	723	751	830	868	900	926	946
996	1030	1054	1078	1084	1094	1111	1157
1167	1195	1239	1265	1294	1328	1332	1336
1372	1386	1407	1411	1415	1418	1465	1478
1486	1493	1495	1497	1503	1506	1515	3025
3031	3035	3041	3045	3049	3057	3069	3081
3087	3096	3100	3119	3124	3125	3796	-1826

Table S118: Geometry (Å) of 2-isoheptyl radical, calculated at the M06-2X/aug-cc-pVTZ level of theory.

C	-2.75302	-2.42902	-1.72889
H	-3.0253	-1.3737	-1.81678
H	-1.66237	-2.45946	-1.66278
C	-2.70894	-2.25934	3.336039
H	-3.101	-1.26506	3.598161
H	-2.89047	-2.9062	4.195356
H	-1.63107	-2.14775	3.208066

C	-3.3553	-2.78973	2.102252
C	-2.90137	-2.22365	0.795356
H	-3.25815	-1.18309	0.704077
H	-1.80874	-2.15097	0.798754
C	-3.34807	-2.99184	-0.44479
H	-3.06392	-4.04382	-0.34103
H	-4.4386	-2.9727	-0.52117
C	-3.21495	-3.18459	-2.9677
H	-2.78462	-2.76815	-3.8779
H	-2.92656	-4.23534	-2.9105
H	-4.30119	-3.14574	-3.06256
C	-4.73338	-3.34831	2.215949
H	-5.49581	-2.56099	2.115023
H	-4.94395	-4.08567	1.440152
H	-4.88857	-3.82179	3.185381

Table S119: Frequencies (cm^{-1}) of 2-isoheptyl radical, calculated at the M06-2X/aug-cc-pVTZ level of theory.

63	108	115	133	139	171	261	280
308	320	443	472	737	786	803	910
927	934	984	999	1034	1058	1090	1120
1134	1238	1244	1305	1308	1323	1336	1381
1400	1413	1416	1421	1464	1477	1483	1490
1493	1496	1502	1506	1515	2945	2970	2974
3038	3039	3050	3053	3071	3072	3077	3094
3119	3123	3125	3126				

Table S120: Geometry (Å) of isoheptane + OOH → 3-isoheptyl + H_2O_2 transition state (R24), calculated at the M06-2X/aug-cc-pVTZ level of theory.

Atom	x	y	z
C	0.016729	-0.50816	0.436901
H	0.649141	0.631164	0.578614
O	1.209995	1.701441	0.466194
O	1.016586	2.028278	-0.86911
H	0.235987	2.597175	-0.84861
C	-1.29984	-0.11909	-0.19143
H	-1.08245	0.315454	-1.17263
C	0.983113	-1.31779	-0.38398
H	0.524178	-2.28058	-0.64341

C	2.311377	-1.57347	0.31961
H	2.76236	-0.61401	0.580296
H	2.122391	-2.09823	1.259688
H	1.160893	-0.79787	-1.3313
C	-2.0322	0.914611	0.659322
H	-2.96668	1.219151	0.187846
H	-2.27379	0.494966	1.638506
H	-1.41886	1.801187	0.826101
C	-2.18559	-1.35259	-0.40553
H	-3.14151	-1.06194	-0.84252
H	-1.71633	-2.07758	-1.06879
H	-2.38637	-1.84233	0.549684
H	-0.08139	-0.84153	1.474124
C	3.272674	-2.3829	-0.53986
H	3.49251	-1.85838	-1.47058
H	4.21612	-2.56042	-0.02513
H	2.843919	-3.35254	-0.79825

Table S121: Frequencies (cm^{-1}) of isoheptane + OOH → 3-isoheptyl + H_2O_2 transition state (R24), calculated at the M06-2X/aug-cc-pVTZ level of theory.

29	46	70	83	114	128	148	216
247	256	280	310	351	389	404	432
499	593	730	824	856	912	931	935
966	984	1049	1070	1074	1088	1101	1161
1173	1194	1199	1254	1268	1322	1340	1348
1384	1398	1406	1411	1415	1423	1450	1466
1490	1494	1496	1506	1507	1510	1515	3006
3038	3040	3042	3044	3048	3052	3080	3089
3111	3115	3116	3121	3129	3134	3779	-1817

Table S122: Geometry (Å) of 3-isoheptyl radical, calculated at the M06-2X/aug-cc-pVTZ level of theory.

Atom	x	y	z
C	-2.77542	-2.41018	-1.70531
H	-3.0998	-1.36823	-1.76415
H	-1.69277	-2.38816	-1.56288
C	-2.76414	-2.17339	3.25164
H	-3.33447	-1.24364	3.309508
H	-2.86055	-2.68559	4.209228
H	-1.71535	-1.91649	3.102505

C	-3.1233	-2.35978	0.790818
H	-2.99233	-1.28287	0.771428
C	-3.42483	-3.05954	-0.48632
H	-3.10698	-4.10679	-0.41299
H	-4.51223	-3.10137	-0.65456
C	-3.11715	-3.13208	-3.00124
H	-2.65589	-2.65083	-3.86295
H	-2.77317	-4.16709	-2.97121
H	-4.19606	-3.1474	-3.16321
C	-4.75277	-3.42643	2.356128
H	-5.37559	-2.53048	2.397826
H	-5.13246	-4.06619	1.559526
H	-4.85856	-3.96032	3.301759
C	-3.28668	-3.03783	2.109695
H	-2.70819	-3.97053	2.084164

Table S123: Frequencies (cm^{-1}) of 3-isoheptyl radical, calculated at the M06-2X/aug-cc-pVTZ level of theory.

47	63	108	166	217	239	253	287
303	339	414	472	514	737	843	868
906	929	958	975	1056	1072	1101	1114
1139	1184	1205	1248	1265	1316	1322	1334
1380	1393	1413	1413	1433	1471	1489	1493
1494	1501	1506	1509	1514	2967	3007	3023
3039	3042	3047	3050	3085	3110	3114	3118
3121	3121	3125	3153				

Figure S4(a-b): Relative absolute deviations as functions of the temperature between rate constants calculated from explicit TST/Eckart calculations for all selected reactions and: (a) from the RC-TST/LER method where M062X reaction energies were used for the LER (b) from the RCT-TST/BHG method.

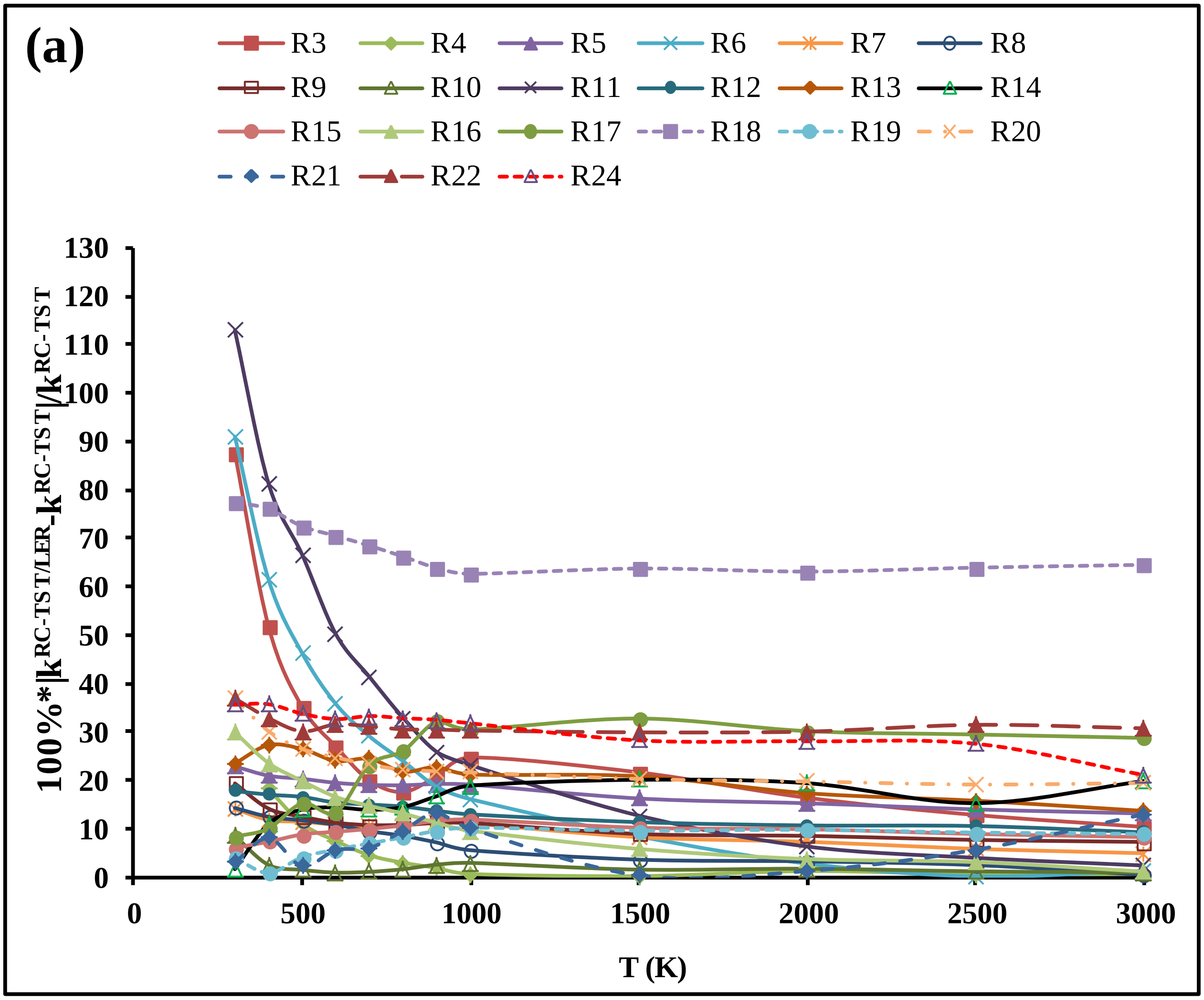

(a)
R3
R4
R5
R6
R7
R8
R9
R10
R11
R12
R13
R14
R15
R16
R17
R18
R19
R20
R21
R22
R24
100%*|k^RC-TST/LER-k^RC-TST|/k^RC-TST
T (K)
0
500
1000
1500
2000
2500
3000
130
120
110
100
90
80
70
60
50
40
30
20
10
0

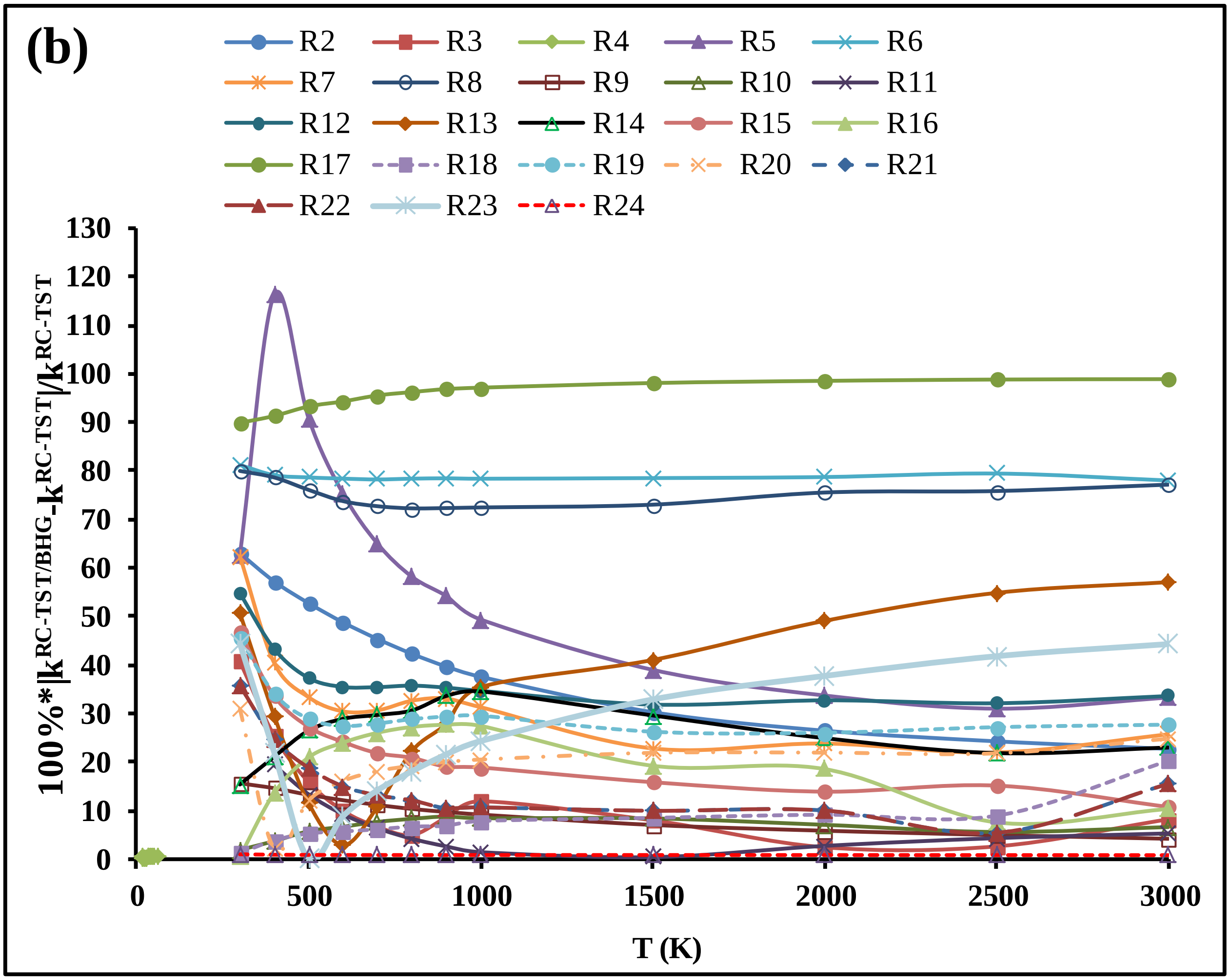
(b)
R2
R3
R4
R5
R6
R7
R8
R9
R10
R11
R12
R13
R14
R15
R16
R17
R18
R19
R20
R21
R22
R23
R24
100%*|k^RC-TST/BHG-k^RC-TST|/k^RC-TST
0
10
20
30
40
50
60
70
80
90
100
110
120
130
0
500
1000
1500
2000
2500
3000
T (K)
